# Lewis Superacidic Tellurenyl Cation‐Induced Electrophilic Activation of an Inert Carborane

**DOI:** 10.1002/chem.202103181

**Published:** 2021-09-28

**Authors:** Martin Hejda, Daniel Duvinage, Enno Lork, Antonín Lyčka, Zdeněk Černošek, Jan Macháček, Sergey Makarov, Sergey Ketkov, Stefan Mebs, Libor Dostál, Jens Beckmann

**Affiliations:** ^1^ Institut für Anorganische Chemie und Kristallographie Universität Bremen Leobener Straße 7 28359 Bremen Germany; ^2^ Department of General and Inorganic Chemistry University of Pardubice Studentská 573 532 10 Pardubice Czech Republic; ^3^ Faculty of Science University of Hradec Králové Rokitanského 62 500 03 Hradec Králové 3 Czech Republic; ^4^ Institute of Inorganic Chemistry Czech Academy of Sciences 250 68 Řež near Prague Czech Republic; ^5^ G. A. Razuvaev Institute of Organometallic Chemistry RAS 49 Tropinin St. 603950 Nizhny Novgorod Russian Federation; ^6^ Institut für Experimentalphysik Freie Universität Berlin Arnimallee 14 14195 Berlin Germany

**Keywords:** bond activation, boron, carboranes, Lewis superacids, tellurium

## Abstract

The aryltellurenyl cation [2‐(*t*BuNCH)C_6_H_4_Te]^+^, a Lewis super acid, and the weakly coordinating carborane anion [CB_11_H_12_]^−^, an extremely weak Brønsted acid (p*K*_a_=131.0 in MeCN), form an isolable ion pair complex [2‐(*t*BuNCH)C_6_H_4_Te][CB_11_H_12_], in which the Brønsted acidity (p*K*_a_ 7.4 in MeCN) of the formally hydridic B−H bonds is dramatically increased by more than 120 orders of magnitude. The electrophilic activation of B−H bonds in the carborane moiety gives rise to a proton transfer from boron to nitrogen at slightly elevated temperatures, as rationalized by the isolation of a mixture of the zwitterionic isomers 12‐ and 7‐[2‐(*t*BuN{H}CH)C_6_H_4_Te(CB_11_H_11_)] in ratios ranging from 62 : 38 to 80 : 20.

## Graphical abstract

**B−H bond activation**: The Lewis superacidic aryltellurenyl cation [2‐(*t*BuNCH)C_6_H_4_Te]^+^ increases the Brønsted acidity of the weakly coordinating carborane anion (CB_11_H_12_)^−^ by more than 120 orders of magnitude. Upon heating, the formal B−H bond is converted into an acidic N−H bond, while a Te−B bond is formed. A mechanism for the electrophilic substitution is proposed.

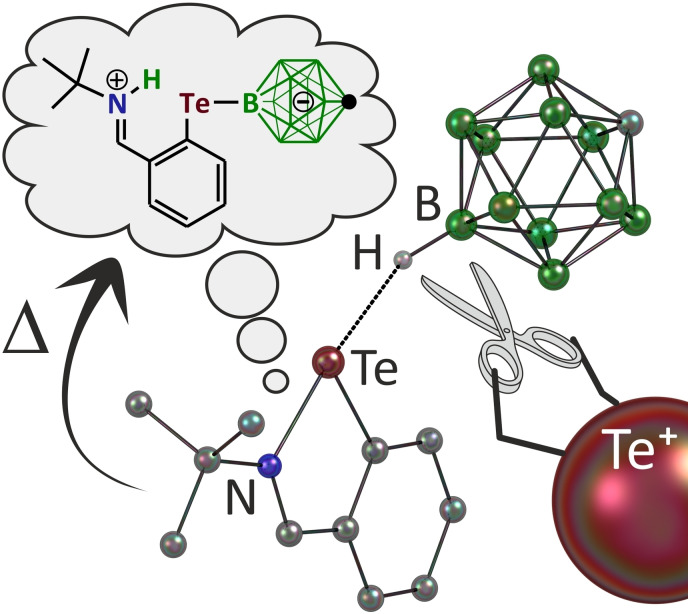

Main‐group elements mediating bond activation of small molecules and catalytic transformations have attracted considerable attention in recent years.^[1]^ That holds particularly for the cooperative reactivity of Lewis acids and Lewis bases that are restricted to form energetically favourable donor–acceptor complexes. Amongst those, the most prominent are arguably the frustrated Lewis pairs (FLPs), in which bulky substituents prevent the formation of stable (and unreactive) donor‐acceptor bonds. Besides FLPs, there is a growing number of regular, yet reactive Lewis pairs that are capable of activating small molecules.^[2]^

The aryltellurenyl cation, [2‐(*t*BuNCH)C_6_H_4_Te]^+^ (**I**), containing an imino donor functionality can be regarded as an intramolecular regular N→Te Lewis pair, however, the intramolecularly coordinating 2‐*tert*‐butyl‐iminomethylphenyl group compensates the electron deficiency at the tellurium atom only insufficiently (Figure 1).^[3]^ Despite the N‐donor coordination and the aromatic character of the five‐membered C_3_NTe ring, **I** is a highly electrophilic Lewis superacid that gives rise to ion pairs even with weakly coordinating anions (WCAs).^[3]^ These ion pairs [2‐(*t*BuNCH)C_6_H_4_Te][X] ([X]^−^=[O_3_SCF_3_]^−^, [SbF_6_]^−^ and [Al{OC(CF_3_)_3_}_4_]^−^) show significant Te⋅⋅⋅O and Te⋅⋅⋅F interactions in the solid state, whereas in nonpolar solvents the electrolytic dissociation remains incomplete. In an effort to obtain an essentially isolated [2‐(*t*BuNCH)C_6_H_4_Te]^+^ (**I**) cation, we turned our attention to an alternative WCA, namely, the *closo*‐carborane anion [CB_11_H_12_]^−^, which is known as robust entity with a very low reactivity.^[4]^ However, the salt metathesis reaction of 2‐(*t*BuNCH)C_6_H_4_TeCl (**II**)^[5]^ with Ag[CB_11_H_12_]^[6]^ provided again a contact ion pair [2‐(*t*BuNCH)C_6_H_4_Te][CB_11_H_12_] (**1**), which was isolated as yellow crystals in 99 % yield (Scheme 1, Figure 2).^[7]^ In the solid state, the dative N→Te bond (2.088(2) Å) of **1** is shorter than that of (*t*BuNCH)C_6_H_4_Te][O_3_SCF_3_] (2.113(1) Å), but longer than those of (*t*BuNCH)C_6_H_4_Te][SbF_6_] (2.076(2) Å) and (*t*BuNCH)C_6_H_4_Te][Al{OC(CF_3_)_3_}_4_] (2.051(4) Å). The cation and anion of **1** are associated by a prominent Te⋅⋅⋅H contact (2.550(1) Å) with the B−H functionality in the B12 position.^[8]^ As further reactivity studies showed, **1** is in fact only metastable and susceptible to further transformations at slightly elevated temperatures, both in CH_2_Cl_2_ and the solid state. Gentle heating of **1** in inert solvents produced a mixture of two isomeric dinuclear donor‐acceptor complexes [2‐(*t*BuNCH)C_6_H_4_Te⋅D][CB_11_H_12_] (**2 a**, D=12‐[2‐(*t*BuN{H}CH)C_6_H_4_Te]CB_11_H_11_ (**5 a**) and **2 b**, D=7‐[2‐(*t*BuN{H}CH)C_6_H_4_Te]CB_11_H_11_ (**5 b**)) in ratio 80 : 20,^[9]^ which were isolated as mixed orange crystals in 49 % yield (Scheme 1, Figure 2).[^7^, ^10^] In the solid‐state, the Te→Te bond of **2 a**(**2 b**) (3.034(1) Å) is substantially longer than that of [MesTe(TeMes_2_)](O_3_SCF_3_) (2.808(1) Å)^[11]^ or [MesTe(TeMes_2_)](SbF_6_) (2.765(1) Å)^[12]^ due to the additional N‐donation. The dative N→Te bond of **2 a**(**2 b**) (2.228(1) Å) is substantially longer than in **1**. As a result, the corresponding Te→Te vibration mode in Raman spectra of **2 a**(**2 b**) (122 cm^−1^) is considerably shifted bathochromically in comparison to ([MesTe(TeMes_2_)](SbF_6_) (143 cm^−1^) indicating a rather weak Te−Te bond.^[12]^ Although the donor–acceptor complex **2 a**(**2 b**) is virtually insoluble in CH_2_Cl_2_ and aromatic solvents, it very easily dissolves in THF via dissociation of the Te→Te bond (Figures S6–17 in the Supporting Information). Consequently, the formation of 1 equiv. of [2‐(*t*BuNCH)C_6_H_4_Te⋅THF][CB_11_H_12_] (**3**) is observed along with releasing of (1−*x*) equiv. of 12‐[2‐(*t*BuN{H}CH)C_6_H_4_Te]CB_11_H_11_ (**5 a**) and *x* equiv. of 7‐[2‐(*t*BuN{H}CH)C_6_H_4_Te]CB_11_H_11_ (**5 b**) from the mixture of **2 a** and **2 b** in molar ratio (1−*x*):*x* (Scheme 1).^[13]^ In the presence of the donor molecules THF and DMAP, the reaction of 2‐(*t*BuNCH)C_6_H_4_TeCl with Ag[CB_11_H_12_] directly afforded the mononuclear donor‐acceptor complexes [2‐(*t*BuNCH)C_6_H_4_Te⋅D][CB_11_H_12_] **3** (D=THF) and **4** (D=DMAP) as yellowish crystals in 95 % and 83 % yield (Scheme 1, Figure 2).[^7^, ^14^] It is worth noting that heating of the DMAP complex **4** in THF did not provide any hint of further transformation as the Lewis acidity is attenuated in comparison to **1**.^[15]^ On the other hand, prolonged heating of the THF complex **3** in THF quantitatively provided mixtures of the isomers **5 a** and **5 b** (Scheme 1). Thermodynamic measurements based on ^1^H NMR integration within transformation of **3** upon heating at various temperatures in [D_8_]THF proved its first‐order kinetics (Figures S28 and 29) with the following activation parameters: Δ*G* ^≠^_298_=118.4 kJ mol^−1^; Δ*H* ^≠^=114.9 kJ mol^−1^; Δ*S* ^≠^=−11.8 J mol^−1^ K^−1^. The most straightforward way providing the quantitative yield of the zwitterionic species **5 a** and **5 b** (in a ratio of 62 : 38) was heating of complex **3** in the solid state at 140 °C for 2.5 h, which effectively removes the THF (details in the Supporting Information). The formation of **5 a** and **5 b** (donors D in case of **2 a**(**2 b**)) may be rationalized by hydrogen transfer from the B−H functions in the B12 and B7 positions^[8]^ of the *closo*‐carborane anion [CB_11_H_12_]^−^ to the lone pair of the N atom in **1**, upon which a Te−B12(B7) bond is formed. This hydrogen transfer is facilitated by the cooperative reactivity of the intramolecular regular N→Te Lewis pair. While the Lewis acidic Te site reverses the formal polarity of the B−H bond from hydridic to protic, the Lewis basic N‐atom serves as a final proton acceptor within its gradual transfer from the Te atom to the N atom (for the DFT based mechanism, see below). Thus, the Lewis super acid [2‐(*t*BuNCH)C_6_H_4_Te]^+^ (**I**) has activated the *closo*‐carborane ion [CB_11_H_12_]^−^, which serves as a proton donor. In fact, such a bond arrangement gives a unique insight into a long unresolved issue of electrophilic activation of *closo*‐[CB_11_H_12_]^−^ (see below).^[4]^ This process is accompanied not only by a significant change in color from light‐yellow (**3**) to dark red, but also by a dramatic change in the *δ*(^125^Te) values^[15]^ reflecting the transition of the cationic Te^II^ site to neutral in the zwitterionic **5 a**(**5 b**) by the Te−B12(B7) bond formation. Consequently, the boron atom at the B12 position of the free [CB_11_H_12_]^−^ anion of **3** resonating in ^11^B NMR at −7.3 ppm is high‐field shifted upon formation of Te−B12 bond in compound **5 a** to −11.5 ppm. While the unsubstituted B12 atom in case of **5 b** containing Te−B7 bond resonates slightly more low‐field at −5.8 ppm, the signal of boron atom at the B7 position is shifted to high field from *δ*(^11^B)=−13.7 ppm in **3** to *δ*(^11^B)=−17.3 ppm. Interestingly, both Te−B12 and Te−B7 atoms in **5 a** and **5 b** are in ^11^B NMR significantly low‐field shifted (Δ*δ* ∼7 ppm) in comparison to analogously monoiodo substituted derivatives of *closo*‐carbadodecaborate.^[16]^ In the solid state **5 a**(**5 b**) features a weak intramolecular hydrogen bond of the type N−H⋅⋅⋅Te (N−H: 0.860(2) Å, H⋅⋅⋅Te (2.568(1) Å, N⋅⋅⋅Te: 3.320(1) Å), in which Te serves as unprecedented hydrogen bond acceptor. This bonding situation is a result of the **5 a**(**5 b**) formation mechanism (see below) enforcing the *E* configuration of the protonated imino C*H*=N*H* ^+^ moiety for both products, which is manifested by observation of typical ^3^ *J*(^1^H,^1^H) values (acquired in CD_2_Cl_2_) of 17.3 and 17.4 Hz, respectively. Although the intramolecular hydrogen bond of the type N−H⋅⋅⋅Te was found in the solid state, the coupling constant ^1^ *J*(^15^N,^1^H)=85.3 Hz for both **5 a** and **5 b** in the solution spectra lies in range for protonated imines.^[17]^ Similarly, despite the fact that both **5 a** and **5 b** contain such bonding interaction, FTIR is showing no shift of the N−H bond *ν* vibration in the solid state sample (**5 a**: 3244 cm^−1^ **5 b**: 3295 cm^−1^) in comparison to adducts **2 a** and **2 b** (3244 cm^−1^ and 3297 cm^−1^) having no N−H⋅⋅⋅Te interaction. Finally, deprotonation of **5 a**(**5 b**) was achieved upon addition of triethylamine, which afforded a mixture of [Et_3_NH][12‐{2‐(*t*BuNCH)C_6_H_4_Te}CB_11_H_11_] (**6 a**) and [Et_3_NH][12‐{2‐(*t*BuNCH)C_6_H_4_Te}CB_11_H_11_] (**6 b**) in the same molar ratio as given by parent compounds **5 a**(**5 b**), which was isolated as orange oil in quantitative yield (Scheme 1). The deprotonation was shown to have a significant effect on the shielding of the nitrogen atom, as the signal with value of *δ*(^15^N)=−172.1 ppm acquired in [D_8_]THF for **5 a**(**5 b**) is shifted to low field for **6 a** and **6 b** up to −35.4 and −32.8 ppm, respectively. Despite the assessable lone pair of the N atom in the **6 a**(**6 b**) for coordination of Te atom, we can conclude that in this case no N→Te interaction is present, as *δ*(^15^N) for these compounds approaches the value of *δ*(^15^N) for the unsubstituted parent Schiff base, namely (*t*BuNCH)C_6_H_5_ (−24.3 ppm; [D_8_]THF).

**Figure 1:**
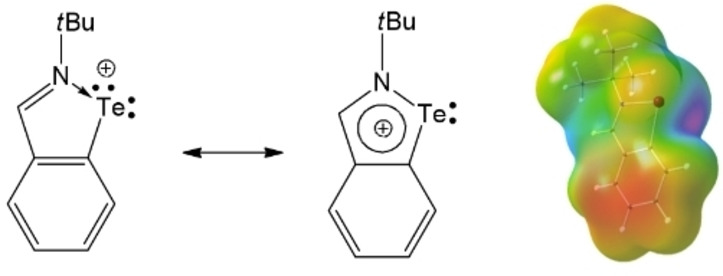
Resonance formula representations and electrostatic potential (ESP) of the Lewis superacid [2‐(*t*BuNCH)C_6_H_4_Te]^+^ (**I**).^[3]^

**Scheme 1:**
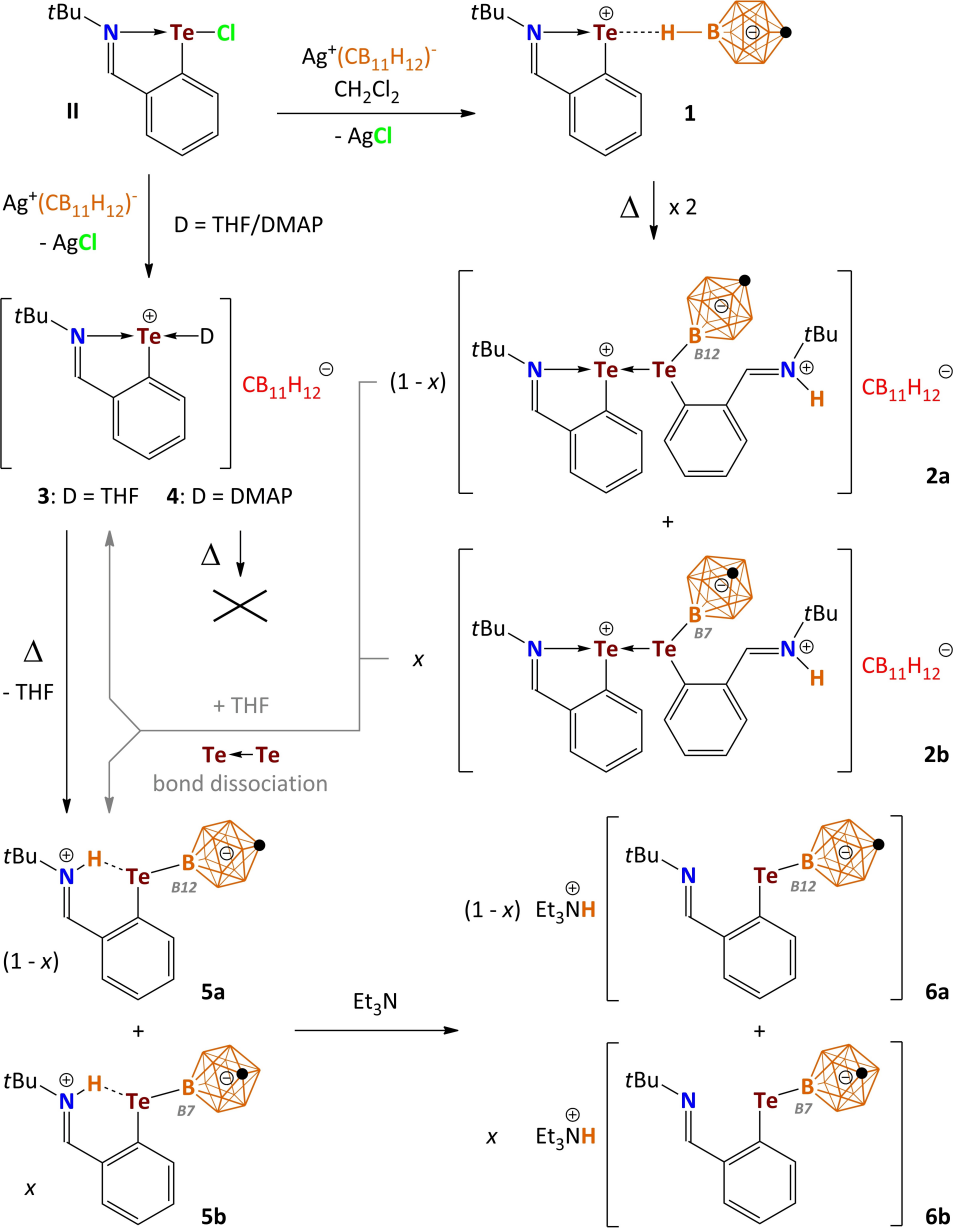
Reaction of 2‐(*t*BuNCH)C_6_H_4_TeCl (**II**) with Ag[CB_11_H_12_] and further transformations.

**Figure 2:**
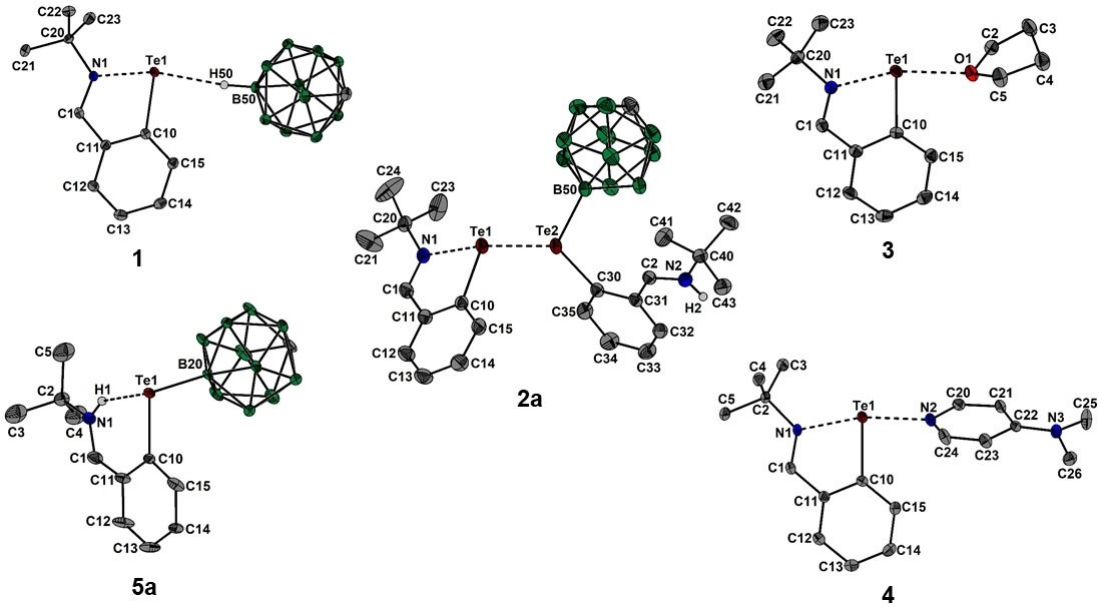
Molecular structures of **1**, **2 a**(**2 b**), **3**, **4** and **5 a**(**5 b**) showing 50 % probability ellipsoids and the atomic numbering scheme. For **2 a**(**2 b**), **3** and **4**, the [CB_11_H_12_]^−^ anion is omitted for clarity.

In an effort to shed light on the activation of the *closo*‐carborane anion by the Lewis superacidic aryltellurenyl cation, two contact ion pairs, namely, **1** resembling the B12−H⋅⋅⋅Te connectivity found in the solid state, and the by 7.6 kJ mol^−1^ less stable isomer **1’** featuring a B7−H⋅⋅⋅Te connectivity, were fully optimized in the gas phase (Figure 3a). The calculated B⋅⋅⋅Te distances of **1** (2.858 Å) and **1’** (2.867 Å) are significantly shorter than the experimentally observed distance of **1** (3.410(2) Å). As a result of the contact to adjacent Te atom, the B−H bond lengths of **1** (1.234) and **1’** (1.229) are substantially longer than those of the free *closo*‐carborane anion (1.194 and 1.193 Å). The B−H stretching upon Te coordination is accompanied by a dramatic increase of the Brønsted acidity. As expected for a weakly coordinating anion, the calculated p*K*_a_ value^[18]^ of the free *closo*‐carborane (131.0) in MeCN is extremely high, rendering it an extremely weak acid. The p*K*_a_ values of **1** (7.4) and **1’** (7.2) are dramatically smaller by more than 120 units. These p*K*_a_ values compare well with that of HCl (7.9) in MeCN.^[18]^ Thus, the Lewis superacidic aryltellurenyl cation, [2‐(*t*BuNCH)C_6_H_4_Te]^+^ (**I**) induces an electrophilic activation of the 12‐ or 7‐B−H bond in the *closo‐*carborane ion^[4]^ which seems to be instrumental for the bond activation and the proton transfer to the N atoms of **5 a** and **5 b**. The proton transfer from **1’** to **5 a** is associated with an energy gain of 193.3 kJ mol^−1^ and most likely proceeds via a concerted wagging motion involving the transition state **TS** with double triangular arrangement of N, Te and B12−H, which accounts for an activation barrier of 118.1 kJ mol^−1^ (Figure 3a). Such a value of activation barrier is in great agreement with the experimental one (Δ*G* ^≠^_298_=118.4 kJ mol^−1^) as discussed above. The AIM bond topology of **TS** reveals a curved Te−H(B) bond path, indicating the onset of Te−B bond formation (Figure 3b).[^19^, ^20^, ^21^] With an electron density (ED, *ρ*(**r**)) of 0.59 eÅ^‐3^ and considerably negative total energy over ED ratio (*H*/*ρ*(**r**)) of −0.38 a.u., covalent bonding aspects of the Te−H contact in the TS are much higher than in **1** (*ρ*(**r**)=0.34, *H*/*ρ*(**r**)=−0.21 a.u.) and **5 a** (*ρ*(**r**)=0.23, *H*/*ρ*(**r**)=−0.14 a.u.). This is supported by the NCI, which shows a ring‐shaped and red‐colored NCI basin enclosing the Te−H and Te−B bonding axes, in contrast to the disc‐shaped and blue‐colored NCI basins in **1** and **5** (Figure 3d). In accordance with a H(Te)B arrangement, the ED within the corresponding ELI−D basin is distributed over the atoms as follows: H=53 %, Te=26 %, and B=22 %, compared to the bisynaptic contact mode in **1** (H=16 %, Te=4 %, B=80 %). In **5 a**, only the N (77 %) and now protic H (23 %) atoms contributions are relevant, supporting a coordinative Te⋅⋅⋅H bonding mode (Figure 3c).

**Figure 3:**
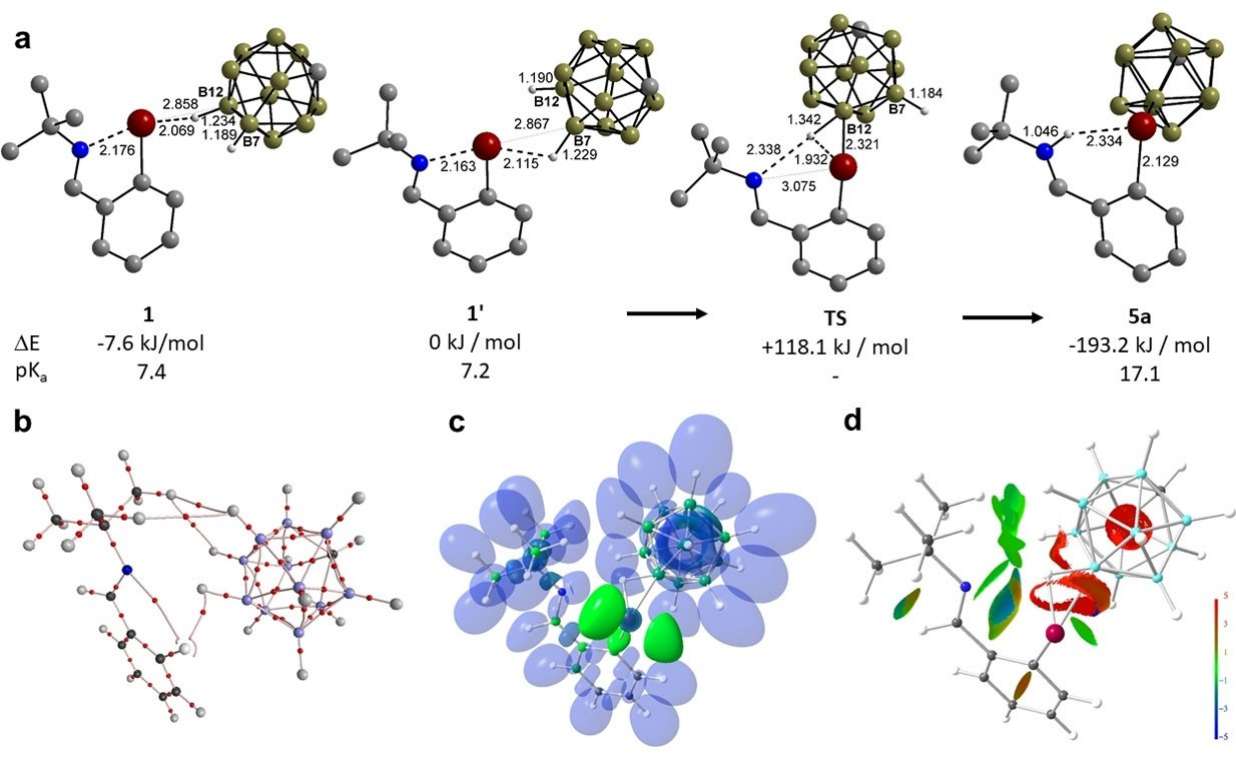
a) Calculated gas‐phase structures, relative energies and p*K*_a_ values of **1**, **1’**, **TS**, and **5 a**. b) The atoms‐in‐molecules (AIM) topology, c) iso‐surface representation of the electron localizability indicator (ELI−D) and d) the noncovalent interaction (NCI) index of the **TS**.

In summary, the aryltellurenyl cation [2‐(*t*BuNCH)C_6_H_4_Te]^+^ (**I**), and the weakly coordinating *closo*‐carborane anion [CB_11_H_12_]^−^ give rise to a metastable contact ion pair [2‐(*t*BuNCH)C_6_H_4_Te][CB_11_H_12_] (**1**), in which the B−H bond in the 12‐ or 7‐position of *closo*‐[CB_11_H_12_]^−^ is activated by the proximity of a Lewis superacidic cation, the Brønsted acidity of the *closo*‐carborane is extremely increased, and this triggers proton transfer and the formation of 12‐[2‐(*t*BuN{H}CH)C_6_H_4_Te]CB_11_H_11_ (**5 a**) and 7‐[2‐(*t*BuN{H}CH)C_6_H_4_Te]CB_11_H_11_ (**5 b**) in ratios ranging from 62 : 38 to 80 : 20.^[9]^ The contact ion pair **1** can be regarded as a snapshot of the first step of the electrophilic substitution of *closo*‐[CB_11_H_12_]^−^, which is poorly understood in general.^[4]^ Our proposed mechanism is in full agreement with the theoretically calculated mechanism of the methylation of *closo*‐[CB_11_H_12_]^−[22]^ involving the formation of three‐center bonded intermediate [MeBH]^+^. We are currently investigating the utility of **I** for the activation of small molecules.

## Conflict of interest

The authors declare no conflict of interest.

## Supporting information

As a service to our authors and readers, this journal provides supporting information supplied by the authors. Such materials are peer reviewed and may be re‐organized for online delivery, but are not copy‐edited or typeset. Technical support issues arising from supporting information (other than missing files) should be addressed to the authors.

Supporting Information
