# Supplementary material for: Lewis Superacidic Tellurenyl Cation‐Induced Electrophilic Activation of an Inert Carborane
Source: Chemistry. 2021 Sep 28;27(59):14577–81. doi: 10.1002/chem.202103181 (PMC8596995; doi:10.1002/chem.202103181)
Supplement: Supplementary file 1 — Supporting Information [file CHEM-27-14577-s001.pdf]

Chemistry–A European Journal

Supporting Information

Lewis Superacidic Tellurenyl Cation-Induced Electrophilic Activation of an Inert Carborane

Martin Hejda,* Daniel Duvinage, Enno Lork, Antonín Lyčka, Zdeněk Černošek, Jan Macháček, Sergey Makarov, Sergey Ketkov,* Stefan Mebs,* Libor Dostál, and Jens Beckmann*

Contents

Experimental details	**S2**
Synthesis of [2-(*t*BuNCH)C_6H_4Te][$CB_{11}H_{12}$] (**1**), NMR data and heating in CD_2Cl_2	**S5**
Synthesis of compounds **2a** and **2b**, NMR data and vibration spectra	**S10**
Evidences proving dissociation of Te→Te^+ bond of **2a/2b** upon dissolving in THF	**S15**
Synthesis of [2-(*t*BuNCH)C_6H_4Te·THF][$CB_{11}H_{12}$] (**3**) and NMR data	**S18**
Synthesis of [2-(*t*BuNCH)C_6H_4Te·DMAP][$CB_{11}H_{12}$] (**4**) and NMR data	**S21**
Synthesis of a mixture of **5a/5b**	**S25**
Kinetics of transformation of compound **3** into compounds **5a/5b**	**S27**
NMR data and vibration spectra of mixture of **5a/5b** in various solvents	**S31**
Proof that protonated imino bond CH=NH^+ in **5a/5b** has *E*- configuration in both cases	**S41**
Synthesis of a mixture of **6a/6b**, NMR data and UV/Vis spectra	**S42**
Proof that **5a** and **5b** regioisomers crystallizes together in one crystal lattice	**S48**
Crystallography	**S49**
Computational methodology and results	**S52**
pK_a values calculations	**S66**
Additional references	**S71**

Experimental details

General procedures. All air- and moisture-sensitive manipulations were carried out under argon (99.999 %) using Schlenk tube techniques enhanced with utilization of rubber septa and cannula for transfer of liquids and for their filtration (via filtration cannulas). All glassware was annealed in vacuum prior to use. All solvents for reactions were dried using Pure Solv–Innovative Technology equipment. The starting compounds: Et_3N (≥99%) and DMAP (4-*N,N*-dimethylaminopyridine, ≥99%) were obtained from Sigma-Aldrich. Et_3N was dried by refluxing with sodium under argon gas and then distilled. Compound **II** (2-(*t*BuNCH)C_6H_4TeCl) was synthesized according to our recently published procedure (M. Hejda, E. Lork, S. Mebs, L. Dostál, J. Beckmann, *Eur. J. Inorg. Chem.* **2017**, 3435-3445.).
Ag($CB_{11}H_{12}$) was synthesized by converting of commercially (Katchem Ltd., Czech Republic) available trimethylammonium salt of 1-carbadodecaborate by sodium hydride in THF with subsequent treatment with aqueous solution of silver nitrate followed by recrystallization from hot benzene and then dried in vacuo for 2 hours at 70 °C (K. Shelly, D. C. Finster, Y. J. Lee, W. R. Sheidt, C. A. Reed, *J. Am. Chem. Soc.* **1985**, *107*, 5955.).

Solution NMR spectroscopy. ^{1}H, ^{11}B, ^{13}C, ^{15}N and ^{125}Te NMR spectra were recorded on a Bruker Avance 500 MHz, a Bruker Avance III HD 400 MHz or a JEOL 600 MHz spectrometers, using a 5 mm tunable broad-band probe. Appropriate chemical shifts in ^{1}H and ^{13}C NMR spectra are given relative to the residual signals of the solvent [CD_2Cl_2: δ(^{1}H) = 5.32 ppm and δ(^{13}C) = 54.0 ppm; $CDCl_3$: δ(^{1}H) = 7.24 ppm and δ(^{13}C) = 77.23 ppm and THF-d_8: δ(^{1}H) = 3.58 ppm and δ(^{13}C) = 67.57 ppm], ^{11}B NMR spectra were related to external secondary standard $B(OMe)_3$ [δ(^{11}B) = 18.1 ppm], ^{15}N NMR spectra were related to external neat nitromethane [δ(^{15}N) = 0.0 ppm]. ^{125}Te NMR chemical shifts are referenced to external

$CDCl_3$ solution of Ph_2Te_2 [$\delta(^{125}Te)$ = 422 ppm relative to Me_2Te]. THF-d_8 (99.5% D) was dried by refluxing with potassium under argon atmosphere followed by vacuum transfer prior to use. Other NMR solvents were dried by following procedures: $CDCl_3$ (99.8% D), CD_2Cl_2 (99.96% D) and $C_2D_2Cl_4$ (99.5% D) were dried by staying over molecular sieves (3Å) for a couple of days and then degassed by three cycles *freeze-pump-thaw.* NMR samples were prepared under argon and measured in annealed NMR tubes sealed by means of Young valve (PTFE) or in annealed flame-sealed NMR tubes. The full assignment of all signals in all measured NMR spectra was managed with the help of various techniques including ^{1}H, ^{11}B, $^{11}B\{^{1}H\}$, $^{11}B\{^{1}H\}$-$^{11}B\{^{1}H\}$ COSY, $^{13}C\{^{1}H\}$ APT, ^{1}H-^{1}H COSY, ^{1}H-^{13}C HSQC and ^{1}H-^{13}C HMBC. The absolute configuration on protonated imino bond ($CH=NH^+$) of discussed species in solution was confirmed as an *E*- in all cases by the help of ^{1}H-^{1}H NOESY (mixing time d8 = 0.2 – 0.8 s). ^{15}N NMR chemical shifts and $^{1}J(^{15}N,^{1}H)$ were obtained from ^{1}H-^{15}N HMBC spectra (value of cnst13 = 4 Hz).

Solid-state NMR spectroscopy. ^{125}Te MAS NMR spectrum was recorded on a Bruker Avance 500 MHz spectrometer equipped with MAS DVT 500SB BL3.2 N-P/H probe with 3.2 mm zirconia rotor, with MAS rate 15 and 20 kHz. ^{125}Te MAS spectrum was acquired using Hahn-Echo pulse sequence with P(1) = 3.5 μs, P(2) = 7,0 μs, D(1) = 30 s, L(1) = 2, RF power 180 W (–22.55 dB). Chemical shifts are referenced relative to Me_2Te at 0 ppm, using the isotropic resonance of solid $Te(OH)_6$ (site 1) at 692.2 ppm as a secondary reference.

UV/Vis spectroscopy. Electronic absorption spectra were run on a Black-Comet C-SR-100 concave grating spectrometer with help of dip probe (optical pathway 1 mm) in Schlenk tubes (region 200 - 1080 nm) in dried THF (drying procedure discussed in General procedures).

IR and Raman spectroscopy. FT-IR spectra of solid samples were recorded using single-bounce diamond ATR crystal on Nicolet iS50. FT-Raman spectra of solid samples or liquids were recorded in the range 4000 – 100 cm^{-1} with a Nicolet iS50 equipped with iS50 Raman module (excitation laser 1064 nm).

Experimental setup for measurement of kinetics of reactions. Flame-sealed NMR tubes with samples were heated in a stirred oil bath and the temperature was monitored with external high-precision digital thermometer TD 110 (VWR). After a given time, the NMR tube was rapidly cooled down in –70 °C bath in order to immediately stop the reaction. Then the NMR sample was analyzed by ^{1}H NMR with NS = 16 at 294 K.

Synthesis of [2-(tBuNCH)C_6H_4Te][$CB_{11}H_{12}$] (1).

2-(tBuNCH)C_6H_4TeCl **(II)** (303 mg, 0.94 mmol) and Ag($CB_{11}H_{12}$) (235 mg, 0.94 mmol) were loaded into a Schlenk tube under argon atmosphere, CH_2Cl_2 was added (15 mL) and the reaction mixture was stirred for 15 min at RT with exclusion of light. The resulting ivory suspension was filtered and the obtained light orange filtrate was immediately evaporated *in vacuo* without any heating under formation of light orange polycrystals. Isolated yield 403 mg (>99 %); m.p. 100 °C (decomp. under formation of compounds **5a** and **5b** with dark red color). Suitable single-crystals were obtained after recrystallization from small amount of CH_2Cl_2 during one day after storing at 6 °C. Compound **1** is highly soluble in CH_2Cl_2 and donor solvents (Et_2O, THF), however virtually insoluble in aliphatic and aromatic solvents.

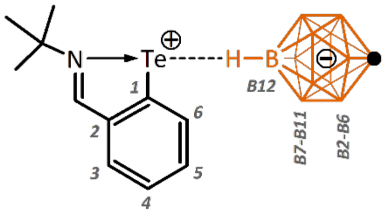

^{1}H NMR (500.20 MHz, CD_2Cl_2) δ (ppm): 1.77 [9H, s, (CH_3)$_3$C]; 0,87–2,22 [11H, m, HC$B_{11}$$H_{11}^-$]; 2.45 [1H, s, *H1*-$CB_{11}H_{11}^-$]; 7.71 [1H, dt, Ar(C4)-*H*]; 7.74 [1H, dt, Ar(C5)-*H*]; 8.32 [1H, dd, Ar(C6)-*H*]; 8.35 [1H, dd, Ar(C3)-*H*]; 9.50 [1H, s, C*H*=N]. **^{11}B NMR** (160.48 MHz, CD_2Cl_2) δ (ppm): –16.2 [5B, d, *B2–B6*), $^1J(^{11}B,^1H)$ = 152.3 Hz]; -13.7 [5B, d, *B7-B11*), $^1J(^{11}B,^1H)$ = 135.3 Hz]; –7.9 [1B, d, *B12*, $^1J(^{11}B,^1H)$ = 129.8 Hz]. **^{13}C{^{1}H} NMR** (125.78 MHz, CD_2Cl_2) δ (ppm): 32.8 [s, (*C*H_3)$_3$C-]; 52.7 [s, *C*$B_{11}H_{12}^-$]; 66.7 [s, (CH_3)$_3$*C*-]; 129.1 [s, Ar-*C4*]; 133.0 [s, Ar-*C6*]; 133.8 [s, Ar-*C5*]; 134.2 [s, Ar-*C3*]; 138.1 [s, qC, Ar-*C2*]; 144.5 [br. s, qC, Ar-*C1*(*ipso*)]; 164.2 [s, *C*H=N]. **^{125}Te NMR** (126.24 MHz, CD_2Cl_2) δ: not observed.

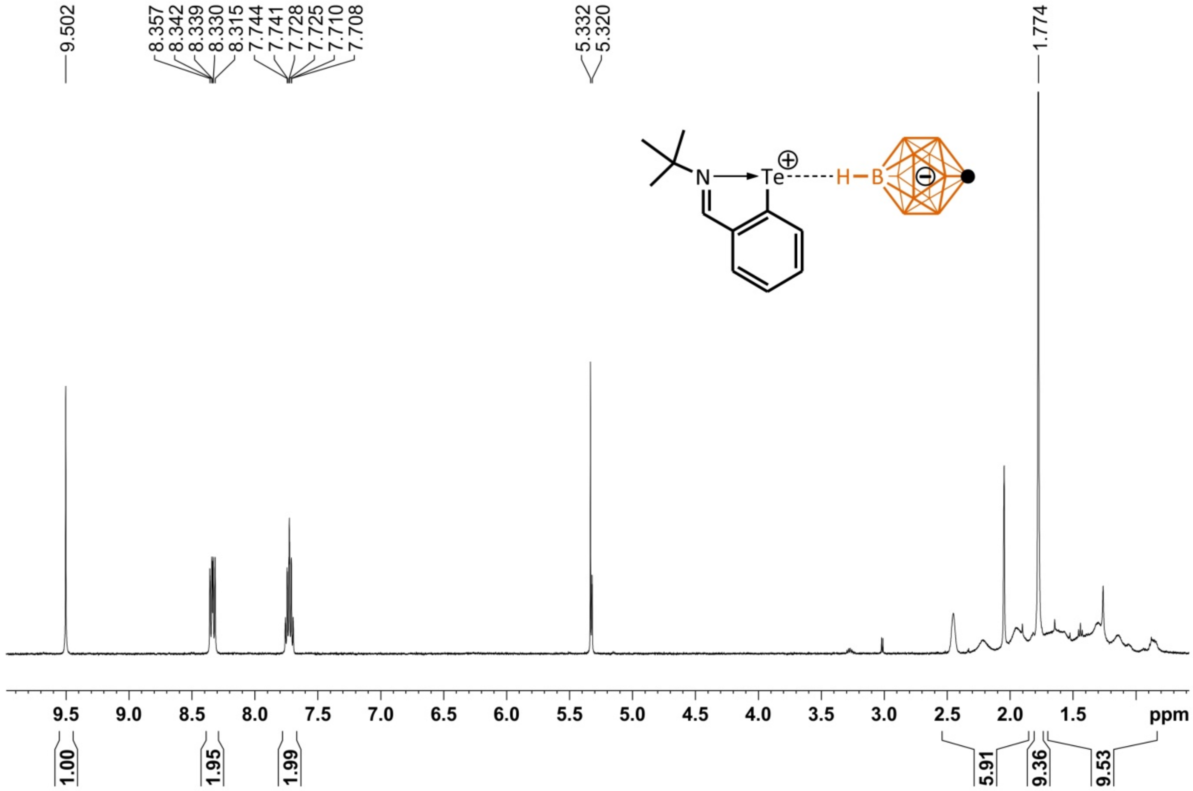

Figure S1. [1]**H NMR** spectrum of **1** (500.20 MHz, CD_2Cl_2, 294 K).

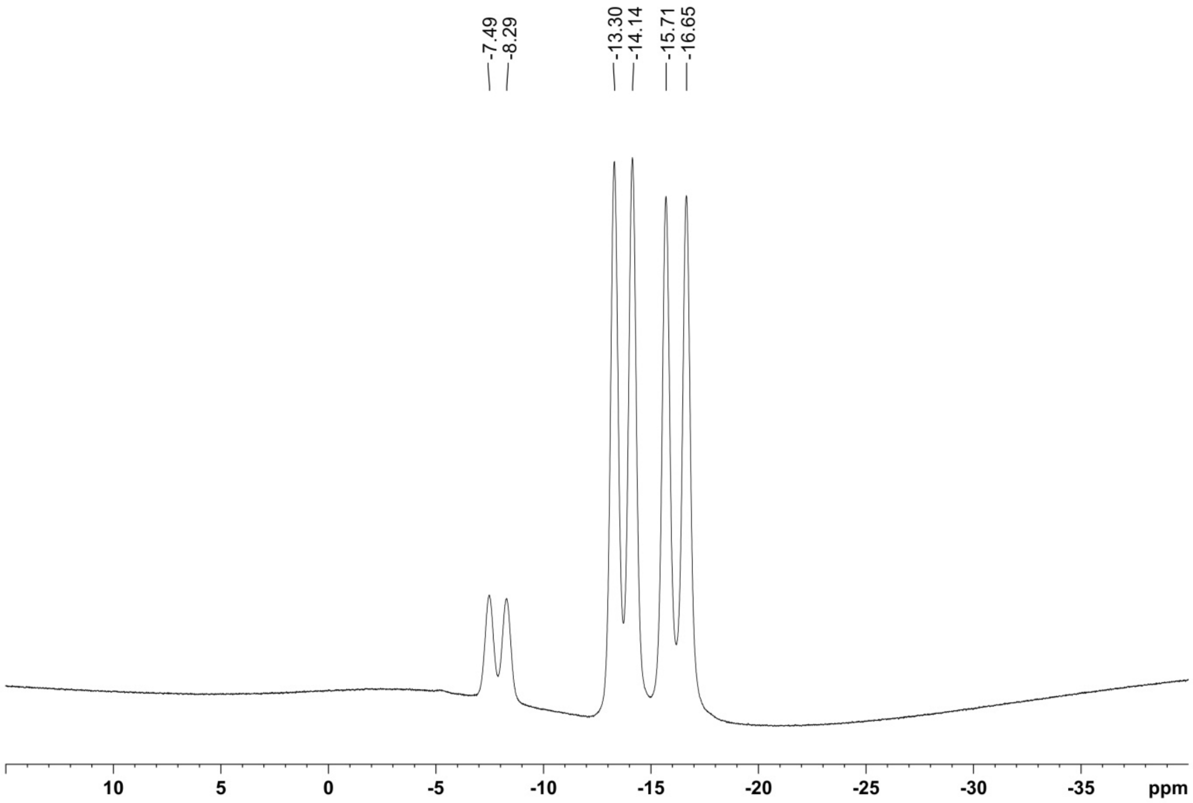

Figure S2. [11]**B NMR** spectrum of **1** (160.48 MHz, CD_2Cl_2, 294 K).

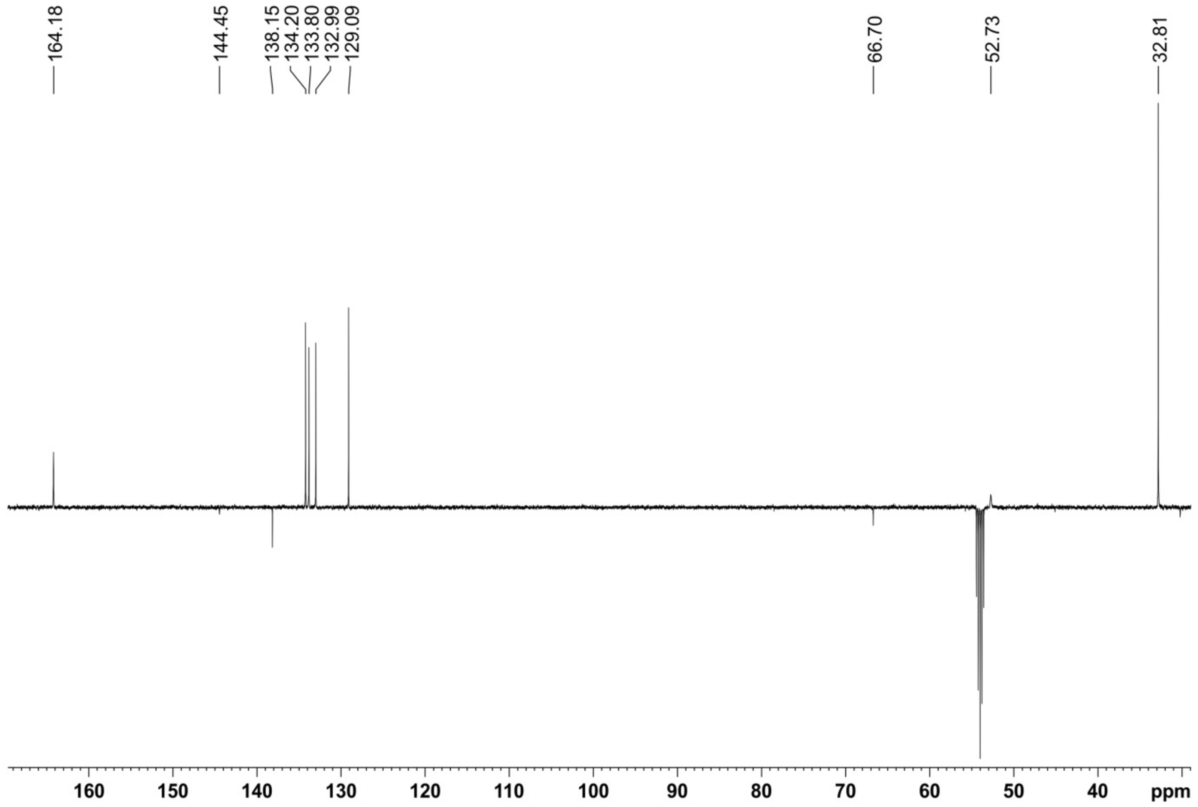

Figure S3. **$^{13}C\{^{1}H\}$ APT NMR** spectrum of **1** (125.78 MHz, CD_2Cl_2, 294 K).

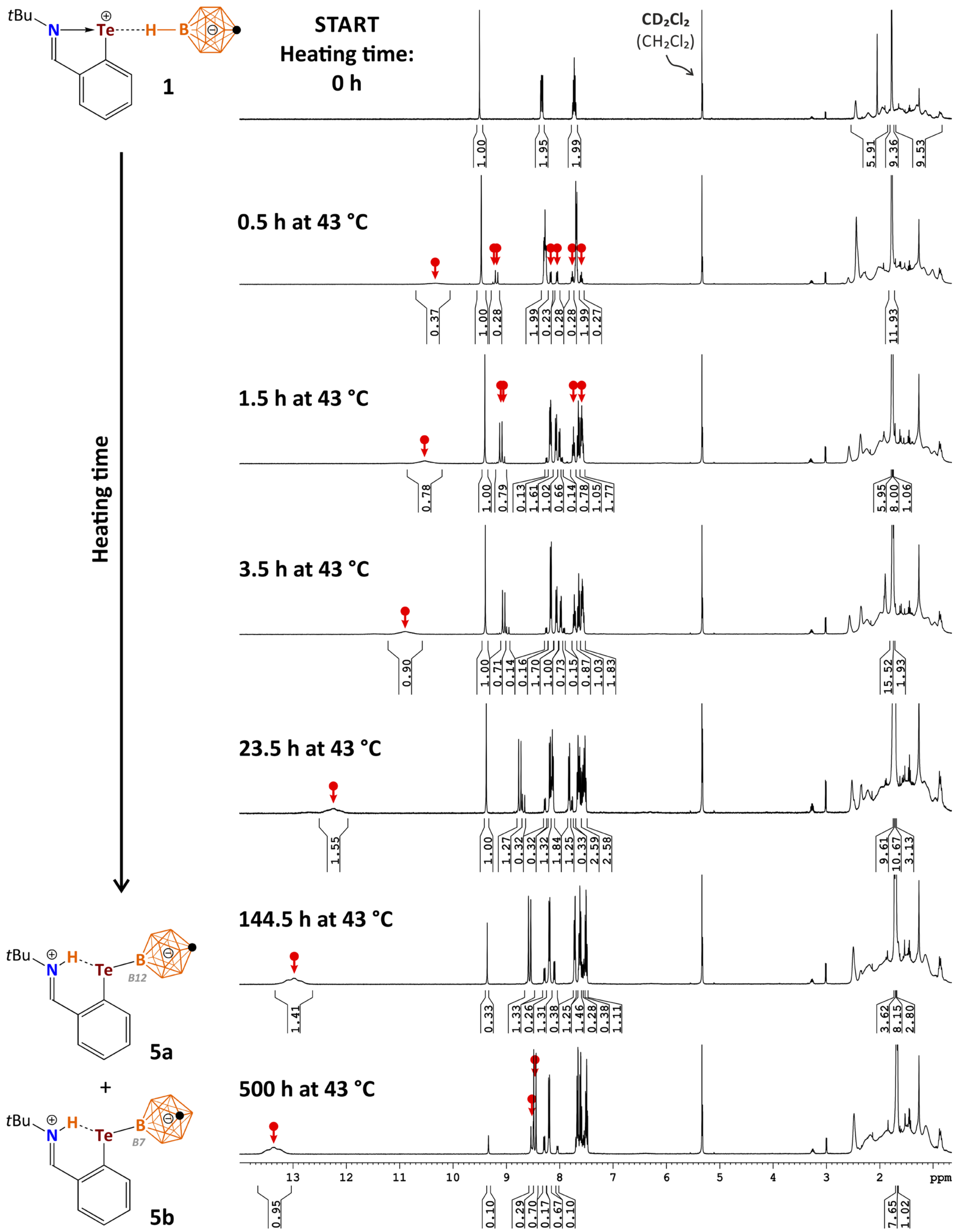

Figure S4. Stacked plot of ^{1}H NMR spectra (500.20 MHz, 294 K) showing transformation of the starting compound **1** into a mixture of compounds **5a** and **5b** when heated in CD_2Cl_2. Red arrows correspond to characteristic signals of products **5a** and **5b**. Formation of **5a** and **5b** is, however, affected (slowed down) by formation of complexes **2a** and **2b** with Te→Te$^+$ interaction.

Table S1. Experimentally obtained relative concentration of compound **1** vs **5a+5b** obtained by integration in ^{1}H NMR spectra, specifically by integration of intensity of C*H*=NH$^+$ doublets of both **5a** and **5b** and C*H*=N singlet of compound **1** (see Figure S4 above).

time [hours]	time [s]	relative [**1**]	ln(relative [**1**])
0,5	300	0,7888	-0,2372
1,5	5400	0,5685	-0,5647
3,5	12600	0,5349	-0,6257
23,5	84600	0,3741	-0,9831
144,5	520200	0,2053	-1,5831
264,5	952200	0,1603	-1,8306
500	1800000	0,0894	-2,4147
716	2577600	0,0853	-2,4611

Transformation of compound **1** in non-coordinating CD_2Cl_2 *does not proceed via expected law of 1st order kinetics* (see Figure S5, bottom). The reaction seems to be complicated by *in situ* formation of complexes **2a+2b** with dative Te→Te$^+$ bond. In other words, the more of both **5a+5b** is formed, the more and more becomes transformation of **1** into **5a+5b** inhibited and thus conversion of resting (unreacted) molecules of compound **1** is more and more difficult.

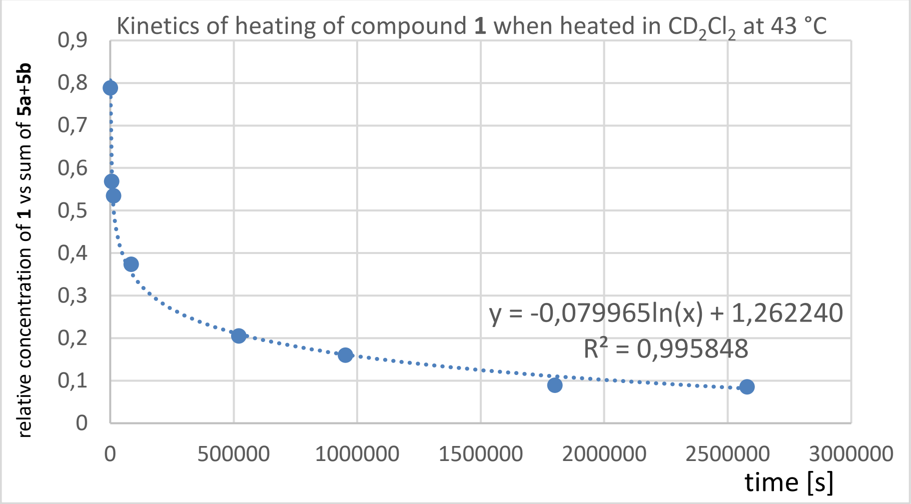

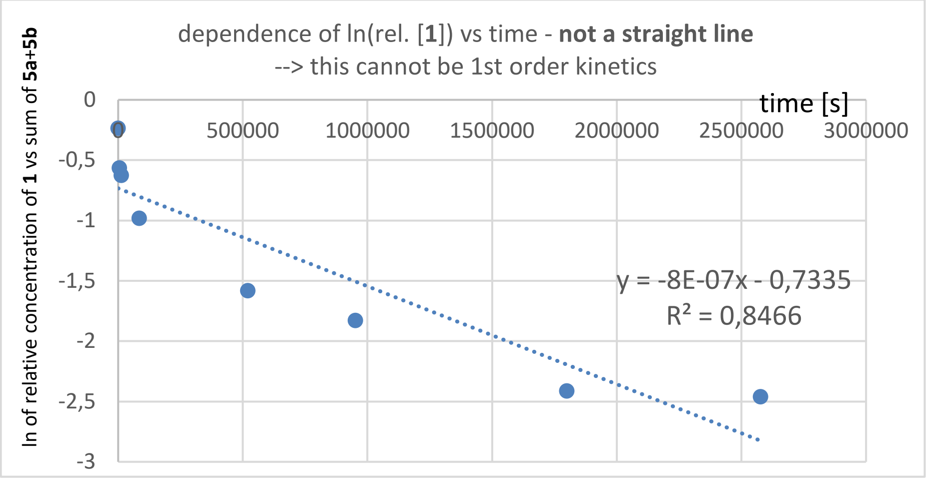

Figure S5. Plot of experimental data obtained for heating of compound **1** in CD_2Cl_2 at 43 °C (measured values can be found in Table S1 above).

Synthesis of [2-(*t*BuNCH)C_6H_4Te·D][$CB_{11}H_{12}$]

(2a, D = 12-[2-(tBuN{H}CH)C_6H_4Te]$CB_{11}H_{11}$ (5a)

and 2b, D = 7-[2-(*t*BuN{H}CH)C_6H_4Te]$CB_{11}H_{11}$ (5b)).

During an attempt to crystallize compound **1** (356 mg, 0.83 mmol) from oversaturated CH_2Cl_2 solution, the solution was heated up over the temperature of the boiling point of CH_2Cl_2 (at 70 °C) in a flame-sealed glass ampoule. Compounds **2a** and **2b** were obtained as orange single-crystals by very slow cooling of this solution in isolated yield 174 mg (49 %), m.p. 152 °C (decomp.).

Compounds **2a** and **2b** are slightly soluble in CH_2Cl_2 and virtually insoluble in aliphatic and aromatic solvents, however, they are highly soluble in THF under formation of blood-red solution. This color change is caused by dissociation of a dative Te→Te^+ bond under formation of blood-red **5a** and **5b** next to yellow **3** (**Figure S6**).

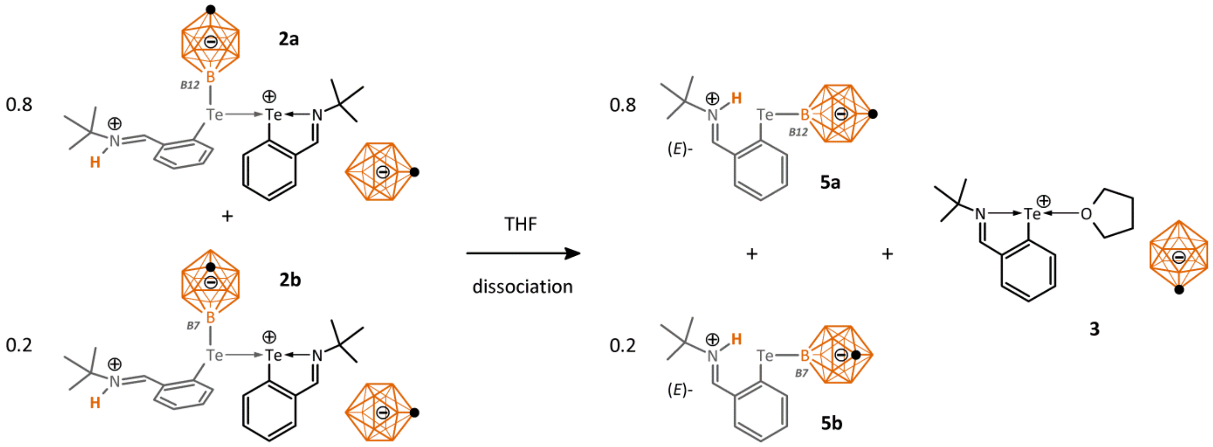

Figure S6. Te→Te^+ bond dissociation of compounds **2a**/**2b** upon dissolving in THF.

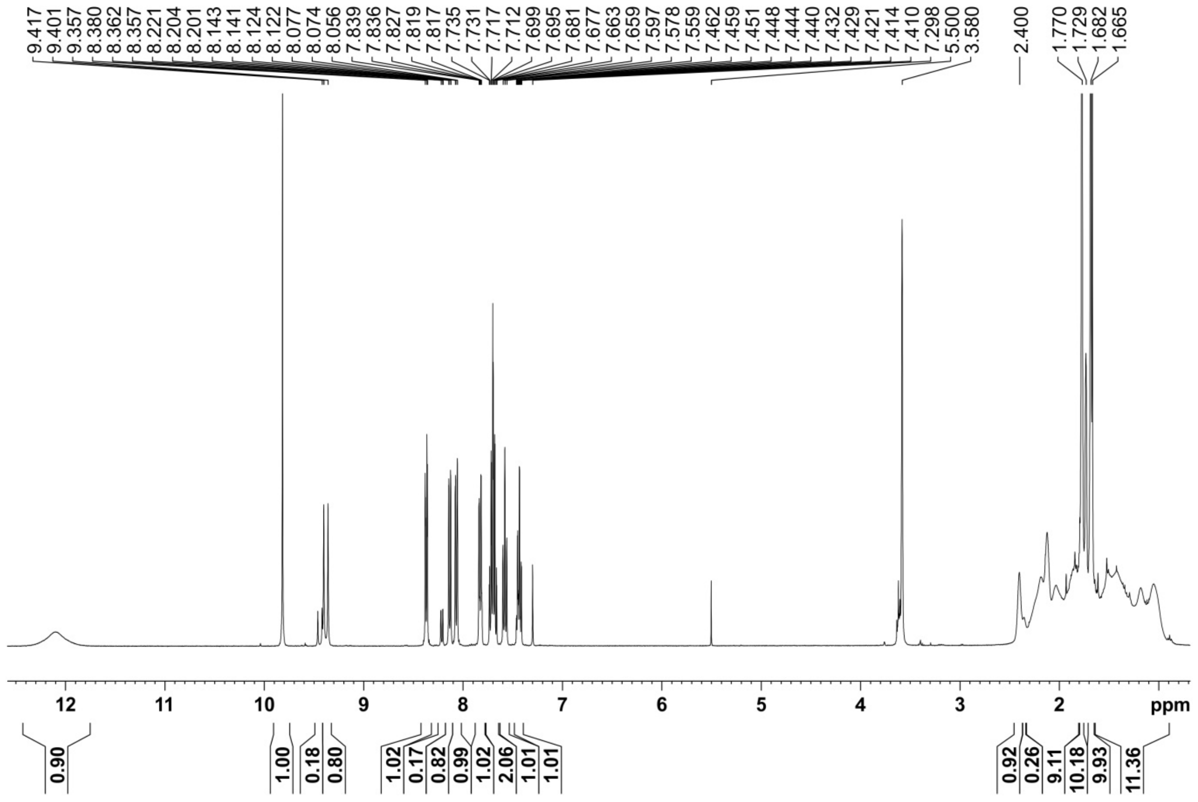

Figure S7. **^{1}H NMR** spectrum of a mixture of **2a** and **2b** (400.13 MHz, THF-d_8, 294 K).

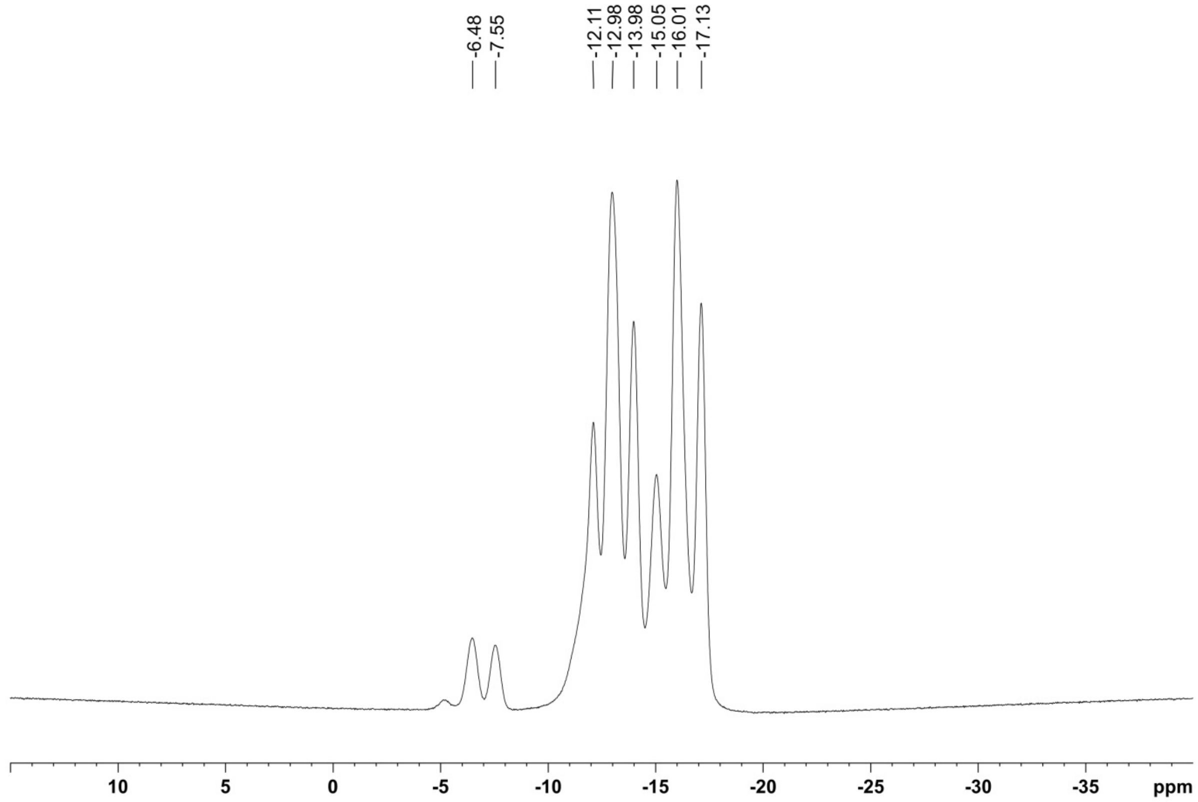

Figure S8. **^{11}B NMR** spectrum of a mixture of **2a** and **2b** (128.38 MHz, THF-d_8, 294 K).

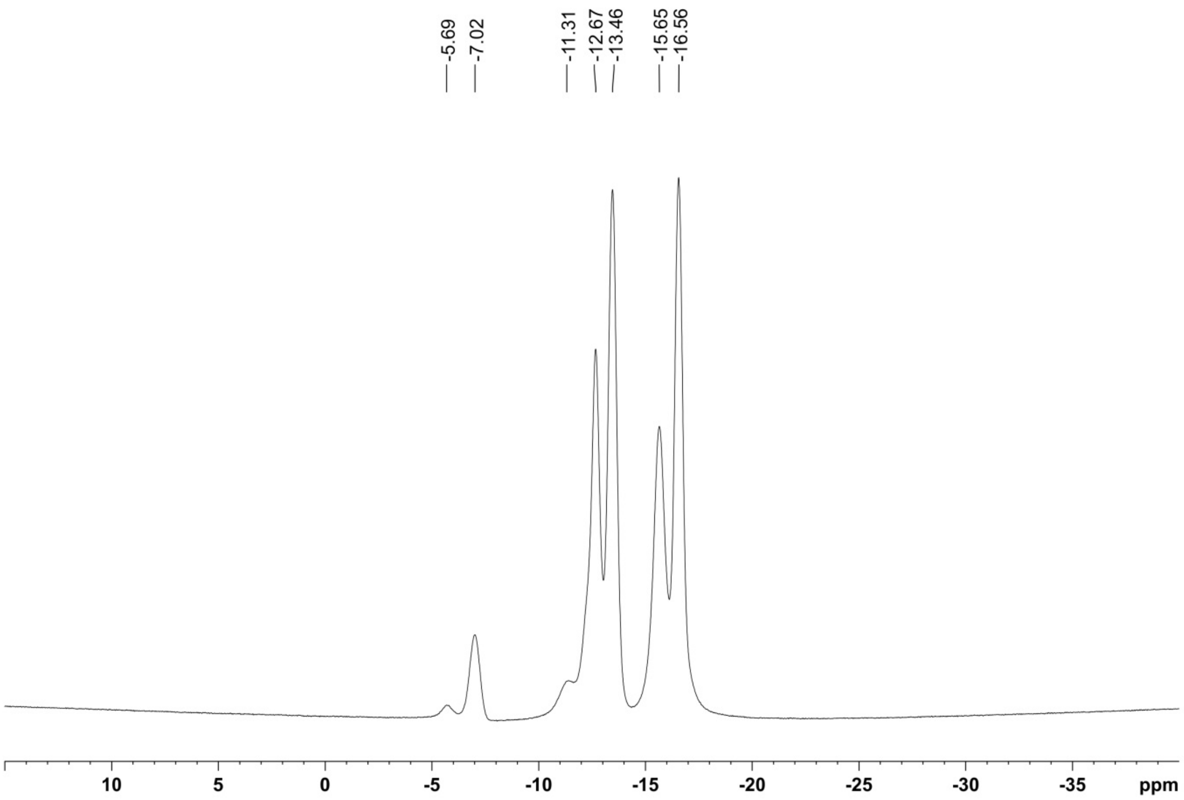

Figure S9. **$^{11}B\{^1H\}$ NMR** spectrum of a mixture of **2a** and **2b** (128.38 MHz, THF-d_8, 294 K).

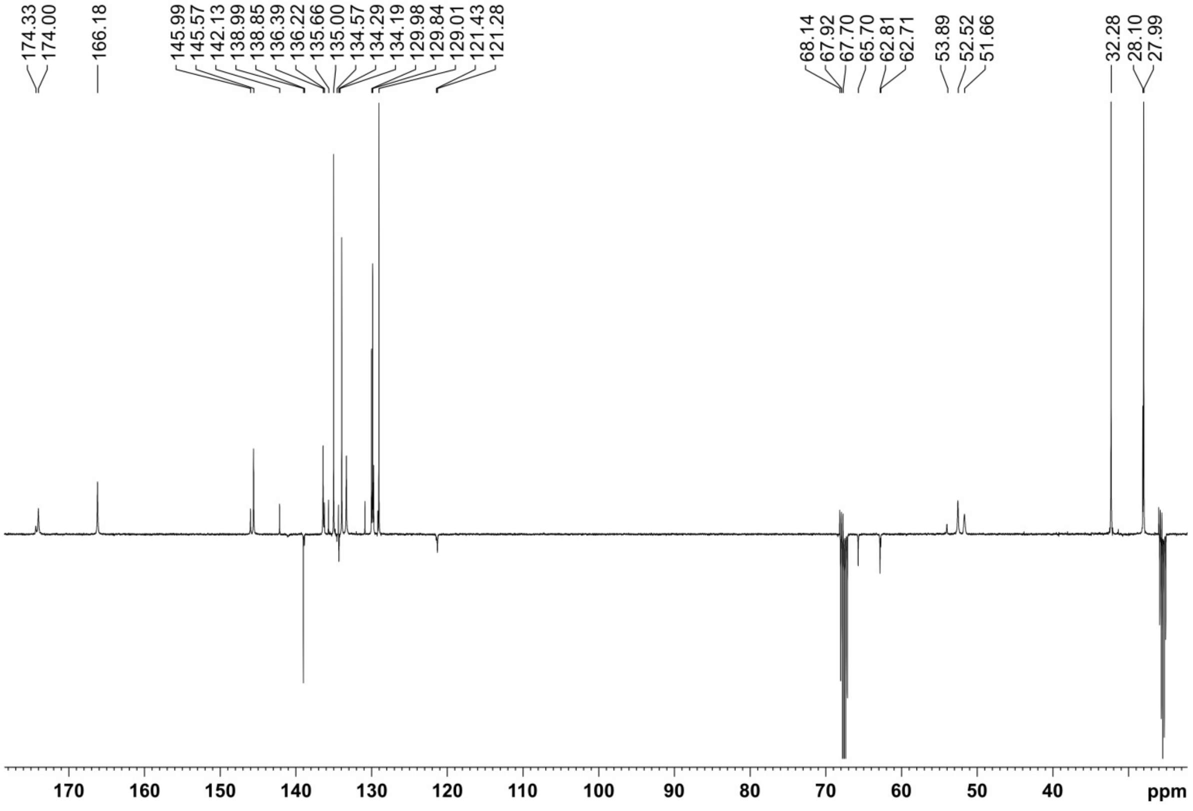

Figure S10. **$^{13}C\{^1H\}$ APT NMR** spectrum of a mixture of **2a** and **2b** (100.61 MHz, THF-d_8, 294 K).

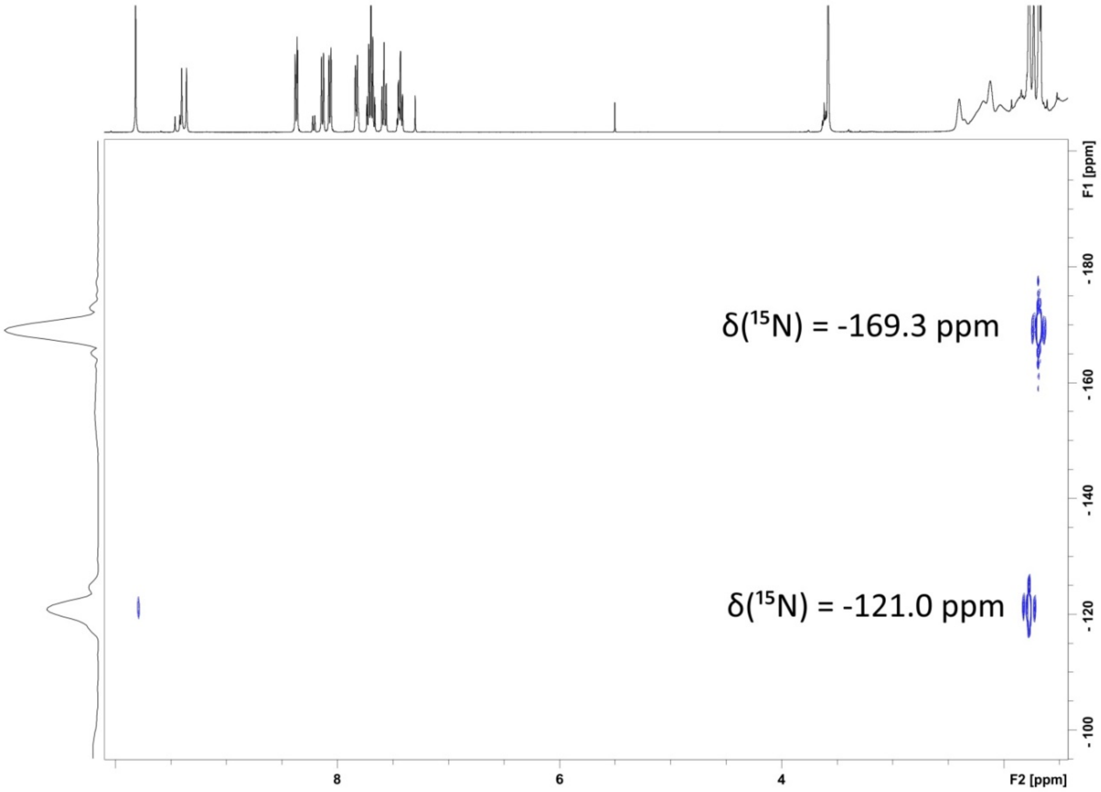

Figure S11. **^{1}H-^{15}N HMBC** NMR spectrum of a mixture of **2a** and **2b** (400.13 MHz, THF-d_8, cnst13 = 4 Hz, 294 K).

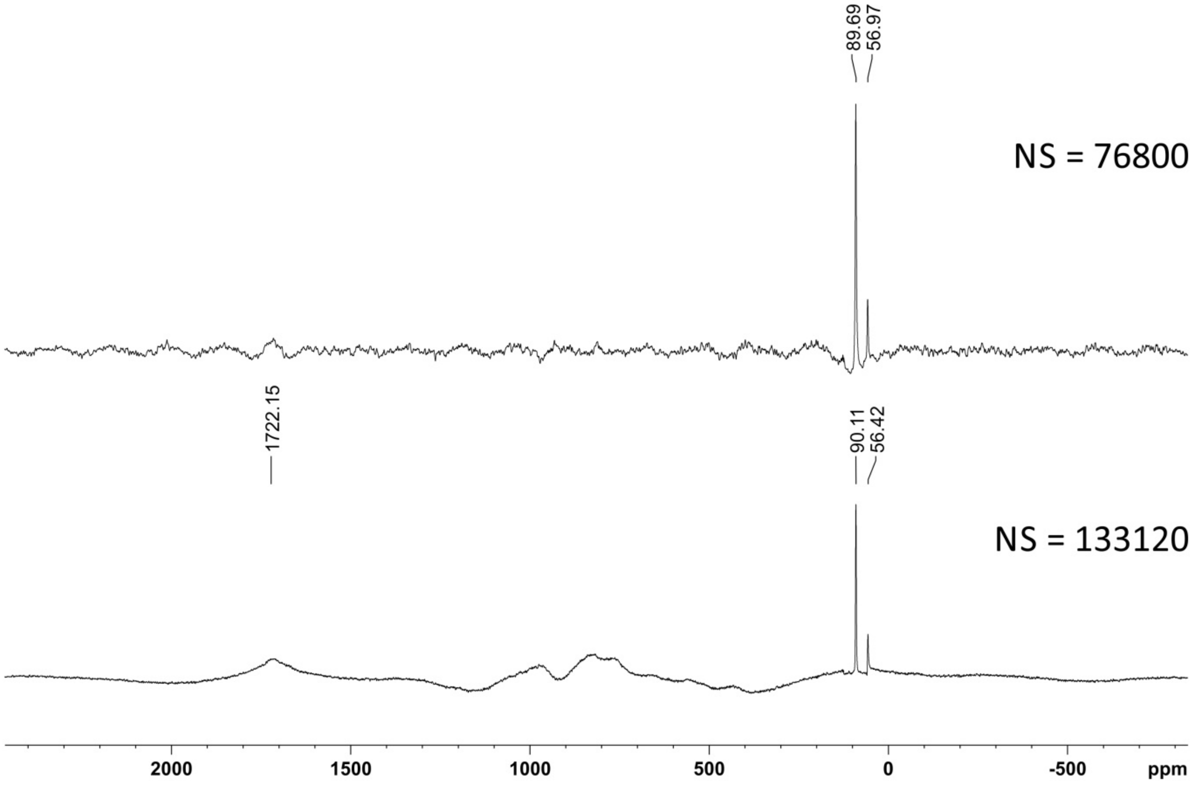

Figure S12. **^{125}Te NMR** spectrum of a mixture of **2a** and **2b** (126.24 MHz, THF-d_8, 294 K) measured at two different number of scans (NS).

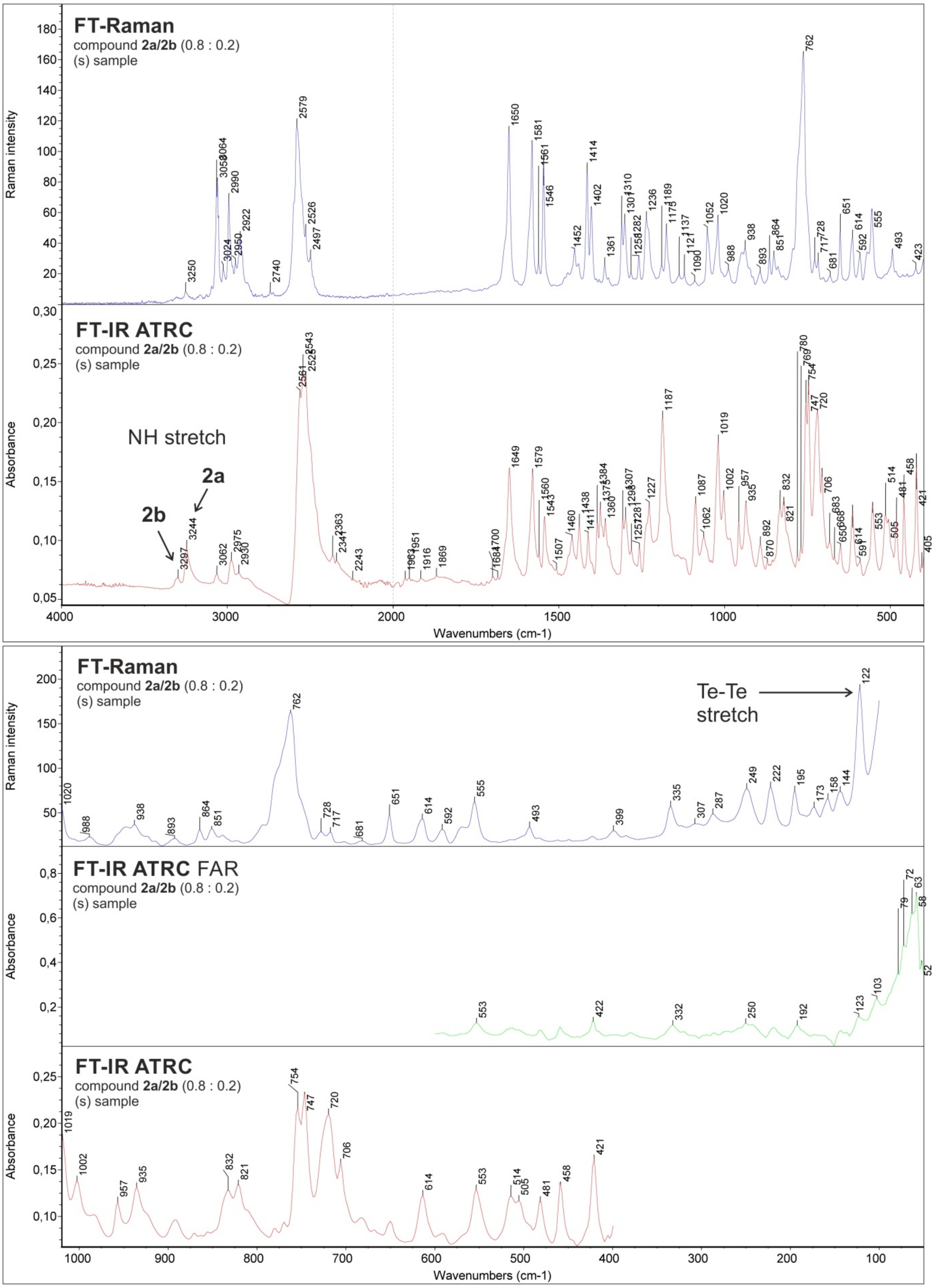

Figure S13. FT-IR ATRC spectra and FT-Raman emission spectra of compound **2a/2b**.

NMR and vibration spectra-based evidences proving dissociation of Te→Te^{+} bond of 2a/2b upon dissolving in THF

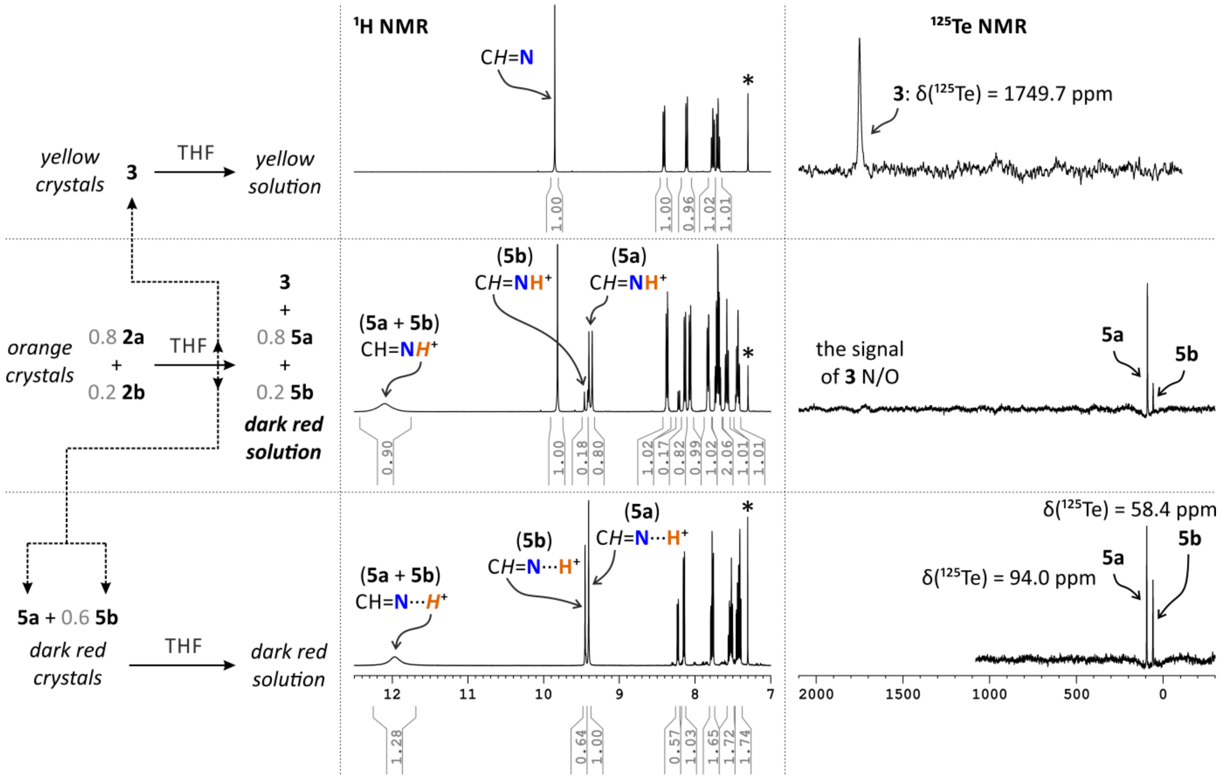

Figure S14. Stacked plot of cut-outs of ^{1}H NMR spectra (500.20 MHz, 294 K) in the region 7.0 – 12.5 ppm and of corresponding ^{125}Te NMR spectra (126.24 MHz, 294 K) in the region -300 – 2100 ppm proving dissociation of the Te→Te^{+} bond of compounds **2a**/**2b** when dissolved in THF-d_8. * trace amount of benzene. N/O = not observed.

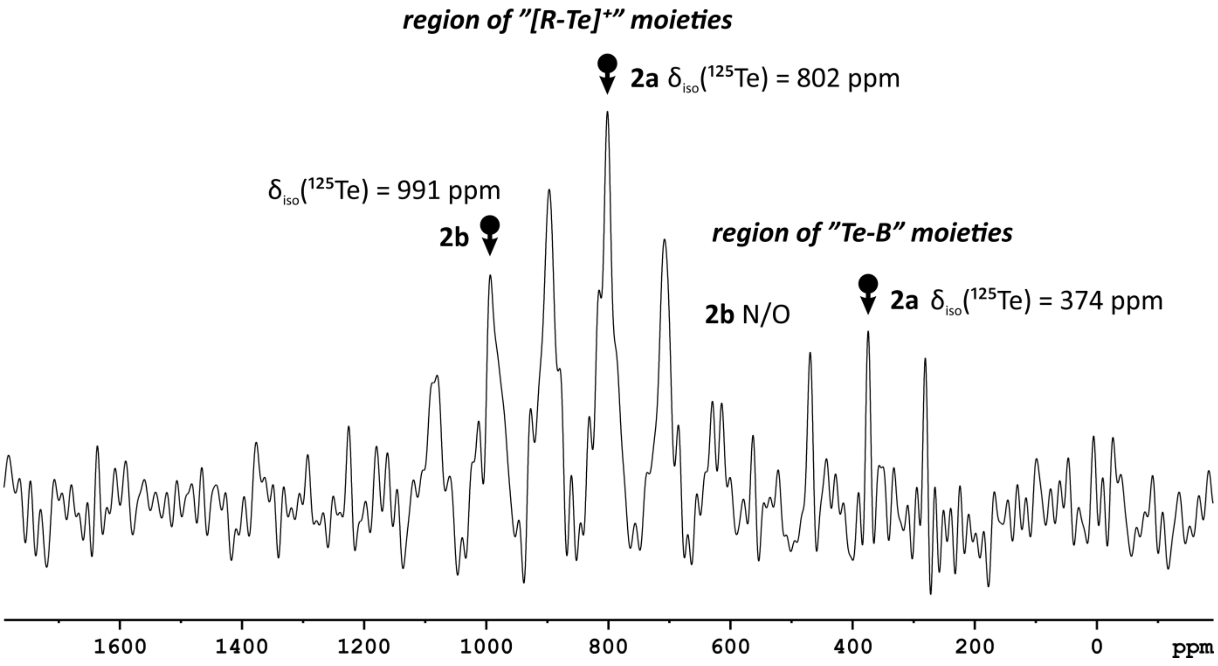

Figure S15. Hahn Echo ^{125}Te NMR MAS spectrum (157.81 MHz, MAS rate = 15 kHz, NS = 5717) of powder with composition 0.8 **2a** + 0.2 **2b**. The isotropic chemical shifts δ_{iso} were determined by comparison of two acquisitions measured at MAS rate 15 and 20 kHz. The fact that mutual ratio between the RTe^{+} unit and the “Te-B” unit within one isomer of compound **2** is not 1:1 is probably caused by their different relaxation time in the MAS NMR experiment. To rule out the sample decomposition within the MAS NMR experiment, the content of 3.2 mm zirconia rotor was after all analyzed by ^{1}H NMR in [D_8]THF. This analysis proved stability (identity) of given sample within MAS NMR experiment as only signals of mixture of compounds **5a/5b** next to signals of compounds **3** were observed.

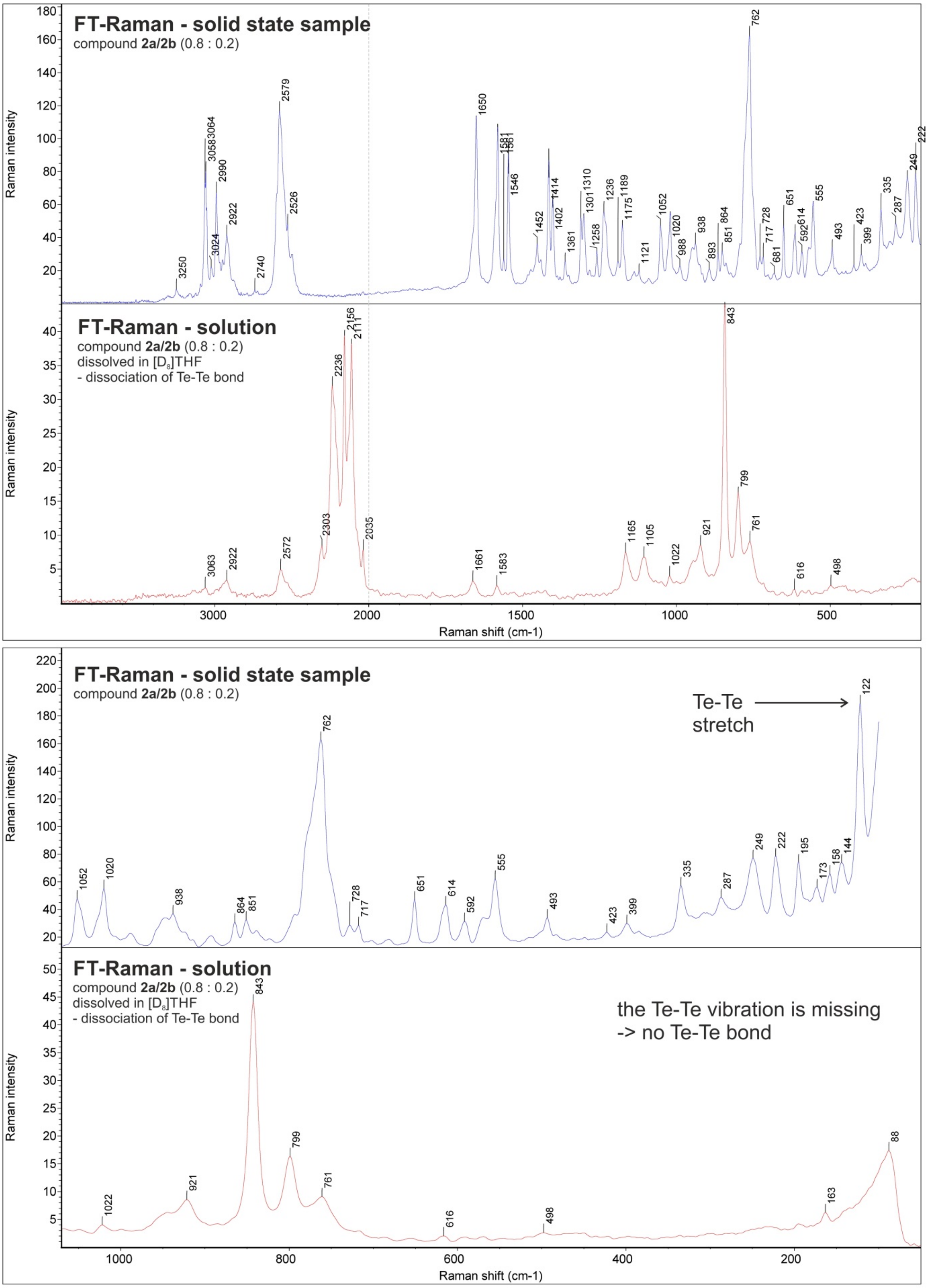

Figure S16. FT-Raman emission spectra of compound **2a/2b** in (s) and as a [D_8]THF solution.

Synthesis of [2-(*t*BuNCH)C_6H_4Te·THF][$CB_{11}H_{12}$] (3).

2-(*t*BuNCH)C_6H_4TeCl (**II**) (524 mg, 1.62 mmol) and $Ag^+CB_{11}H_{12}^-$ (407 mg, 1.62 mmol) were loaded into a Schlenk tube under argon atmosphere, THF was added (15 mL) and the reaction mixture was stirred for 15 min at RT with exclusion of light. The resulting ivory suspension was filtered and the volume of the obtained yellowish filtrate was reduced to approx. 2 mL without any heating. This highly concentrated THF solution was layered with hexane (2 mL) and after storing over one day at 6 °C huge yellowish single-crystals of compound **3** were obtained. Isolated yield 774 mg (95 %); m.p. 119 °C (decomp.).

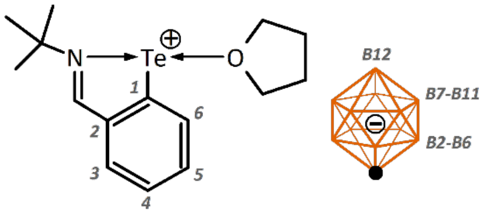

^{1}H NMR (400.13 MHz, THF-d_8) δ (ppm): 1.77 [9H, s, (C*H*$_3$)$_3$C-]; 0.93–2.27 [11H, m, $HCB_{11}H_{11}^-$]; 2.12 [1H, s, *H1*-$CB_{11}H_{11}^-$]; 7.69 [1H, dt, Ar(C4)-*H*]; 7.76 [1H, dt, Ar(C5)-*H*]; 8.11 [1H, d, Ar(C6)-*H*]; 8.41 [1H, dd, Ar(C3)-*H*]; 9.85 [1H, s, C*H*=N]. **^{11}B NMR** (160.48 MHz, THF-d_8) δ (ppm): -16.8 [5B, d, *B2–B6*), $^1J(^{11}B,^1H)$ = 149.5 Hz]; –13.7 [5B, d, *B7–B11*), $^1J(^{11}B,^1H)$ = 136.9 Hz]; –7.3 [1B, d, *B12*, $^1J(^{11}B,^1H)$ = 134.7 Hz]. **^{13}C{^{1}H} NMR** (100.61 MHz, THF-d_8) δ (ppm): 32.3 [s, (*C*H$_3$)$_3$C-]; 51.6 [s, *C*$B_{11}H_{12}^-$]; 66.4 [s, (CH$_3$)$_3$*C*-]; 129.0 [s, Ar-*C4*]; 132.1 [s, Ar-*C6*]; 134.1 [s, Ar-*C5*]; 135.0 [s, Ar-*C3*]; 138.9 [s, qC, Ar-*C2*]; 143.4 [br. s, qC, Ar-*C1*(*ipso*)]; 167.3 [s, *C*H=N]. **^{15}N NMR** (50.70 MHz, THF-d_8) δ: –132.2 ppm. **^{125}Te NMR** (126.24 MHz, THF-d_8) δ: 1749.7 ppm.

Compound **3** is highly soluble in THF, but virtually insoluble in aliphatic solvents.

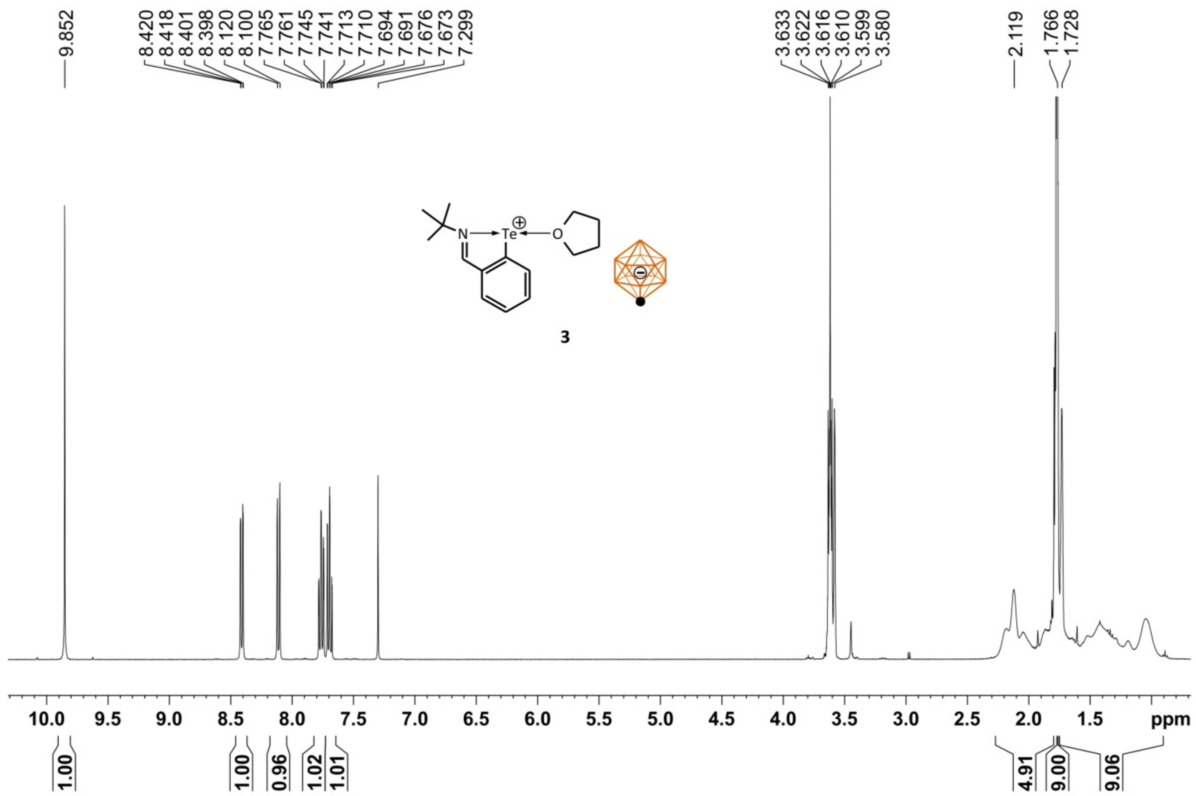

Figure S17. **^{1}H NMR** spectrum of **3** (400.13 MHz, THF-d_8, 294 K).

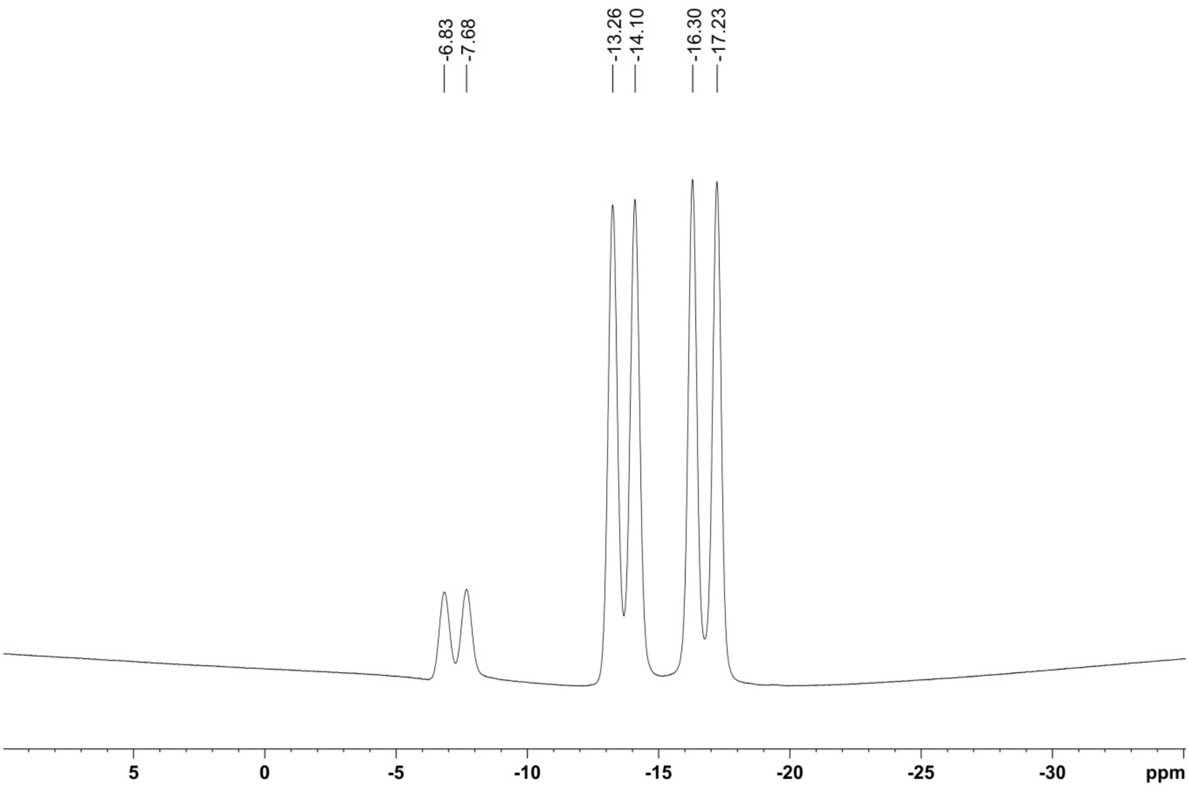

Figure S18. **^{11}B NMR** spectrum of **3** (160.48 MHz, THF-d_8, 294 K).

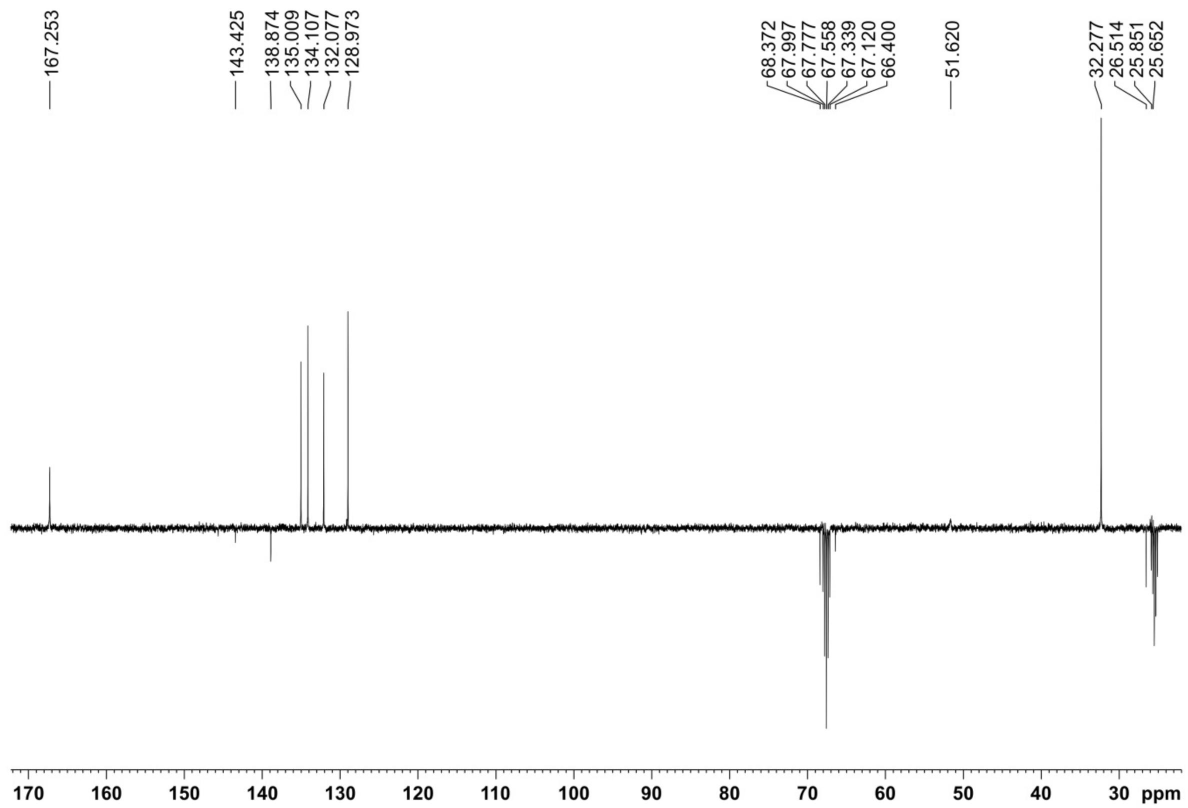

Figure S19. ^{13}**C{**^{1}**H} APT NMR** spectrum of **3** (100.61 MHz, THF-d_8, 294 K).

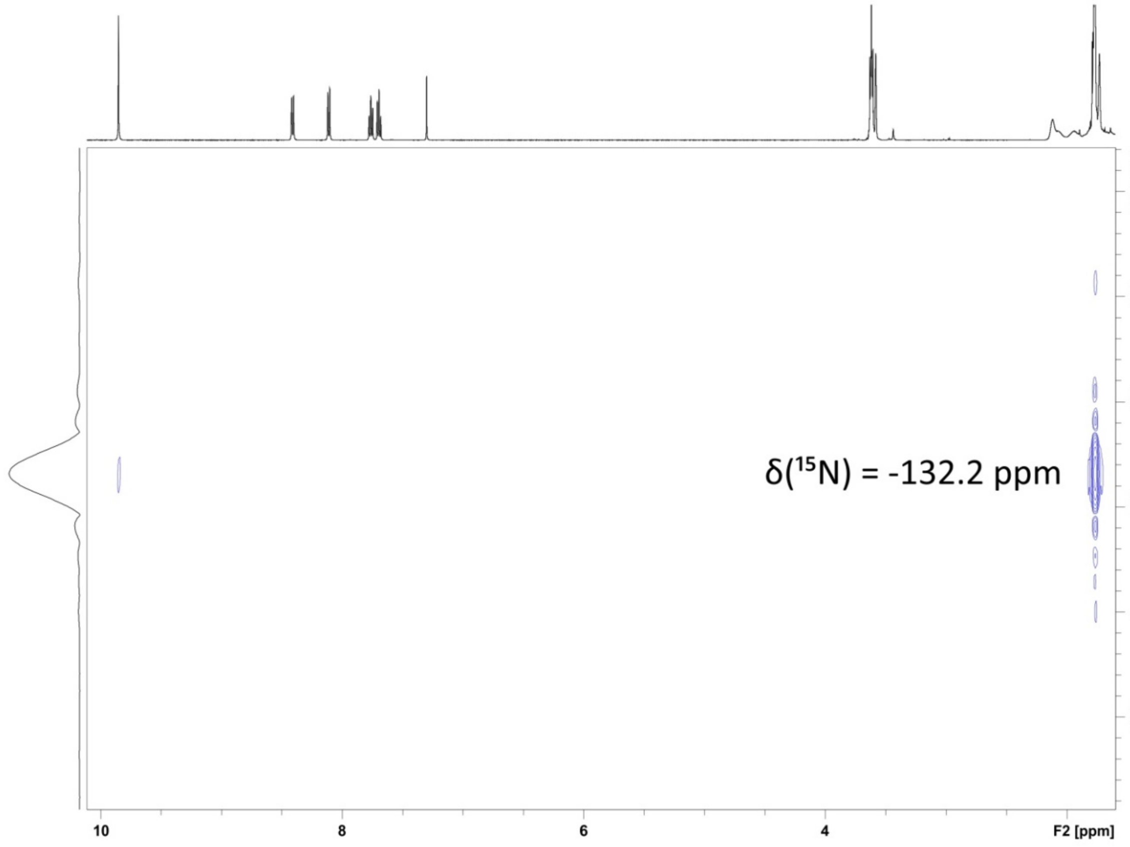

Figure S20. ^{1}**H-**^{15}**N HMBC** NMR spectrum of **3** (400.13 MHz, THF-d_8, cnst13 = 5 Hz, 294 K).

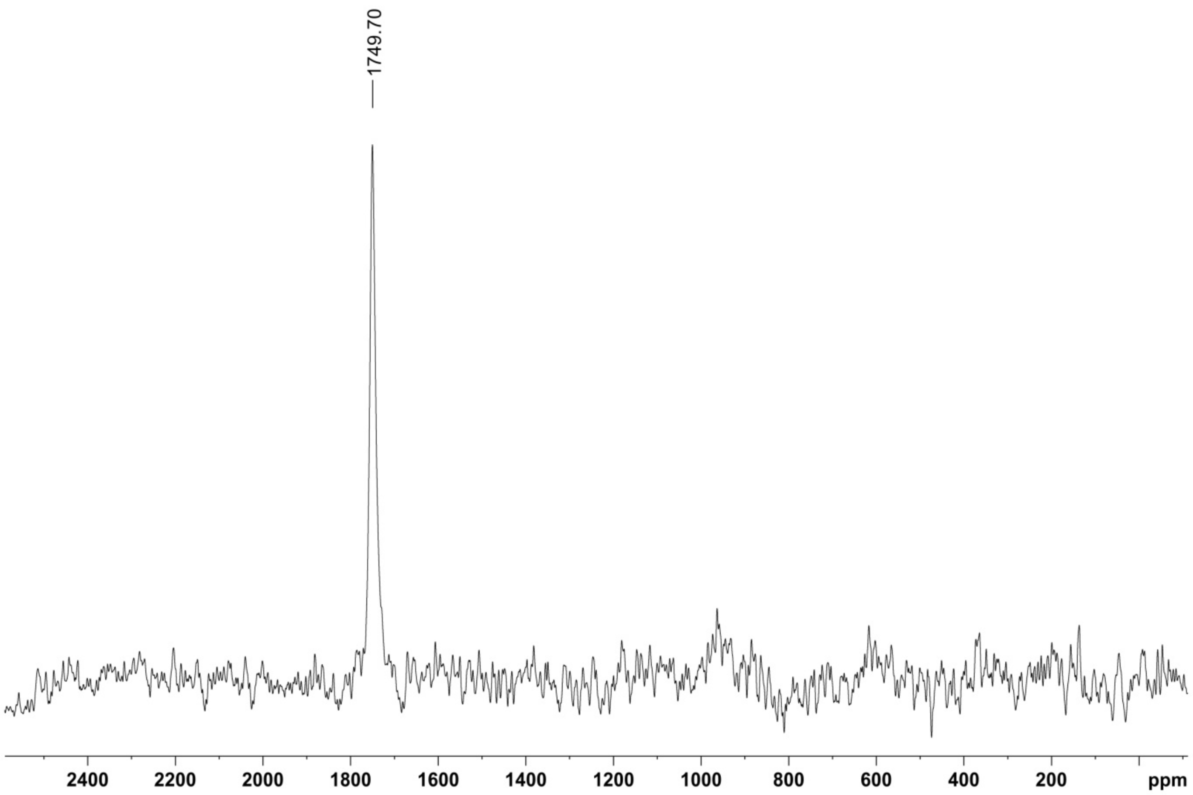

Figure S21. **^{125}Te NMR** spectrum of **3** (126.24 MHz, THF-d_8, 294 K, NS = 102400).

Synthesis of [2-(*t*BuNCH)C_6H_4Te·DMAP][$CB_{11}H_{12}$] (4).

Option A) 2-(*t*BuNCH)C_6H_4TeCl (**II**) (284 mg, 0.88 mmol), $Ag^+CB_{11}H_{12}^-$ (220 mg, 0.88 mmol) and 4-*N,N*-dimethylaminopyridine (DMAP; 107 mg, 0.88 mmol) were loaded into a Schlenk tube under argon atmosphere, CH_2Cl_2 was added (15 mL) and the reaction mixture was stirred for 15 min at RT with exclusion of light. The resulting ivory suspension was filtered and the volume of the obtained yellowish filtrate was reduced to approx. 2 mL. This highly concentrated CH_2Cl_2 solution was layered with hexane (2 mL) and after storing over one day at 6 °C huge yellowish single-crystals of compound **4** were obtained. Isolated yield 402 mg (83 %); m.p. 203 °C (decomp.).

Option B) [2-(tBuNCH)C_6H_4Te·THF][$CB_{11}H_{12}$] (**3**) (71 mg, 0.14 mmol) and 4-*N,N*-dimethylaminopyridine (DMAP; 17 mg, 0.14 mmol) were loaded into NMR tube under argon atmosphere and dissolved in 0.5 mL of THF-d_8. According to NMR spectroscopy the yield of the reaction is quantitative.

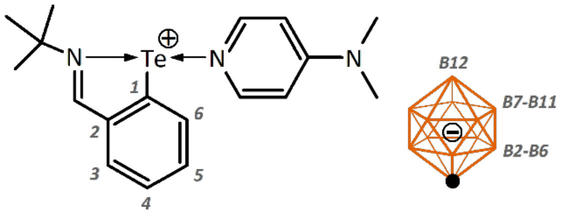

^{1}H NMR (500.20 MHz, THF-d_8) δ (ppm): 1.70 [9H, s, (CH_3)$_3$C-]; 1.03–2.15 [11H, m, H$CB_{11}H_{11}^-$]; 2.10 [1H, s, *H1*-$CB_{11}H_{11}^-$]; 3.20 [6H, s, (DMAP)-N(CH_3)$_2$]; 6.86 [2H, d, (DMAP)-β-C*H*]; 6.99 [1H, br. d, Ar(C6)-*H*]; 7.46 [1H, t, Ar(C5)-*H*]; 7.55 [1H, t, Ar(C4)-*H*]; 8.21 [1H, d, Ar(C3)-*H*]; 8.32 [2H, br. d, (DMAP)-α-C*H*]; 9.59 [1H, s, Ar-C*H*=N-tBu]. **^{11}B NMR** (160.48 MHz, THF-d_8) δ (ppm): -16.7 [5B, d, *B2–B6*), $^1J(^{11}B,^1H)$ = 149.2 Hz]; -13.5 [5B, d, *B7–B11*), $^1J(^{11}B,^1H)$ = 136.4 Hz]; –7.1 [1B, d, *B12*, $^1J(^{11}B,^1H)$ = 136.1 Hz]. **^{13}C{^{1}H} NMR** (125.78 MHz, THF-d_8) δ (ppm): 32.1 [s, (CH_3)$_3$C-]; 39.9 [2C, s, (DMAP)-N(CH_3)$_2$]; 51.6 [s, $CB_{11}H_{12}^-$]; 63.6 [s, (CH_3)$_3$*C*-]; 109.7 [2C, s, (DMAP)-β-*C*H]; 128.3 [s, Ar-*C4*]; 131.7 [s, Ar-*C6*]; 133.2 [s, Ar-*C5*]; 134.7 [s, Ar-*C3*]; 137.4 [s, qC, Ar-*C2*]; 137.8 [br. s, qC, Ar-*C1*(*ipso*)]; 150.0 [2C, s, (DMAP)-α-*C*H]; 157.1 [s, qC, (DMAP)-γ-*C*]; 164.4 [s, Ar-*C*H=N-tBu]. **^{15}N NMR** (50.70 MHz, THF-d_8) δ (ppm): –306.5 [s, (DMAP)-*N*(CH_3)$_2$]; –187.8 [s, (DMAP)-*N1*]; –100.5 [s, Ar-CH=*N*-tBu]. **^{125}Te NMR** (126.24 MHz, THF-d_8) δ: 1446.0 ppm. Compound **4** is highly soluble in THF, but virtually insoluble in aliphatic and aromatic solvents.

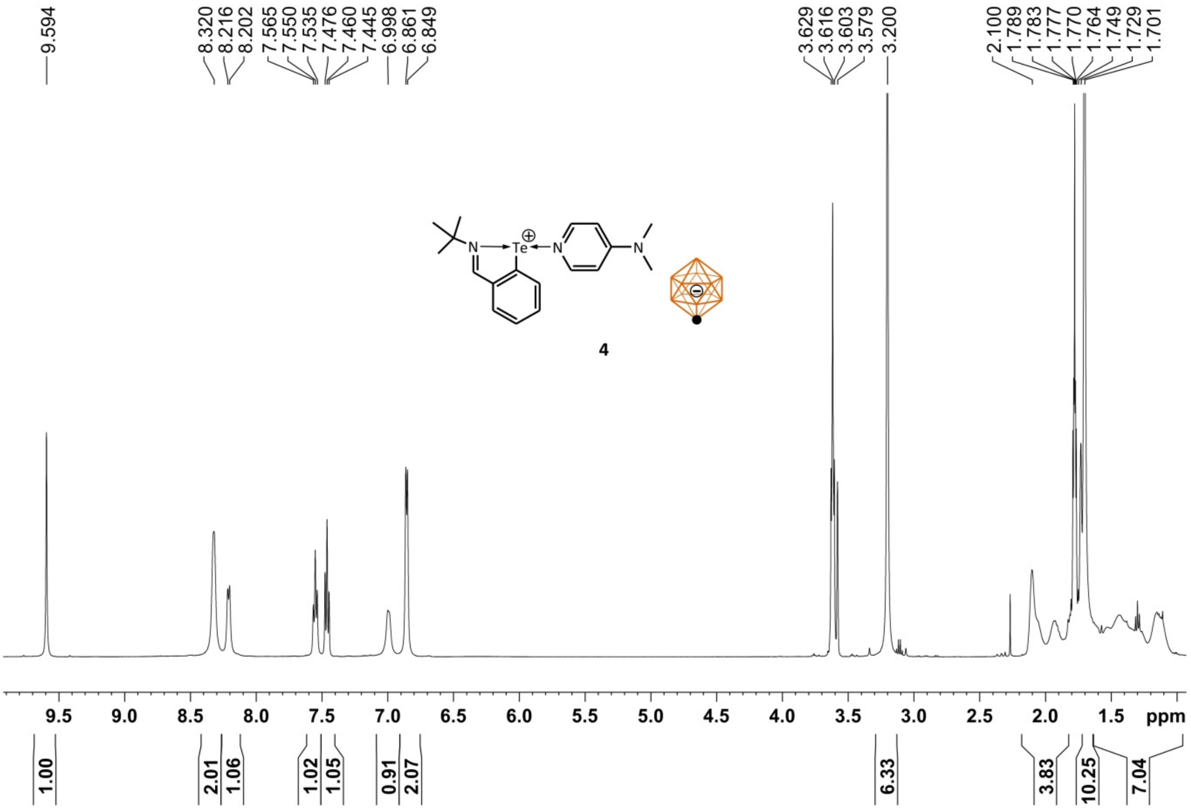

Figure S22. **^{1}H NMR** spectrum of **4** (500.20 MHz, THF-d_8, 294 K).

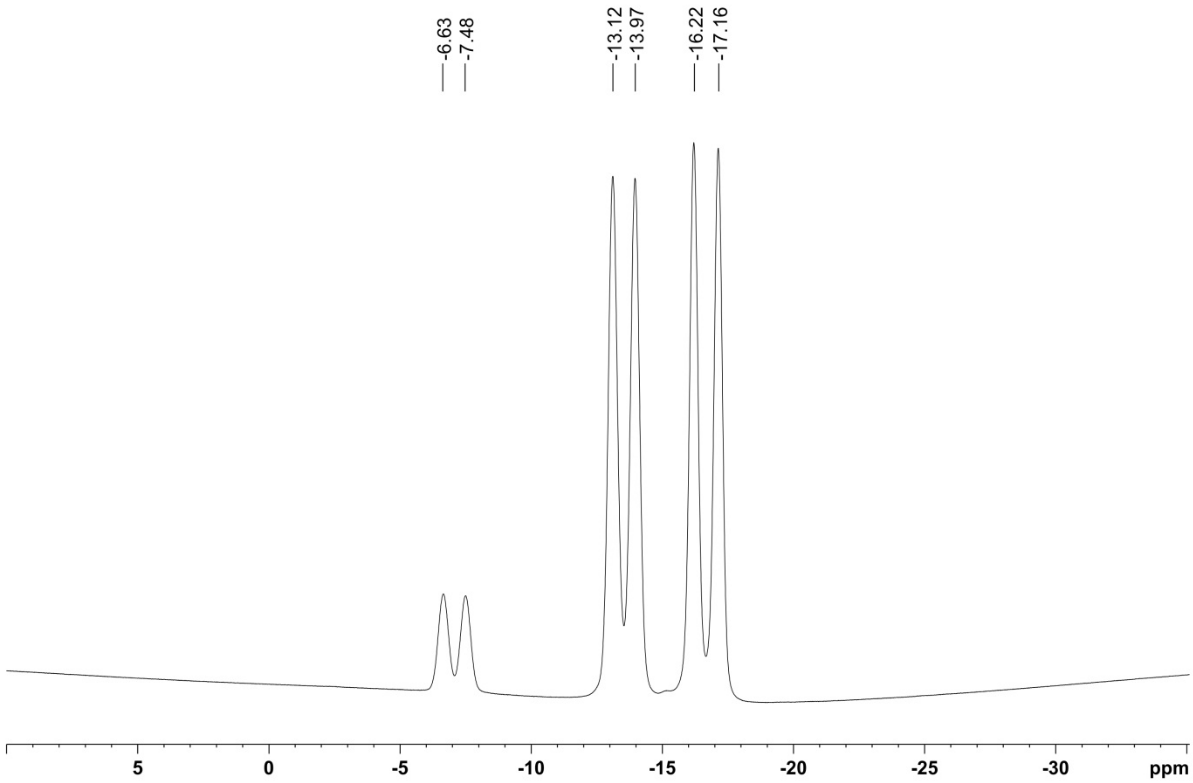

Figure S23. **^{11}B NMR** spectrum of **4** (160.48 MHz, THF-d_8, 294 K).

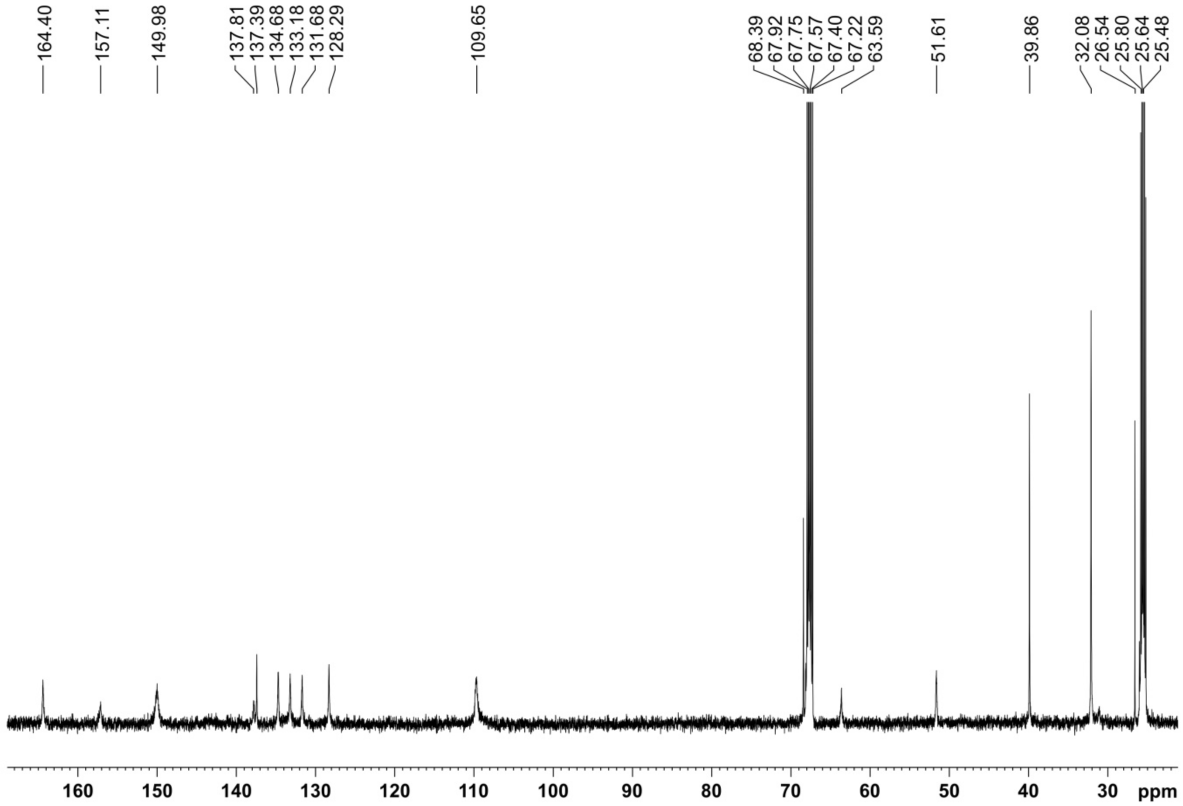

Figure S24. $^{13}C\{^1H\}$ **NMR** spectrum of **4** (125.78 MHz, THF-d_8, 294 K).

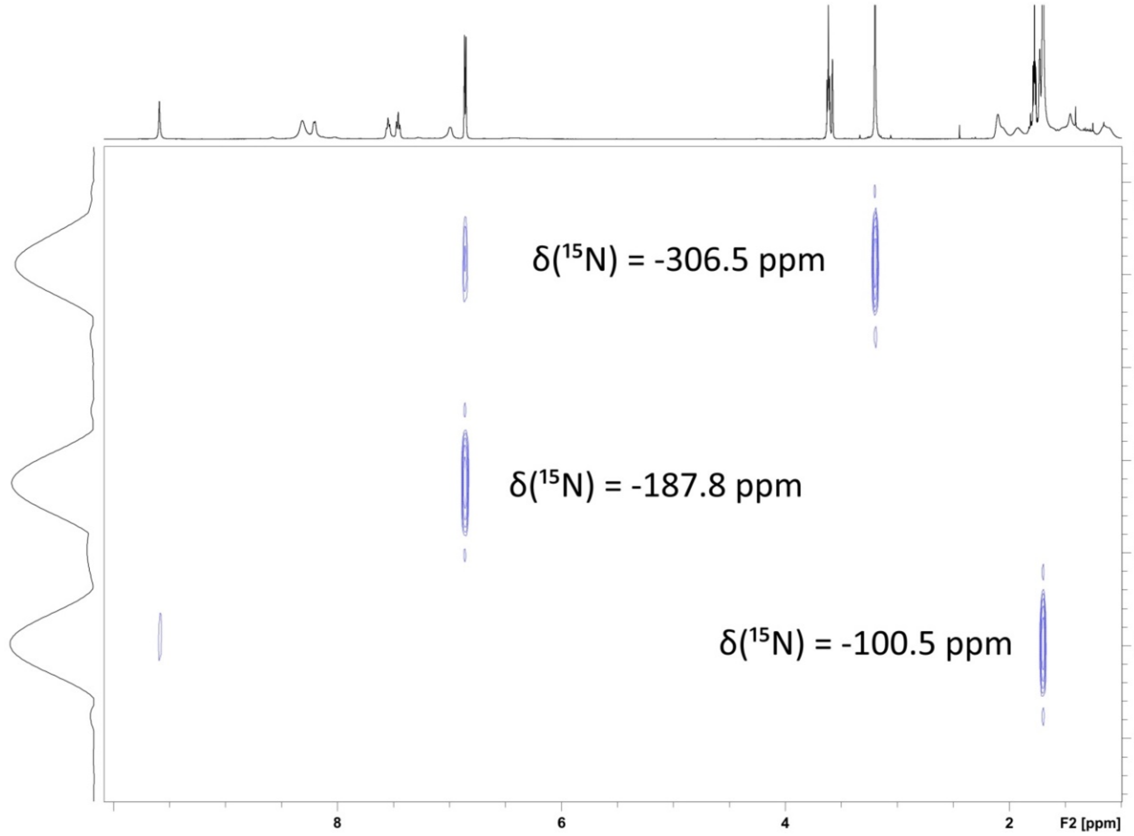

Figure S25. ^1H-^{15}N **HMBC** NMR spectrum of **4** (500.20 MHz, THF-d_8, cnst13 = 5 Hz, 294 K).

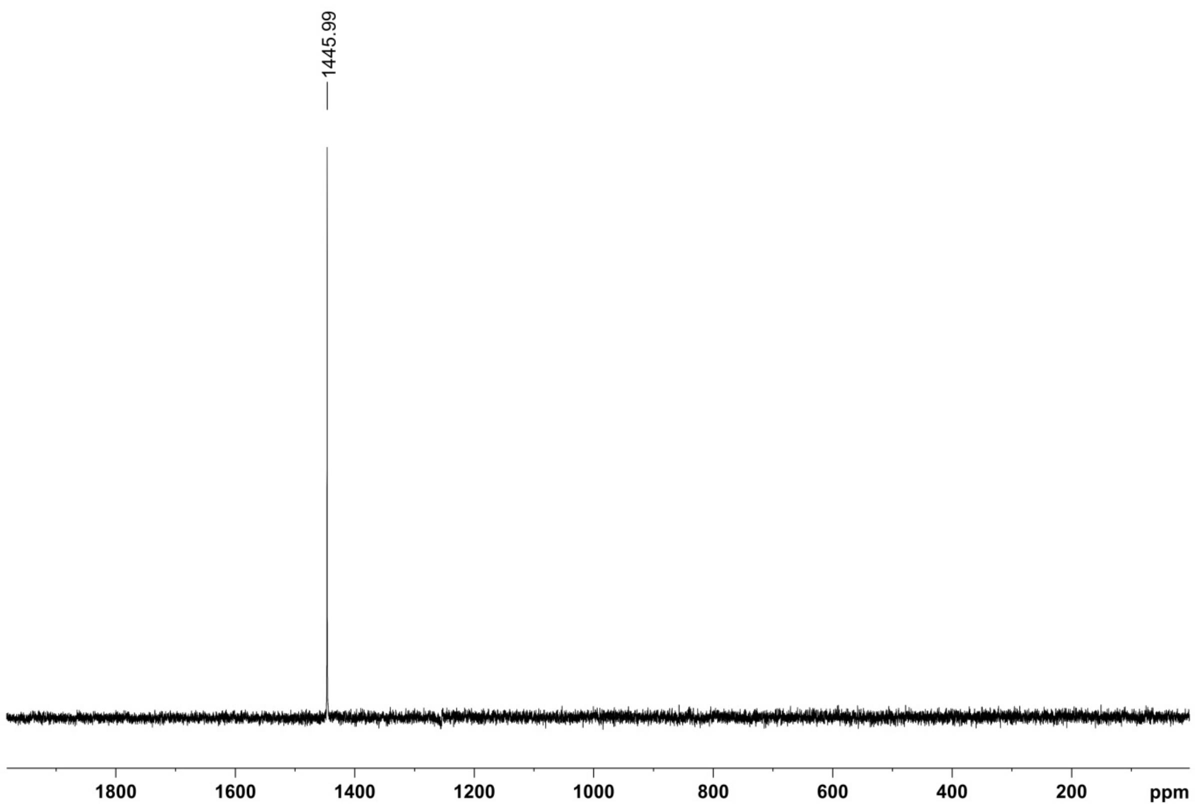

Figure S26. **^{125}Te NMR** spectrum of **4** (126.24 MHz, THF-d_8, 294 K, NS = 3072).

Synthesis of 12-[2-(*t*BuN{H}CH)C_6H_4Te]$CB_{11}H_{11}$ (5a) and 7-[2-(*t*BuN{H}CH)C_6H_4Te]$CB_{11}H_{11}$ (5b):

Option A) Crystals of [2-(*t*BuNCH)C_6H_4Te·THF][$CB_{11}H_{12}$] (**3**) (576 mg, 1.15 mmol) were loaded into a previously annealed glass ampoule (10 mm outside diameter) under argon atmosphere. The part of the ampoule containing the compound was heated by a heat gun (with maximal output 2300 W) set to 140 °C, while the ampoule was still under argon atmosphere. The material inside the ampoule immediately started to change color from yellowish to red. After 2 hours of heating, while the material melts in the beginning and then the re-solidification of dark red material occurs later on, the ampoule was cautiously evacuated during constant heating for next half an hour to obtain full conversion into product and to remove traces of THF – two regioisomers **5a** and **5b** with a larger representation of **5a** (12-regioisomer) [mutual ratio **5a** : **5b** = 0.62 : 0.38]. Compounds **5a** and **5b** were obtained as a

dark red polycrystalline material in isolated yield 490 mg (99 %). Suitable single-crystals were grown from a minimal amount of boiling toluene [with composition (**5a**/**5b**)·**toluene**; m.p. 217 °C] and from minimal amount of hot $C_2D_2Cl_4$ [with composition (**5a**/**5b**)·$\mathbf{C_2D_2Cl_4}$].

Option B) The yellowish solution of [2-(*t*BuNCH)C_6H_4Te·THF][$CB_{11}H_{12}$] (**3**) (178 mg, 0.35 mmol) in THF (15 mL) was loaded into a previously annealed heavy walled tall Schlenk bomb closable by the means of bore plug Young valve (PTFE). The Schlenk bomb was pressurized by argon and the solution was heated in an oil bath at 77 °C for 12 hours to obtain full conversion into products – two regioisomers **5a** and **5b** with a larger representation of **5a** (12-regioisomer) [mutual ratio was in this case **5a** : **5b** = 1 : 0.31]. After that time, the volume of the obtained blood-red solution was evaporated at low pressure to give compounds **5a** and **5b** as a dark red polycrystalline material. Isolated yield 151 mg (99 %).

Note: Mixture of compounds **5a/5b** dissolved in [D8]THF shows significant thermochromic behavior. The higher the temperature, the far more intense is the blood-red color of the solution. While at –40 °C the NMR sample is only light red colored which allows to sample to be transparent, at 100 °C (at elevated pressure) is dark red colored and cannot be seen through.

Kinetics of transformation of compound 3 into compounds 5a and 5b

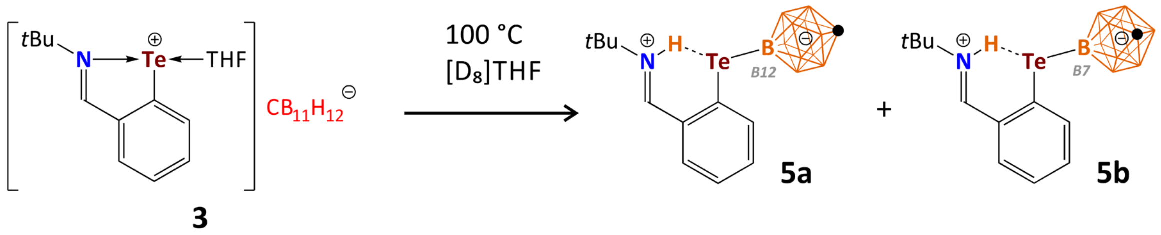

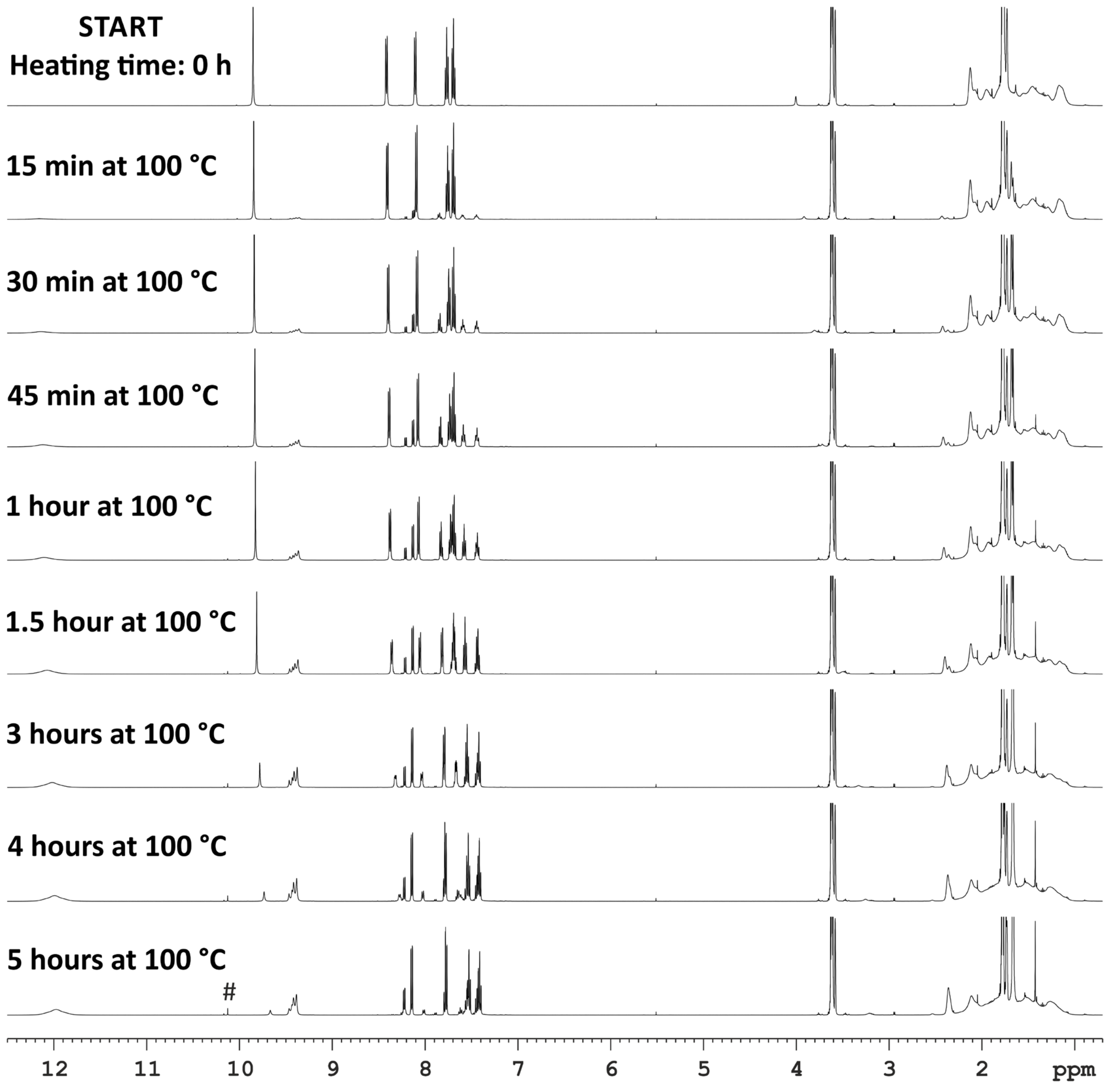

Figure S27. Stacked plot of ^{1}H NMR spectra (500.20 MHz, 294 K) showing transformation of the starting compound **3** via Option B (see description above) into a mixture of compounds **5a** and **5b** when heated in THF-d_8. # - minority of decomposition products.

Table S2. Experimentally obtained relative concentration of compound **3** vs **5a+5b** obtained by integration in ^{1}H NMR spectra, specifically by integration of intensity of C*H*=NH^{+} doublets of both **5a** and **5b** and CH=N singlet of compound **3**.

experimental data obtained at 85.4 °C				experimental data obtained at 100.0 °C				experimental data obtained at 115.5 °C			
time [hours]	time [s]	rel. [**3**]	ln(rel. [**3**])	time [hours]	time [s]	rel. [**3**]	ln(rel. [**3**])	time [hours]	time [s]	rel. [**3**]	ln(rel. [**3**])
0	0	1	0	0	0	1	0	0	0	1	0
0.25	900	0.9867	-0.0134	0,25	900	0.9252	-0.0777	5	300	0.7778	-0.2513
0,5	1800	0.9520	-0.0492	0,5	1800	0.8029	-0.2195	10	600	0.6045	-0.5033
0.75	2700	0.9252	-0.0778	0,75	2700	0.6993	-0.3577	15	900	0.4811	-0.7317
1	3600	0.8979	-0.1077	1	3600	0.6053	-0.5020	20	1200	0.3948	-0.9294
1.5	5400	0.8368	-0.1782	1,5	5400	0.4502	-0.7980	25	1500	0.3373	-1.0868
2	7200	0.7846	-0.2426	3	10800	0.2048	-1.5857				
2.5	9000	0.7351	-0.3077	4	14400	0.1263	-2.0688				
3	10800	0.6897	-0.3715	5	18000	0.0785	-2.5449				
4	14400	0.6078	-0.4979								
5	18000	0.5452	-0.6067								
6	21600	0.4900	-0.7134								

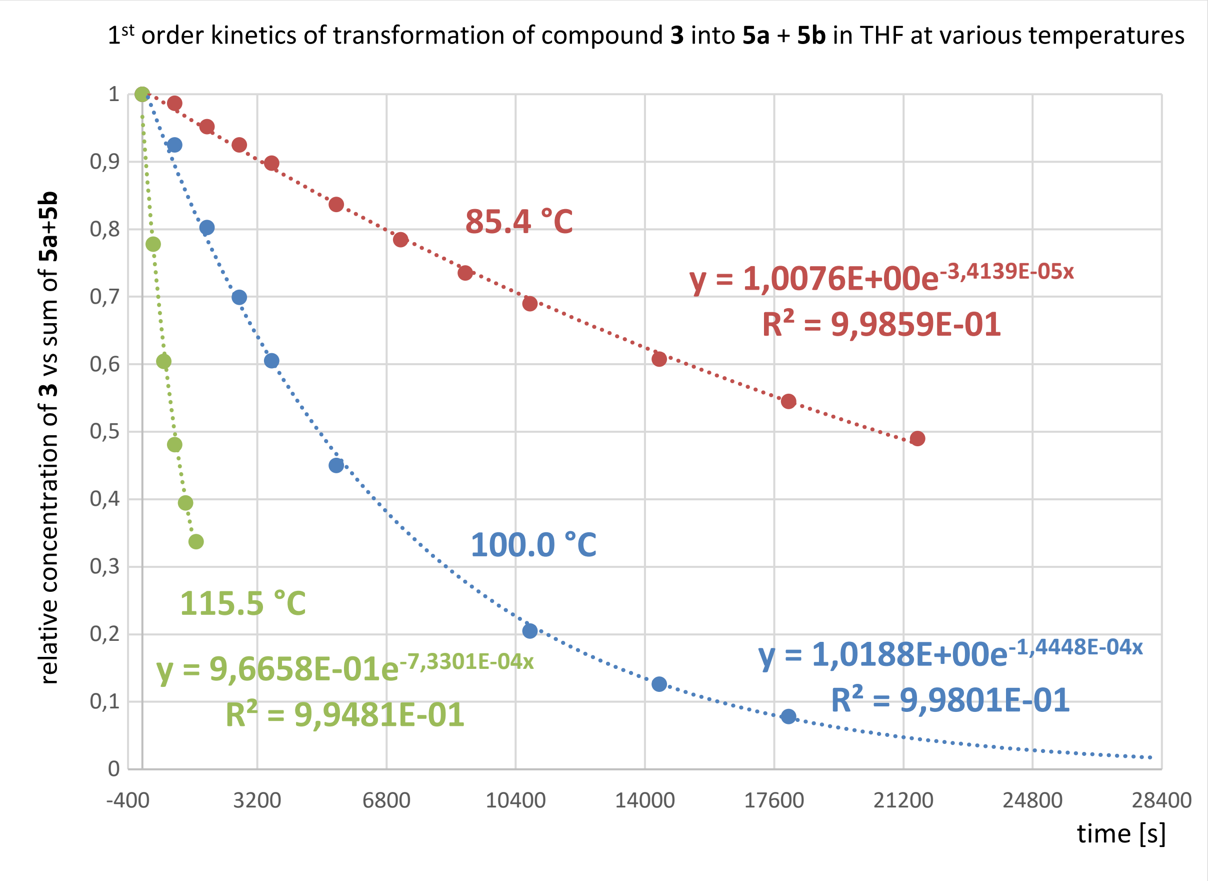

Figure S28. Plots of experimental data obtained for heating of **3** in [D$_8$]THF (measured values can be found in Table S2 above) for determination of rate constants *k*.

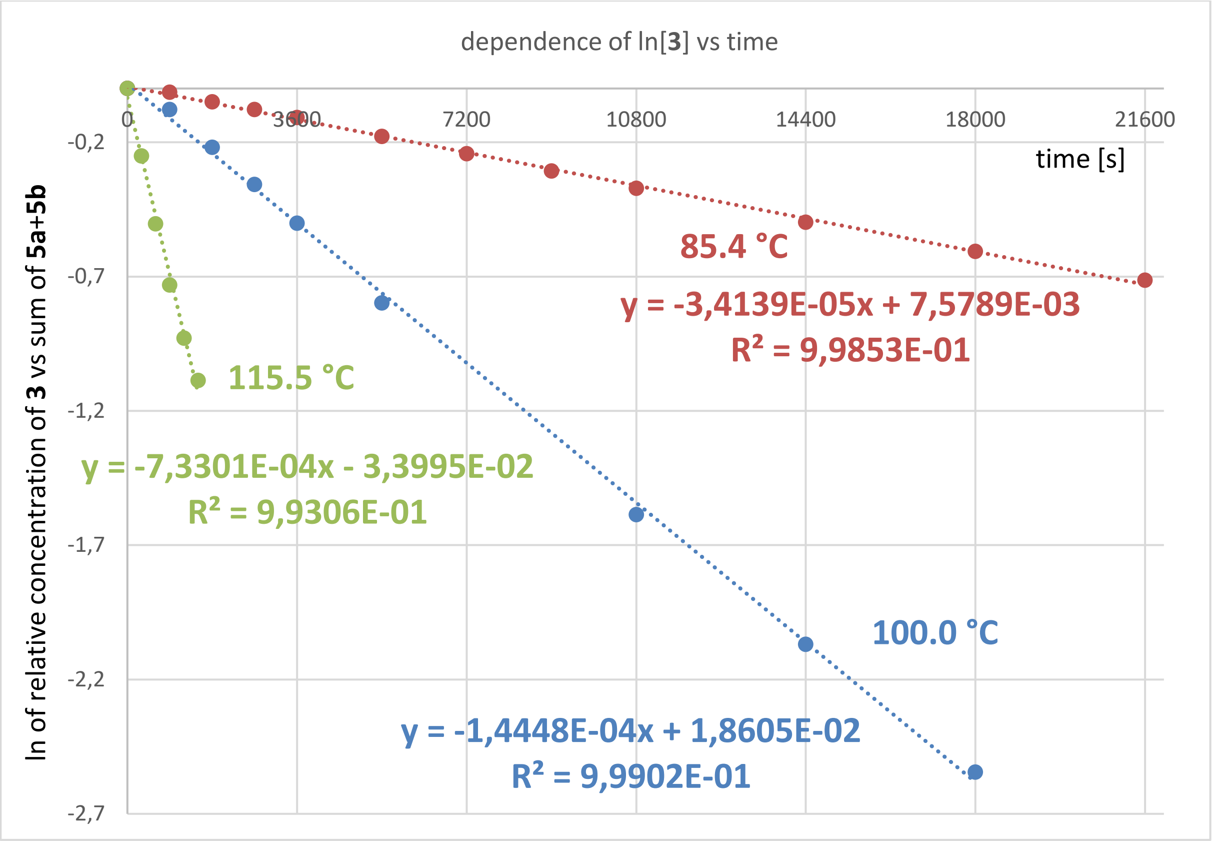

Continuation of Figure S28.

Based on Figure S28, reaction of **3** in THF to **5a/5b** proceeds as 1st order kinetics.

$k^{85.4°C}$ = 0.0000341 s^{-1} (half-life at 85.4 °C is 5.64 hours)

$k^{100°C}$ = 0.0001444 s^{-1} (half-life at 100 °C is 1.33 hours)

$k^{115.5°C}$ = 0.0007330 s^{-1} (half-life at 115.5 °C is 0.26 hour)

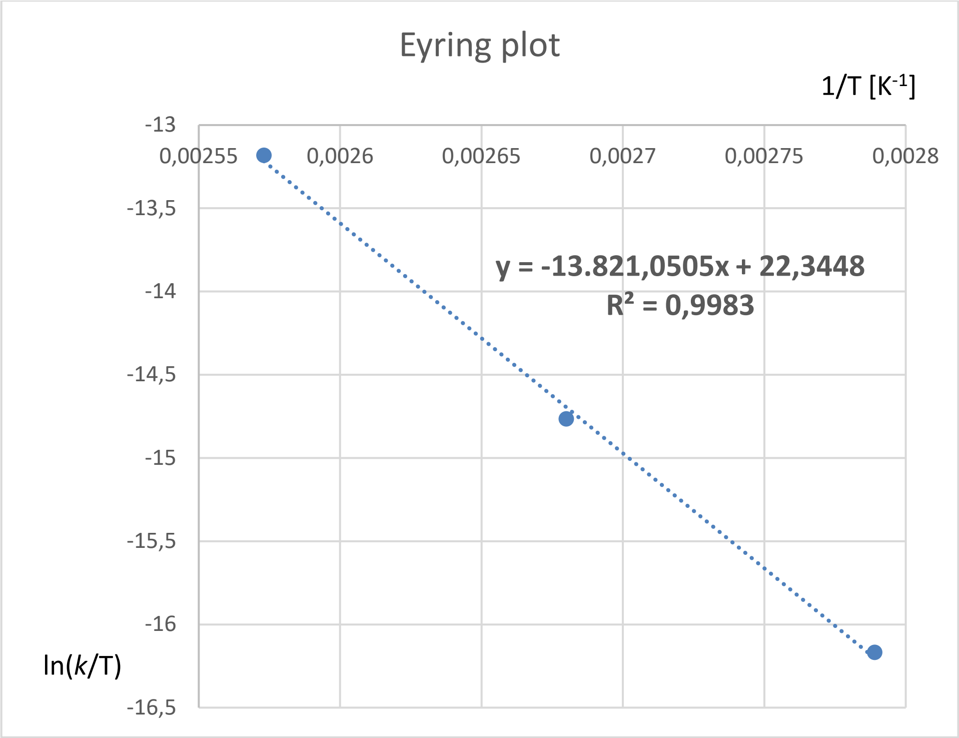

Figure S29. Eyring plot for data summarized above **-** heating of compound **3** in [D_8]THF.

intercept = *22.3448*

slope = *–13821.0505*

intercept = $\Delta S^‡/R$ + 23.76

slope = $\Delta H^‡/–R$

$\Delta S^‡$ = –11.8 J mol^{-1} K^{-1}

$\Delta H^‡$ = 114.9 kJ mol^{-1}

$\Delta G^‡_{298}$ = 118.4 kJ mol^{-1}

NMR data for **12-[2-(*t*BuN{H}CH)C_6H_4Te]$CB_{11}H_{11}$ (5a)** (major isomer) in THF-d_8:

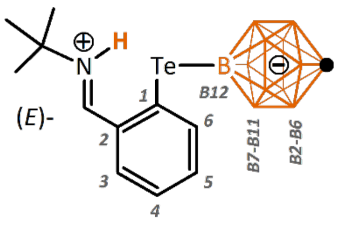

^{1}H NMR (500.20 MHz, THF-d_8) δ (ppm): 1.67 [9H, s, (CH_3)$_3$C-]; 1.06–2.27 [11H, m, HCB$_{11}$$H_{11}^{-}$]; 2.35 [1H, s, *H1*-CB$_{11}H_{11}^{-}$]; 7.41 [1H, td, Ar(C5)-*H*]; 7.52 [1H, td, Ar(C4)-*H*]; 7.76 [1H, dd, Ar(C3)-*H*]; 8.14 [1H, dd, Ar(C6)-*H*]; 9.40 [1H, s, Ar-C*H*=NH$^+$-*t*Bu, $\Delta\nu^1/_2$ = 2.4 Hz]; 11.97 [1H, vbr. s, Ar-CH=N*H*$^+$-*t*Bu, $\Delta\nu^1/_2$ = 80 Hz]. **^{11}B NMR** (160.48 MHz, THF-d_8) δ (ppm): –15.8 [5B, d, *B2–B6*, $^{1}J(^{11}$B,^{1}H) = N/A]; –12.5 [5B, d, *B7–B11*, $^{1}J(^{11}$B,^{1}H) = N/A]; –11.5 [1B, s, *B12* (i.e. Te-*B*)]. **^{13}C{^{1}H} NMR** (100.61 MHz, THF-d_8) δ (ppm): 28.0 [s, (*C*H$_3$)$_3$C-]; 51.8 [s, *C*B$_{11}$H$_{12}^{-}$]; 62.5 [s, (CH$_3$)$_3$*C*-]; 122.7 [s, qC, Ar-*C1*(*ipso*)]; 129.2 [s, Ar-*C4*]; 129.5 [s, Ar-*C3*]; 134.6 [s, qC, Ar-*C2*]; 135.9 [s, Ar-*C5*]; 146.2 [s, Ar-*C6*]; 174.6 [s, Ar-*C*H=NH$^+$-*t*Bu]. **^{15}N NMR** (40.54 MHz, THF-d_8) δ (ppm): –172.1 ppm. **^{125}Te NMR** (126.24 MHz, THF-d_8) δ: 94.0 ppm.

Solid state sample: **IR** (diamond ATR): 3244 cm^{-1} (NH stretch).

NMR data for **7-[2-(*t*BuN{H}CH)C_6H_4Te]$CB_{11}H_{11}$** (**5b**) (minor isomer) in THF-d_8:

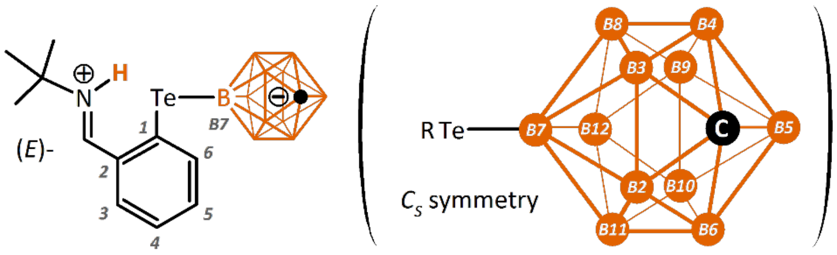

^{1}H NMR (500.20 MHz, THF-d_8) δ (ppm): 1.66 [9H, s, (CH_3)$_3$C-]; 1.06–2.27 [11H, m, HCB$_{11}$$H_{11}^{-}$]; 2.35 [1H, s, *H1*-CB$_{11}H_{11}^{-}$]; 7.44 [1H, td, Ar(C5)-*H*]; 7.54 [1H, td, Ar(C4)-*H*]; 7.78 [1H, dd, Ar(C3)-*H*]; 8.23 [1H, dd, Ar(C6)-*H*]; 9.45 [1H, s, Ar-C*H*=NH$^+$-*t*Bu, $\Delta\nu^1/_2$ = 2.1

Hz]; 11.97 [1H, vbr. s, Ar-CH=N*H*$^+$-*t*Bu, Δν$^1/_2$ = 80 Hz]. **^{11}B NMR** (160.48 MHz, THF-*d*$_8$) δ (ppm): –17.3 [1B, s, *B7* (i.e. Te-*B*)]; –15.8 [5B, overlapping dublets, *B2–B6,* $^1J(^{11}B,^1H)$ = N/A]; –12.5 [4B, overlapping dublets, *B8–B11,* $^1J(^{11}B,^1H)$ = N/A]; –5.8 [1B, d, *B12*, $^1J(^{11}B,^1H)$ = 139.9 Hz]. **^{13}C{^{1}H} NMR** (100.61 MHz, THF-*d*$_8$) δ (ppm): 28.0 [s, (CH_3)$_3$C-]; 53.9 [s, *C*$B_{11}H_{12}^-$]; 62.6 [s, (CH_3)$_3$*C*-]; 122.1 [s, qC, Ar-*C1*(*ipso*)]; 129.4 [s, Ar-*C4*]; 129.5 [s, Ar-*C3*]; 134.7 [s, qC, Ar-*C2*]; 136.0 [s, Ar-*C5*]; 146.3 [s, Ar-*C6*]; 174.6 [s, Ar-*C*H=NH$^+$-*t*Bu]. **^{15}N NMR** (40.54 MHz, THF-*d*$_8$) δ (ppm): –172.1 ppm. **^{125}Te NMR** (126.24 MHz, THF-*d*$_8$) δ: 58.4 ppm.

Solid state sample: **IR** (diamond ATR): 3295 cm^{-1} (NH stretch).

NMR data obtained for dissolved crystals (**5a/5b**)**·toluene** in $CDCl_3$:

12-[2-(*t*BuN{H}CH)C_6H_4Te]$CB_{11}H_{11}$ (5a) (major isomer): **^{1}H NMR** (500.20 MHz, $CDCl_3$) δ (ppm): 1.71 [9H, s, (C*H*$_3$)$_3$C-]; 1.19–2.32 [11H, m, HCB$_{11}$*H*$_{11}^-$]; 2.37 [3H, s, toluene-C*H*$_3$]; 2.52 [1H, s, *H1*-$CB_{11}H_{11}^-$]; 7.17 [1H, t, toluene-*p*-C*H*]; 7.19 [2H, d, toluene-*o*-C*H*]; 7.27 [2H, t, toluene-*m*-C*H*]; 7.46 [1H, td, Ar(C5)-*H*]; 7.56 [1H, td, Ar(C4)-*H*]; 7.61 [1H, dd, Ar(C3)-*H*]; 8.21 [1H, dd, Ar(C6)-*H*]; 8.38 [1H, d, Ar-C*H*=NH$^+$-*t*Bu, $^3J(^1H,^1H)$ = 17.3 Hz]; 13.69 [1H, vbr. m, Ar-CH=N*H*$^+$-*t*Bu, Δν$^1/_2$ = 90 Hz]. **^{11}B NMR** (128.38 MHz, $CDCl_3$) δ (ppm): –15.2 [5B, d, *B2–B6*), $^1J(^{11}B,^1H)$ = N/A]; –12.4 [5B, d, *B7–B11*), $^1J(^{11}B,^1H)$ = N/A]; –11.3 [1B, s, *B12* (i.e. Te-*B*)]. **^{13}C{^{1}H} NMR** (125.78 MHz, $CDCl_3$) δ (ppm): 21.7 [s, toluene-*C*H$_3$]; 28.9 [s, (*C*H$_3$)$_3$C-]; 52.1 [s, *C*$B_{11}H_{12}^-$]; 61.7 [s, (CH_3)$_3$*C*-]; 115.7 [s, qC, Ar-*C1*(*ipso*)]; 125.5 [s, toluene-*p*-*C*H]; 127.8 [s, qC, Ar-*C2*]; 128.4 [2C, s, toluene-*m*-*C*H]; 129.1 [s, Ar-*C4*]; 129.2 [2C, s, toluene-*o*-*C*H]; 136.3 [s, Ar-*C5*]; 138.1 [s, qC, toluene-*C1*(*ipso*)]; 140.0 [s, Ar-*C3*]; 149.3 [s, Ar-*C6*]; 167.3 [s, Ar-*C*H=NH$^+$-*t*Bu]. **^{15}N NMR** (40.54 MHz, $CDCl_3$) δ: –169.8 ppm [d, Ar-CH=*N*H$^+$-*t*Bu, $^1J(^{15}N,^1H)$ = 85.3 Hz]. **^{125}Te NMR** (126.24 MHz, $CDCl_3$) δ: 98.5 ppm.

7-[2-(*t*BuN{H}CH)C_6H_4Te]$CB_{11}H_{11}$ (5b) (minor isomer): **^{1}H NMR** (500.20 MHz, $CDCl_3$) δ (ppm): 1.70 [9H, s, $(CH_3)_3$C-]; 1.19–2.32 [11H, m, $HCB_{11}H_{11}^-$]; 2.37 [3H, s, toluene-CH_3]; 2.50 [1H, s, *H1*-$CB_{11}H_{11}^-$]; 7.17 [1H, t, toluene-*p*-C*H*]; 7.19 [2H, d, toluene-*o*-C*H*]; 7.27 [2H, t, toluene-*m*-C*H*]; 7.49 [1H, td, Ar(C5)-*H*]; 7.57 [1H, td, Ar(C4)-*H*]; 7.65 [1H, dd, Ar(C3)-*H*]; 8.30 [1H, dd, Ar(C6)-*H*]; 8.46 [1H, d, Ar-C*H*=NH^+-*t*Bu, $^3J(^1H,^1H)$ = 17.4 Hz]; 13.47 [1H, vbr. m, Ar-CH=NH^+-*t*Bu, $\Delta\nu^1/_2$ = 79 Hz]. **^{11}B NMR** (128.38 MHz, $CDCl_3$) δ (ppm): -16.8 [1B, s, *B7* (i.e. Te-*B*)]; -15.1 [5B, overlapping doublets, *B2–B6,* $^1J(^{11}B,^1H)$ = N/A]; -12.2 [4B, overlapping doublets, *B8–B11,* $^1J(^{11}B,^1H)$ = N/A]; -5.7 [1B, d, *B12*, $^1J(^{11}B,^1H)$ = 139.9 Hz]. **$^{13}C\{^1H\}$ NMR** (125.78 MHz, $CDCl_3$) δ (ppm): 21.7 [s, toluene-CH_3]; 28.9 [s, $(CH_3)_3$C-]; 54.0 [s, $CB_{11}H_{12}^-$]; 61.9 [s, $(CH_3)_3$*C*-]; 115.4 [s, qC, Ar-*C1*(*ipso*)]; 125.5 [s, toluene-*p*-*C*H]; 128.0 [s, qC, Ar-*C2*]; 128.4 [2C, s, toluene-*m*-*C*H]; 129.4 [s, Ar-*C4*]; 129.2 [2C, s, toluene-*o*-*C*H]; 136.5 [s, Ar-*C5*]; 138.1 [s, qC, toluene-*C1*(*ipso*)]; 139.6 [s, Ar-*C3*]; 149.2 [s, Ar-*C6*]; 167.7 [s, Ar-*C*H=NH^+-*t*Bu]. **^{15}N NMR** (40.54 MHz, $CDCl_3$) δ: -169.8 ppm [d, Ar-CH=*N*H^+-*t*Bu, $^1J(^{15}N,^1H)$ = 85.3 Hz]. **^{125}Te NMR** (126.24 MHz, $CDCl_3$) δ: 65.5 ppm.

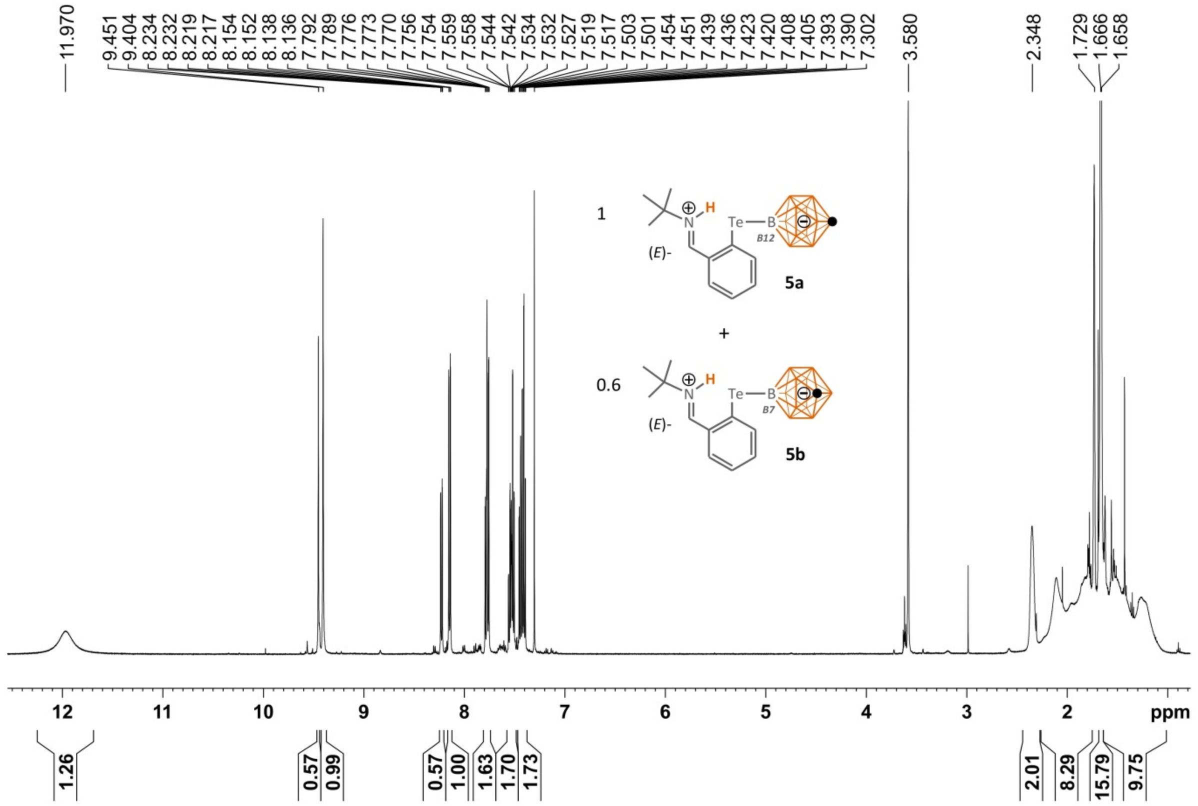

Figure S30. ^{1}**H NMR** spectrum of a mixture of **5a** and **5b** (400.13 MHz, THF-d_8, 294 K).

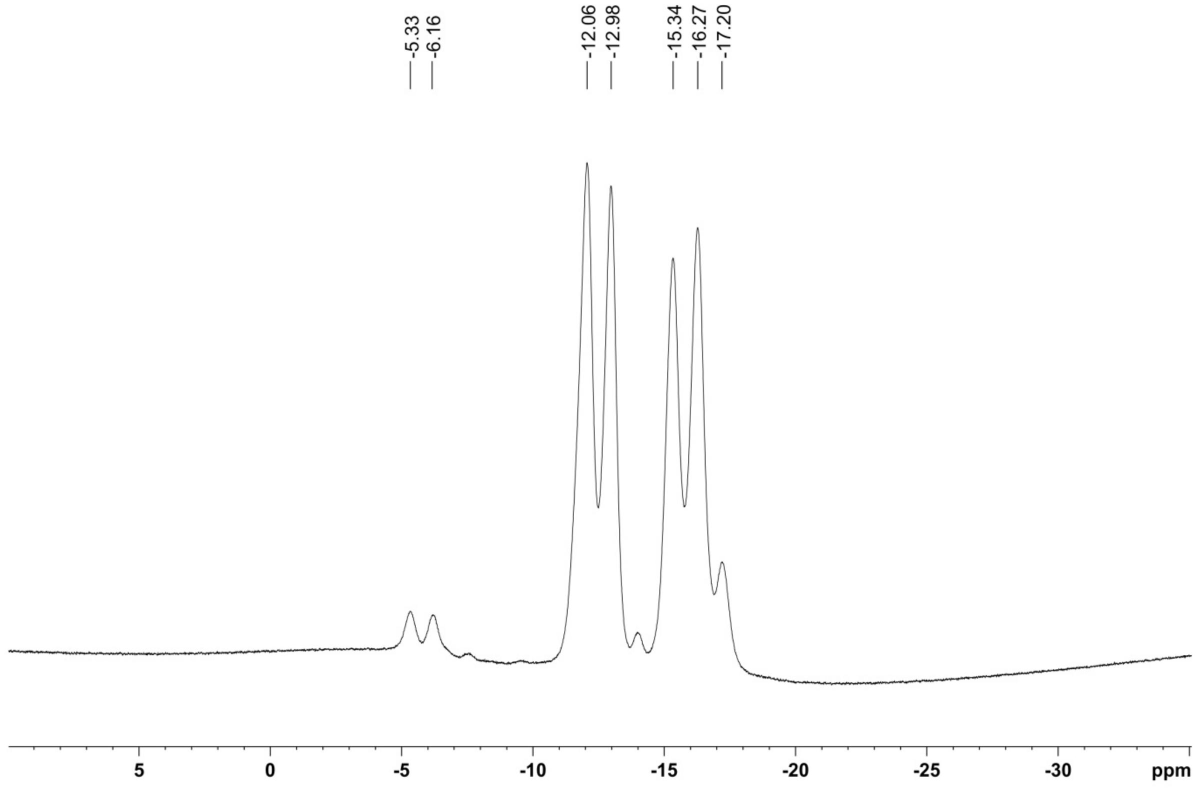

Figure S31. ^{11}**B NMR** spectrum of a mixture of **5a** and **5b** (160.48 MHz, THF-d_8, 294 K).

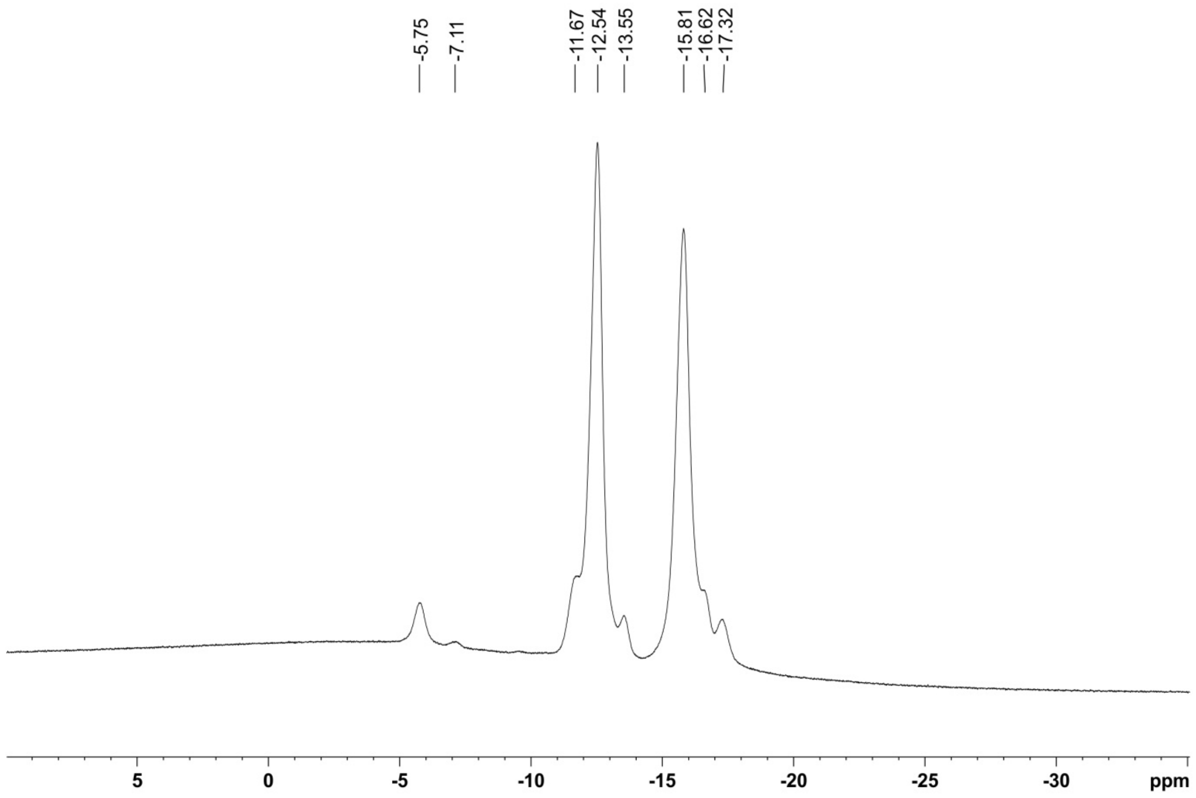

Figure S32. ^{11}**B{**^{1}**H} NMR** spectrum of a mixture of **5a** and **5b** (160.48 MHz, THF-d_8, 294 K).

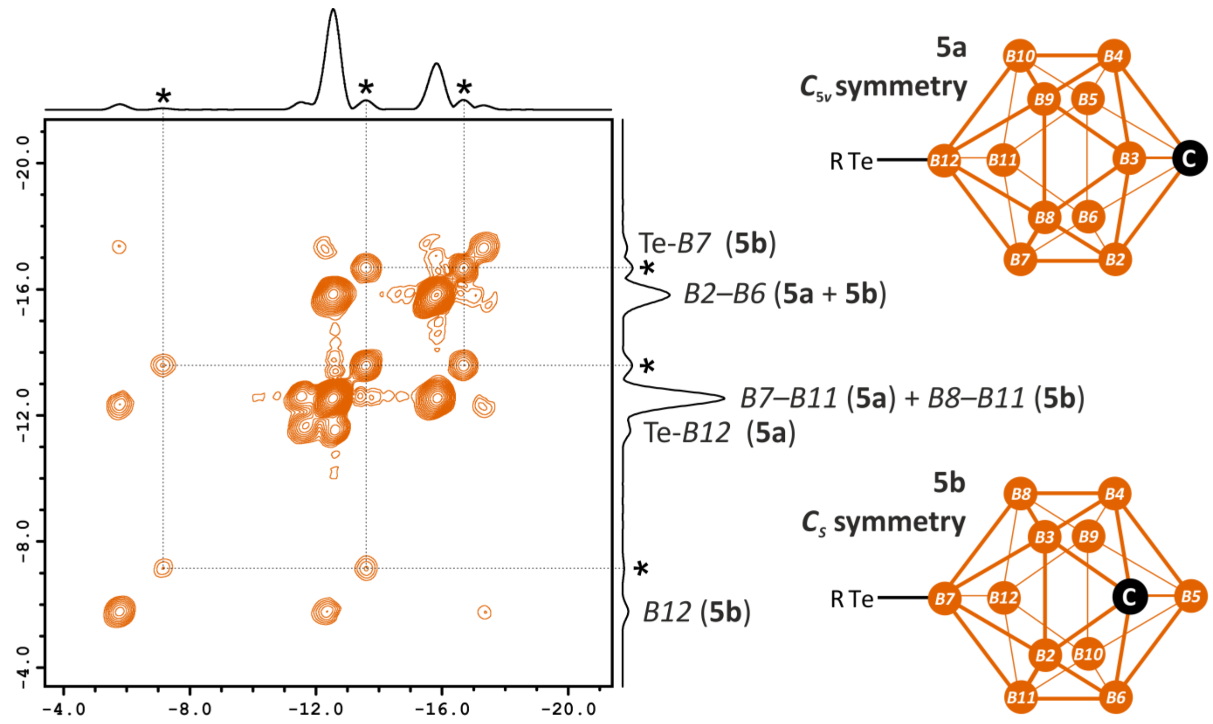

Figure S33. ^{11}B{^{1}H}-^{11}B{^{1}H} COSY NMR spectrum (192.56 MHz, THF-d_8, 294 K) of **5a** and **5b** regioisomers mixture in 1 : 0.6 molar ratio. * Traces of unsubstituted $CB_{11}H_{12}^{-}$ anion with C_{5v} symmetry as impurity.

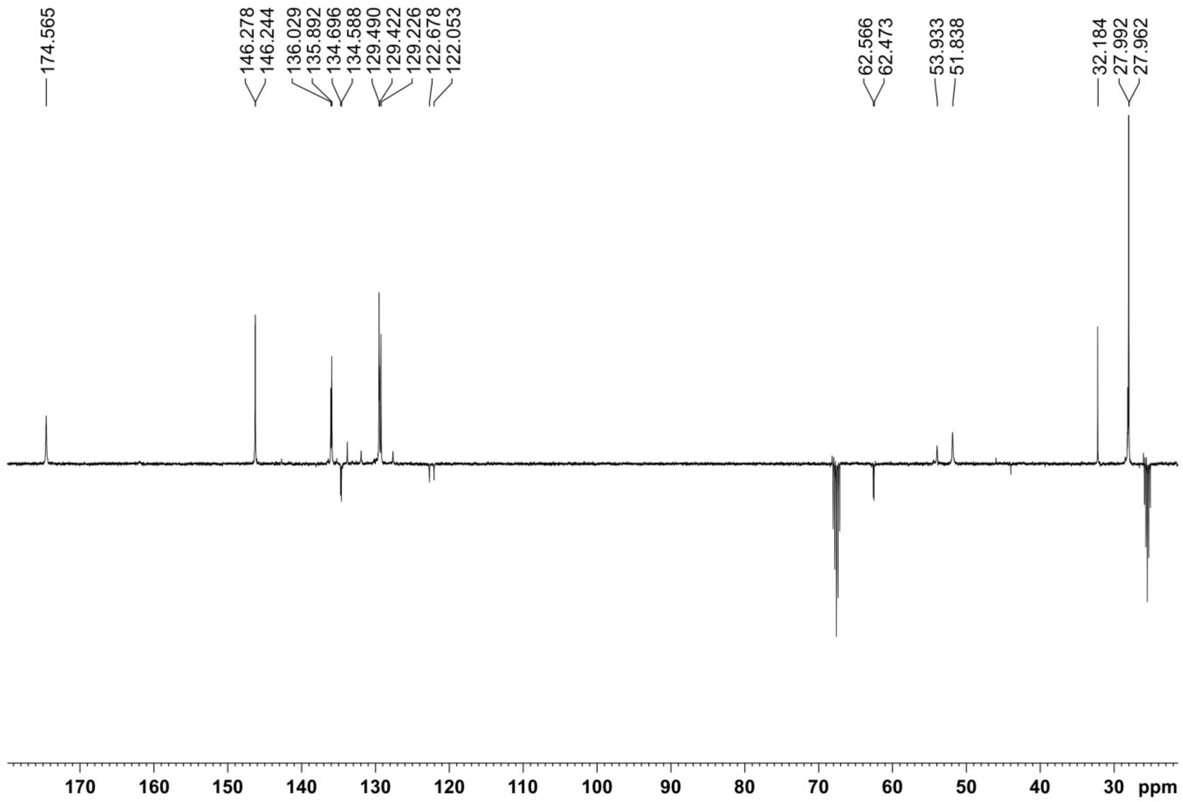

Figure S34. $^{13}C\{^1H\}$ **APT NMR** spectrum of mixture of **5a** and **5b** (100.61 MHz, THF-d_8, 294 K).

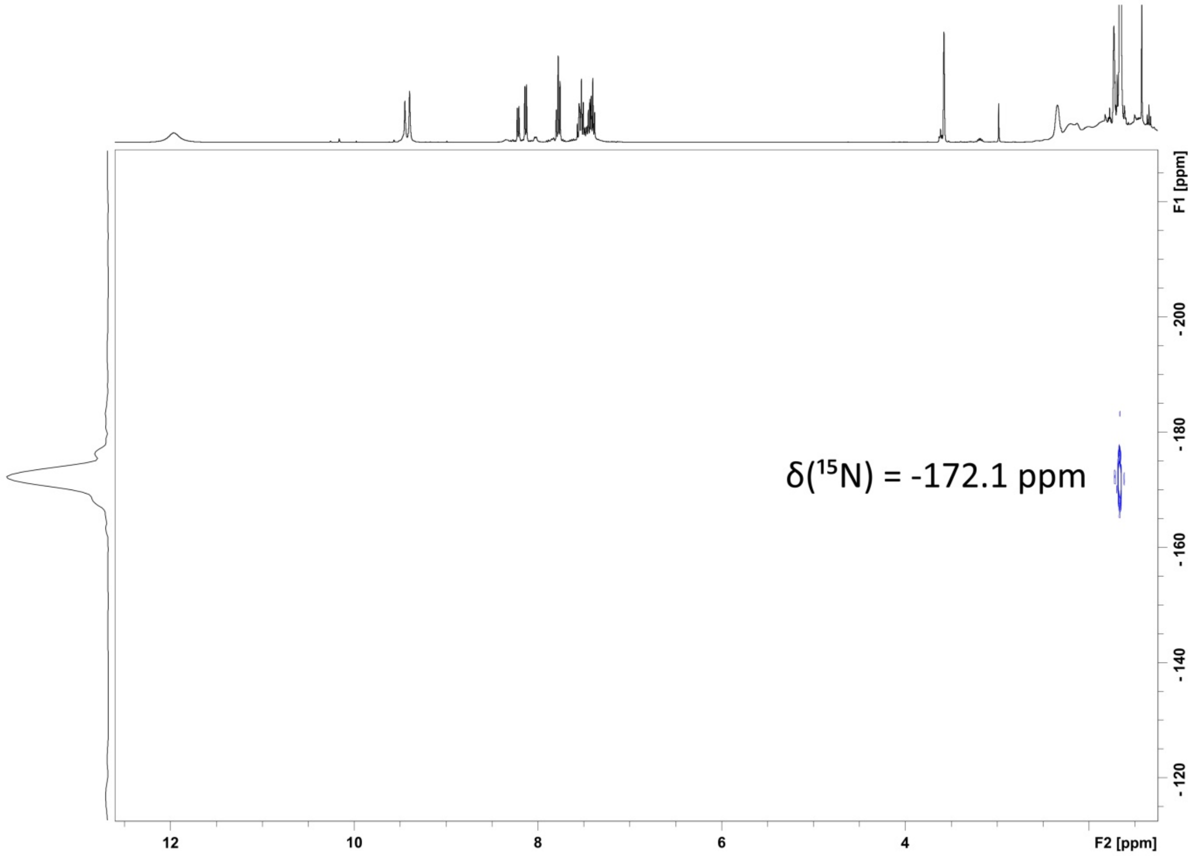

Figure S35. ^1H-^{15}N **HMBC** NMR spectrum of a mixture of **5a** and **5b** (400.13 MHz, THF-d_8, cnst13 = 4 Hz, 294 K).

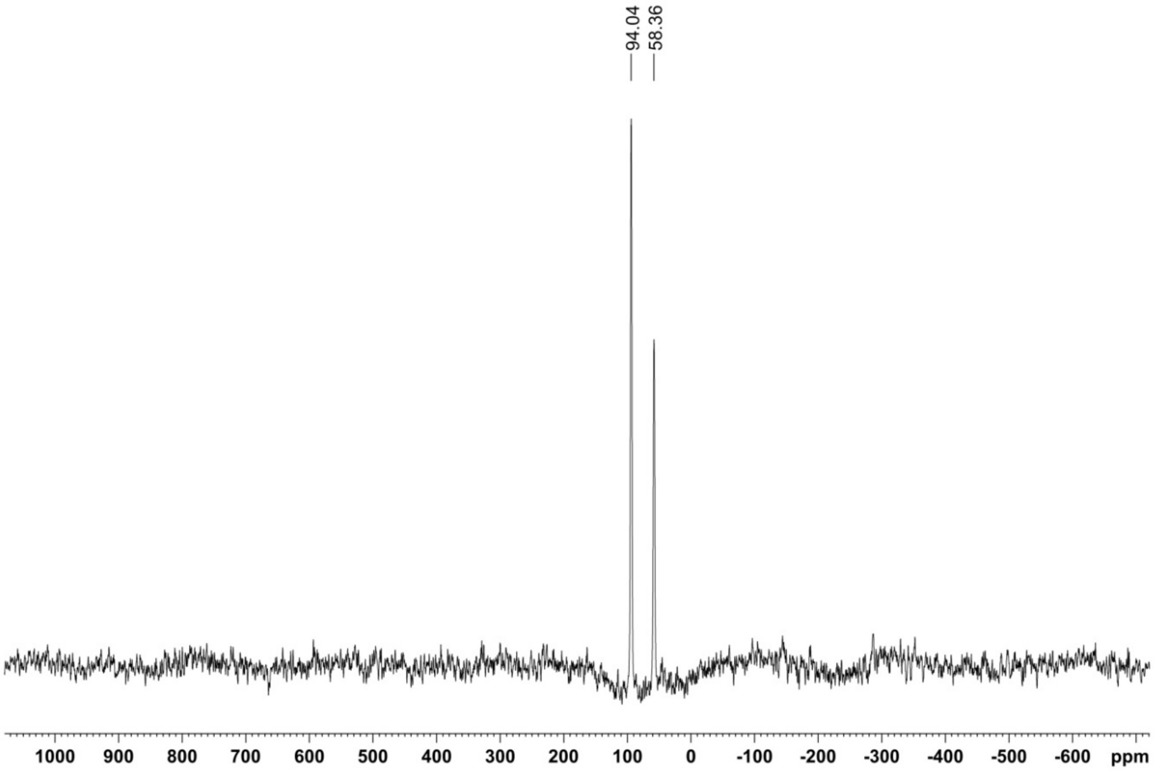

Figure S36. [125]**Te NMR** spectrum of mixture of **5a** and **5b** (126.24 MHz, THF-d_8, 294 K, NS = 76800).

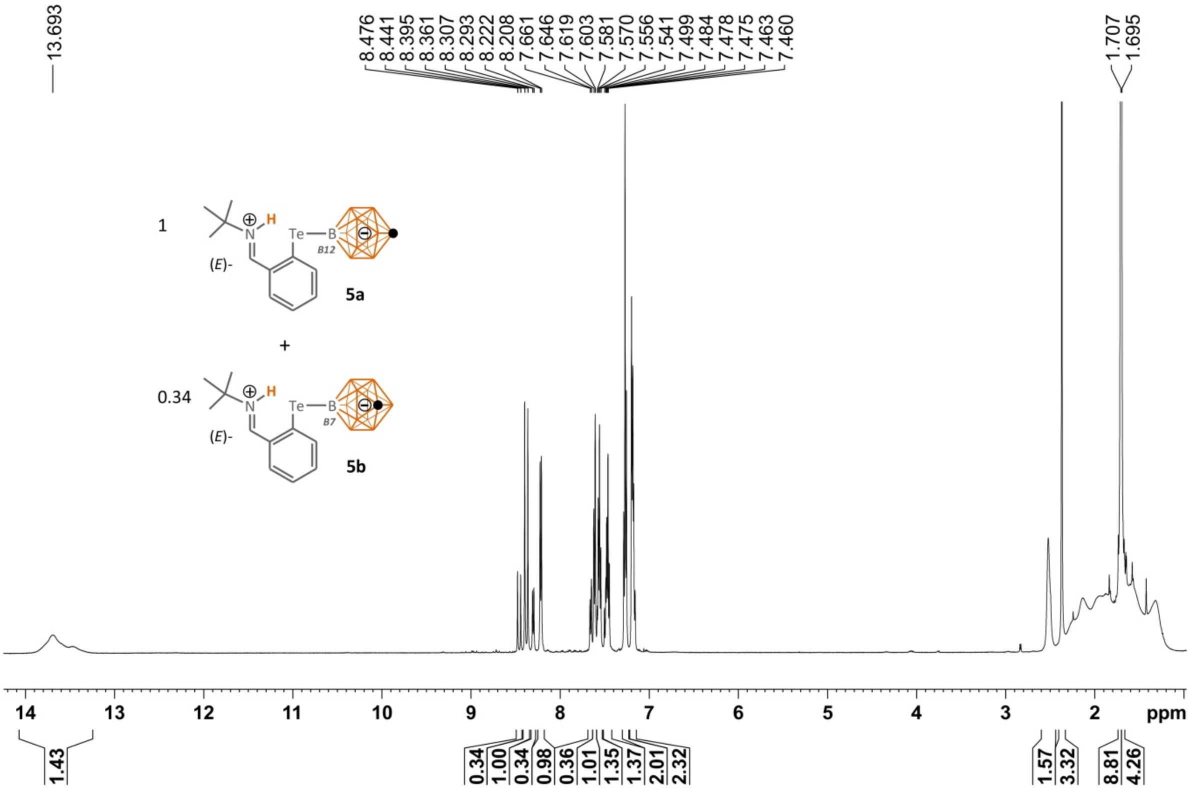

Figure S37. [1]**H NMR** spectrum of crystals containing **5a** and **5b** co-crystalized with toluene (500.20 MHz, $CDCl_3$, 294 K).

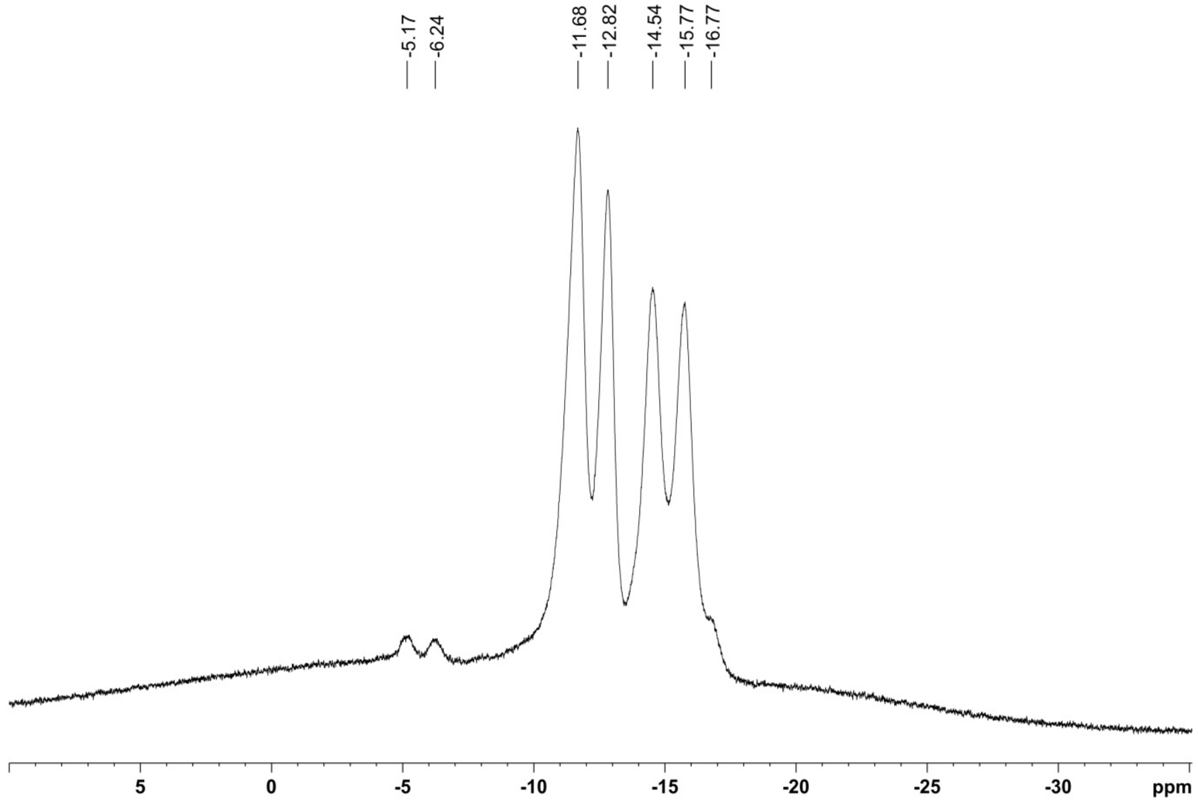

Figure S38. ^{11}**B NMR** spectrum of crystals containing **5a** and **5b** co-crystallized with toluene (128.38 MHz, $CDCl_3$, 294 K).

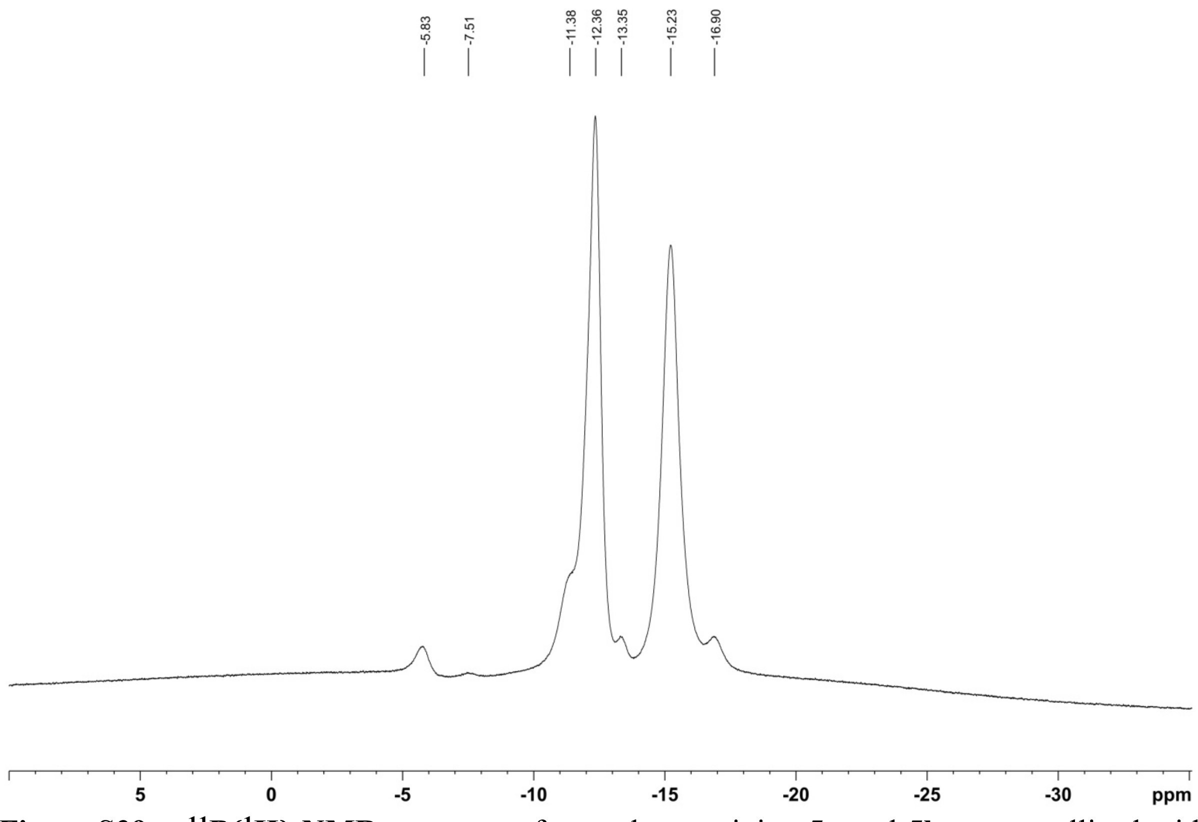

Figure S39. ^{11}**B{**^{1}**H} NMR** spectrum of crystals containing **5a** and **5b** co-crystallized with toluene (128.38 MHz, $CDCl_3$, 294 K).

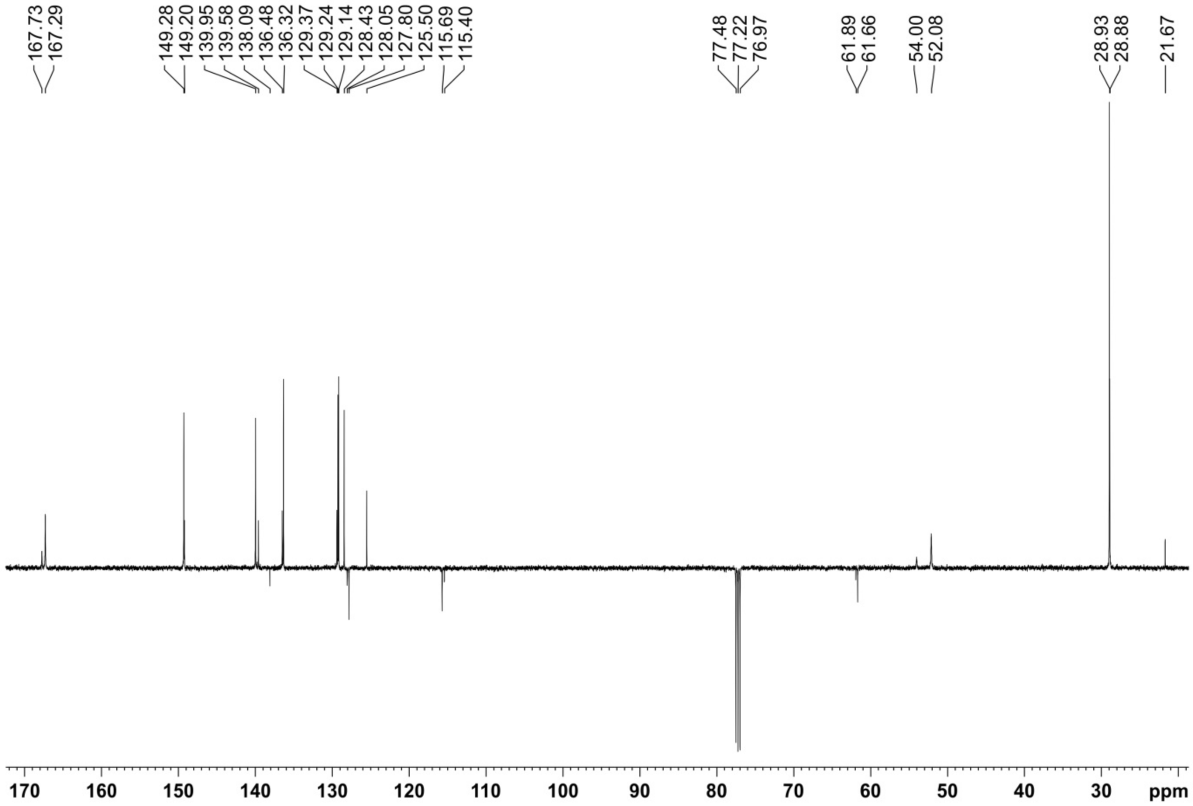

Figure S40. **$^{13}C\{^1H\}$ APT NMR** spectrum of crystals containing **5a** and **5b** co-crystallized with toluene (125.78 MHz, $CDCl_3$, 294 K.

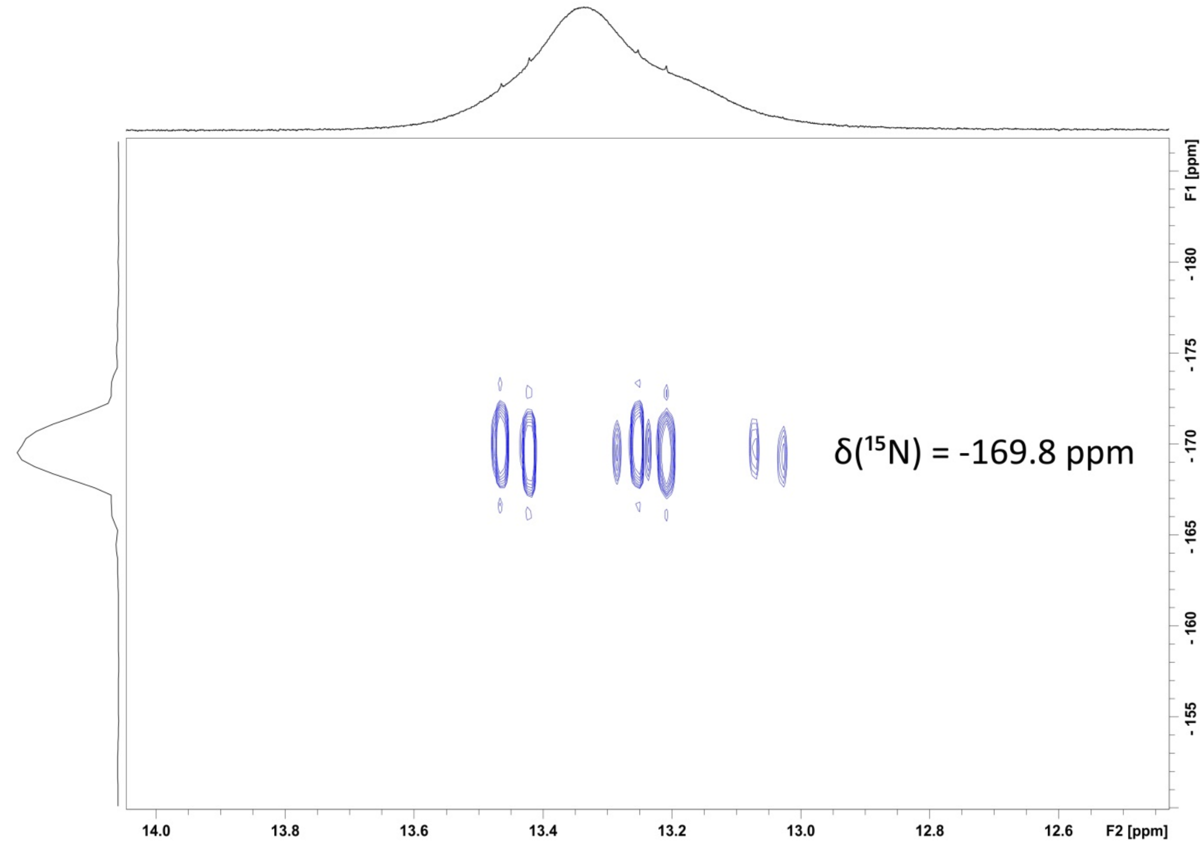

Figure S41. **^1H-^{15}N HMBC** NMR spectrum of crystals containing **5a** and **5b** co-crystallized with toluene (400.13 MHz, $CDCl_3$, cnst13 = 4 Hz, 294 K).

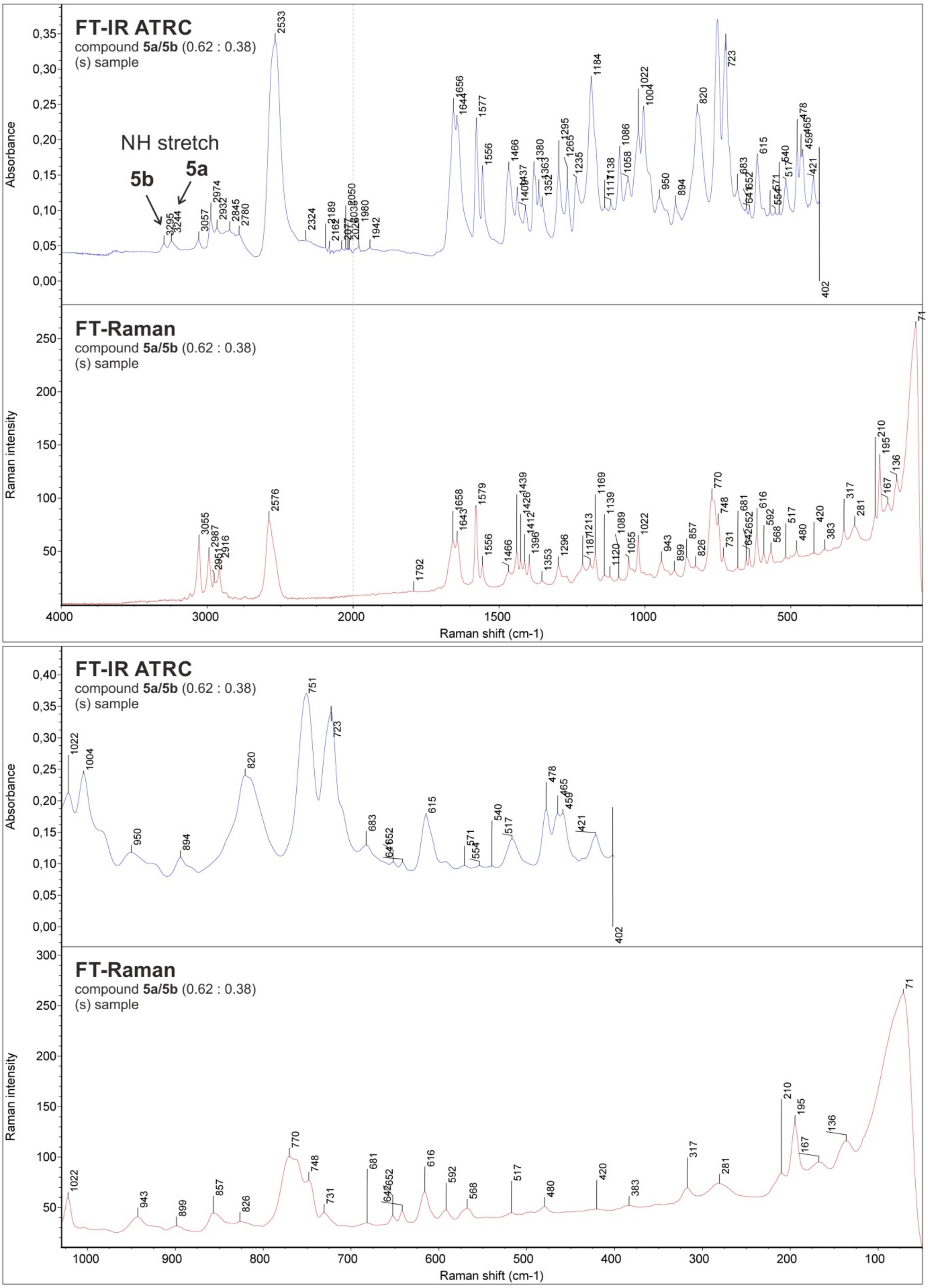

Figure S42. FT-IR ATRC spectra and FT-Raman emission spectra of compound **5a/5b**.

Proof that protonated imino bond CH=NH$^+$ in 5a and 5b has *E*- configuration in both cases

The best resolution in ^{1}H-^{15}N HMBC spectrum (bellow) was obtained when **5a** and **5b** were measured in CD_2Cl_2 since in this solvent the signals of CH=N***H***$^+$ protons of both isomers are most separated in ^{1}H ppm axis.

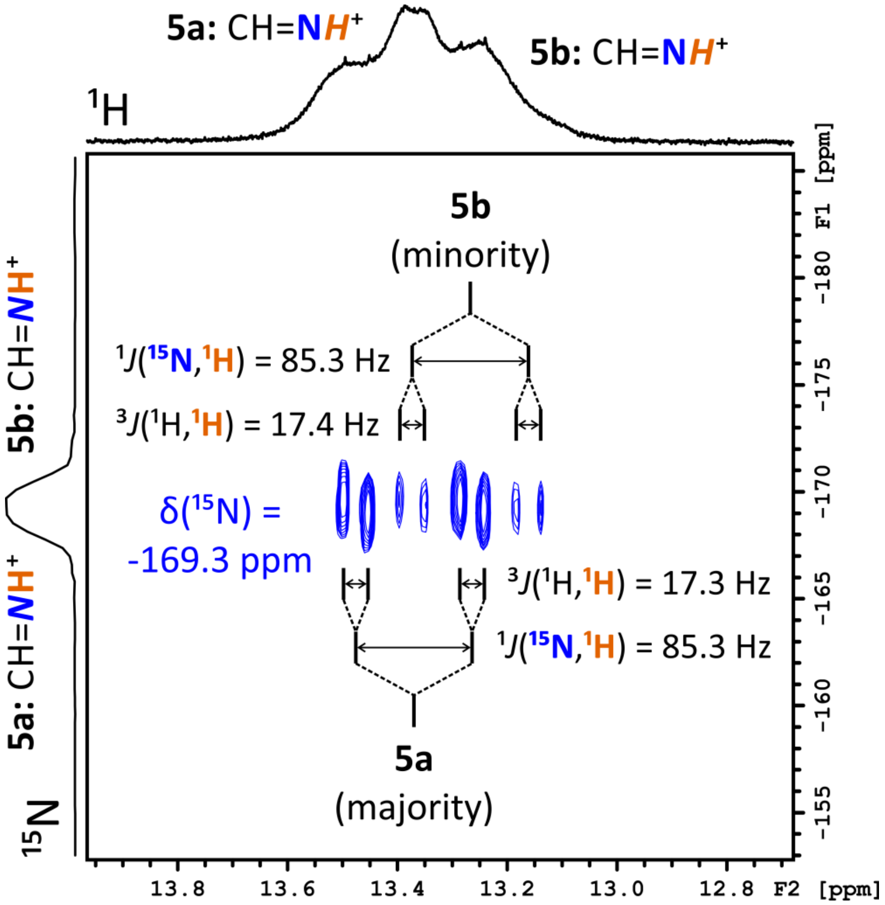

Figure S43. Cut-out of ^{1}H-^{15}N HMBC NMR spectrum (400.13 MHz, CD_2Cl_2, cnst13 = 4 Hz, 294 K) of **5a** and **5b** regioisomers mixture in 1 : 0.2 molar ratio showing the crucial region of the spectrum.

Note: It turned out, that change of non-coordinating solvent to coordinating solvent results in huge difference in δ(^{13}C) of Ar-***C1***(*ipso*), Ar-***C2***, Ar-***C3*** and imino ***C***H=NH$^+$ carbon (*i.e degree of dissociation of CH=N**H**$^+$ proton affects δ(^{13}C) of these carbons, see Table S3 below*). This observation seems to be result of very different degree of the Ar(***C2***)---***C***H=NH$^+$

bond order in the dependence on N*H*$^+$ acidity – dissociation of H^+ in coordinating/non-coordinating solvent.

Table S3. δ(^{13}C) NMR of **5a** [ppm] in non-coordinating/coordinating solvent.

	**in $CDCl_3$**	**in THF-*d*$_8$**
Ar-***C1***(*ipso*)-Te	115.7	122.7
Ar-***C2***-CH=NH$^+$*t*Bu	127.8	134.6
Ar-***C***H=NH$^+$*t*Bu	167.3	174.6
Ar-***C3***	140.0	129.5

Synthesis of [Et_3NH][12-{2-(*t*BuNCH)C_6H_4Te}$CB_{11}H_{11}$] (6a) and [Et_3NH][7-{2-(*t*BuNCH)C_6H_4Te}$CB_{11}H_{11}$] (6b).

The mixture of regioisomers **5a** and **5b** in mutual ratio **5a** : **5b** = 0.62 : 0.38 (256 mg, 0.59 mmol) prepared according to the procedure described above was dissolved in THF (10 mL) in a Schlenk tube under formation of a blood-red solution. Neat triethylamine (83 μL, 60 mg, 0.59 mmol) was added into this solution under stirring and the color change from blood-red to much less intense light-orange suddenly occurred (See UV/Vis spectrum at Figure S51). The reaction mixture was evaporated under the formation of an orange oil in quantitative yield of composition **6a** and **6b** with (according to ^{1}H NMR analysis) the exactly same mutual ratio as given by starting stereoisomers **5a** and **5b**. Unfortunately, all attempts to crystallize **6a** or **6b** remained unsuccessful probably due to their inherent ionic liquid character.

NMR data for [Et_3NH][12-{2-(*t*BuNCH)C_6H_4Te}$CB_{11}H_{11}$] (6a) (major isomer) in THF-d_8:

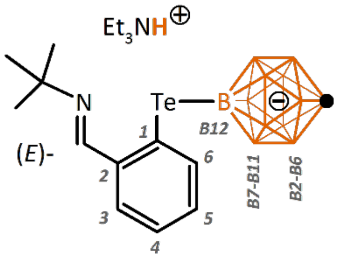

^{1}H NMR (500.20 MHz, THF-d_8) δ (ppm): 1.21 [9H, t, (CH_3-CH_2)$_3$$NH^+$]; 1.28 [9H, s, (C$H_3$)$_3$C-]; 1.33–2.25 [11H, m, $HCB_{11}$$H_{11}^-$]; 2.33 [1H, s, *H1*-$CB_{11}H_{11}^-$]; 3.02 [6H, q, ($CH_3$-C$H_2$)$_3$$NH^+$]; 6.95 [1H, td, Ar(C5)-*H*]; 7.11 [1H, t, Ar(C4)-*H*]; 7.45 [1H, br. s, (CH_3-CH_2)$_3$NH^+, $\Delta\nu^1/_2$ = 11.5 Hz]; 7.83 [1H, dd, Ar(C3)-*H*]; 7.99 [1H, dd, Ar(C6)-*H*]; 8.76 [1H, s, Ar-C*H*=N-*t*Bu]. **^{11}B NMR** (160.48 MHz, THF-d_8) δ (ppm): -16.0 [5B, d, *B2–B6*), $^1J(^{11}B,^1H)$ = N/A]; -12.2 [5B, d, *B7–B11*), $^1J(^{11}B,^1H)$ = N/A]; -11.8 [1B, s, *B12* (i.e. Te-*B*)]. **^{13}C{^{1}H} NMR** (125.78 MHz, THF-d_8) δ (ppm): 10.4 [3C, s, (*C*H_3-CH_2)$_3$$NH^+$]; 30.5 [s, (*C*$H_3$)$_3$C-]; 48.0 [3C, s, ($CH_3$-*C*$H_2$)$_3$$NH^+$]; 51.7 [s, *C*$B_{11}H_{12}^-$]; 58.1 [s, ($CH_3$)$_3$*C*-]; 118.9 [br. s, qC, Ar-*C1*(*ipso*)]; 127.0 [s, Ar-*C3*]; 127.0 [s, Ar-*C4*]; 129.9 [s, Ar-*C5*]; 141.5 [s, qC, Ar-*C2*]; 143.4 [s, Ar-*C6*]; 163.0 [s, Ar-*C*H=N-*t*Bu]. **^{15}N NMR** (50.70 MHz, THF-d_8) δ (ppm): -326.4 [Et_3*N*H^+]; -35.4 [CH=*N*-*t*Bu]. **^{125}Te NMR** (126.24 MHz, THF-d_8) δ: -3.2 ppm.

NMR data for [Et_3NH][7-{2-(*t*BuNCH)C_6H_4Te}$CB_{11}H_{11}$] (6b) (minor isomer) in THF-d_8:

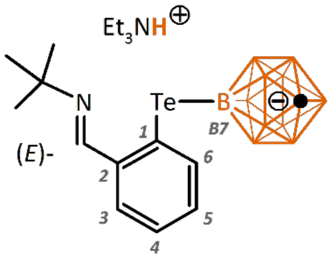

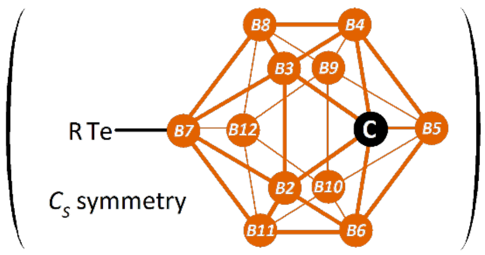

^{1}H NMR (500.20 MHz, THF-d_8) δ (ppm): 1.21 [9H, t, (CH_3-CH_2)$_3$$NH^+$]; 1.28 [9H, s, (C$H_3$)$_3$C-]; 1.33–2.25 [11H, m, $HCB_{11}$$H_{11}^-$]; 2.28 [1H, s, *H1*-$CB_{11}H_{11}^-$]; 3.02 [6H, q, ($CH_3$-

$CH_2)_3NH^+$]; 6.96 [1H, td, Ar(C5)-*H*]; 7.14 [1H, t, Ar(C4)-*H*]; 7.45 [1H, br. s, (CH_3-$CH_2)_3NH^+$, $\Delta\nu^1/_2$ = 11.5 Hz]; 7.87 [1H, dd, Ar(C3)-*H*]; 8.05 [1H, dd, Ar(C6)-*H*]; 8.84 [1H, s, Ar-C*H*=N-*t*Bu]. **^{11}B NMR** (160.48 MHz, THF-d_8) δ (ppm): -17.7 [1B, s, *B7* (i.e. Te-*B*)]; -16.7 – -14.9 [5B, overlapping dublets, *B2–B6,* $^1J(^{11}B,^1H)$ = N/A]; -13.1 – -11.7 [4B, overlapping dublets, *B8–B11,* $^1J(^{11}B,^1H)$ = N/A]; -5.5 [1B, d, *B12*, $^1J(^{11}B,^1H)$ = 139.5 Hz]. **$^{13}C\{^1H\}$ NMR** (125.78 MHz, THF-d_8) δ (ppm): 10.4 [3C, s, (CH_3-$CH_2)_3NH^+$]; 30.5 [s, ($CH_3)_3C$-]; 48.0 [3C, s, (CH_3-$CH_2)_3NH^+$]; 53.6 [s, $CB_{11}H_{12}^-$]; 58.0 [s, ($CH_3)_3C$-]; 118.1 [br. s, qC, Ar-*C1*(*ipso*)]; 127.0 [s, Ar-*C3*]; 127.2 [s, Ar-*C4*]; 129.9 [s, Ar-*C5*]; 141.3 [s, qC, Ar-*C2*]; 143.8 [s, Ar-*C6*]; 163.1 [s, Ar-*C*H=N-*t*Bu]. **^{15}N NMR** (50.70 MHz, THF-d_8) δ (ppm): -326.4 [Et_3NH^+]; -32.8 [CH=*N*-*t*Bu]. **^{125}Te NMR** (126.24 MHz, THF-d_8) δ: -42.5 ppm.

Compounds **6a/6b** are highly soluble in THF and CH_2Cl_2, but virtually insoluble in aliphatic and aromatic solvents.

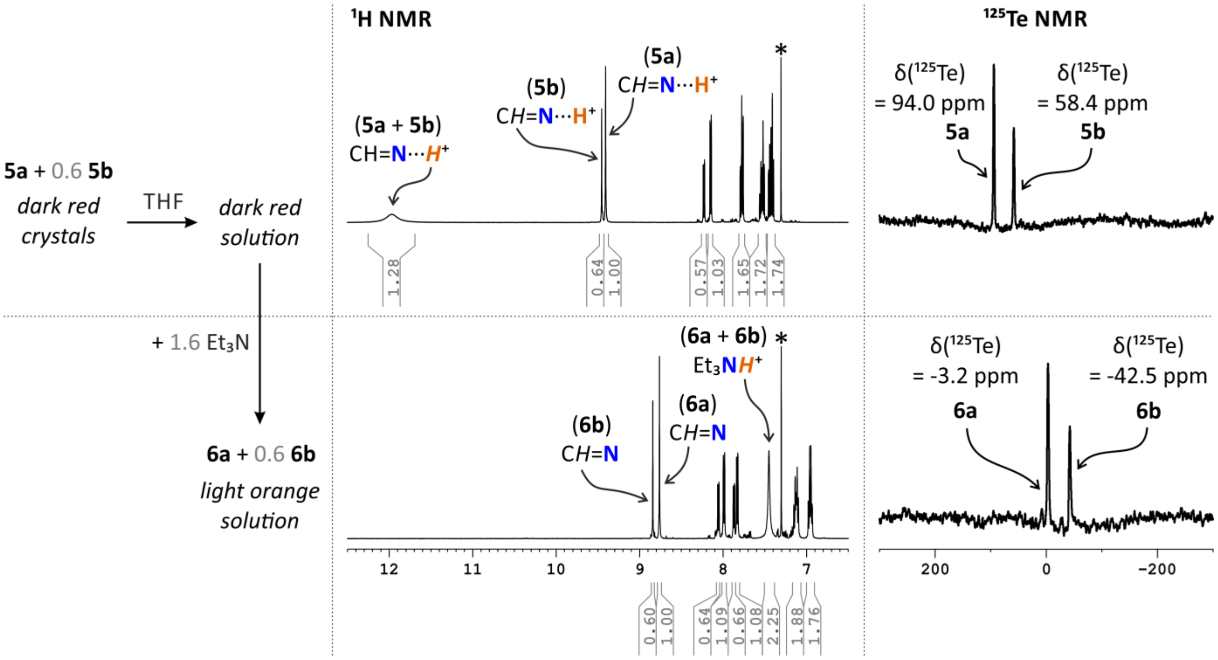

Figure S44. Stacked plot of cut-outs of ^{1}H NMR spectra (500.20 MHz, 294 K, THF-d_8) in the region 6.5 – 12.5 ppm and of corresponding ^{125}Te NMR spectra (126.24 MHz, 294 K, THF-d_8) in the region –300 – 300 ppm showing changes in spectra after deprotonation of compounds **5a**/**5b** when Et_3N was added. * trace amounts of benzene.

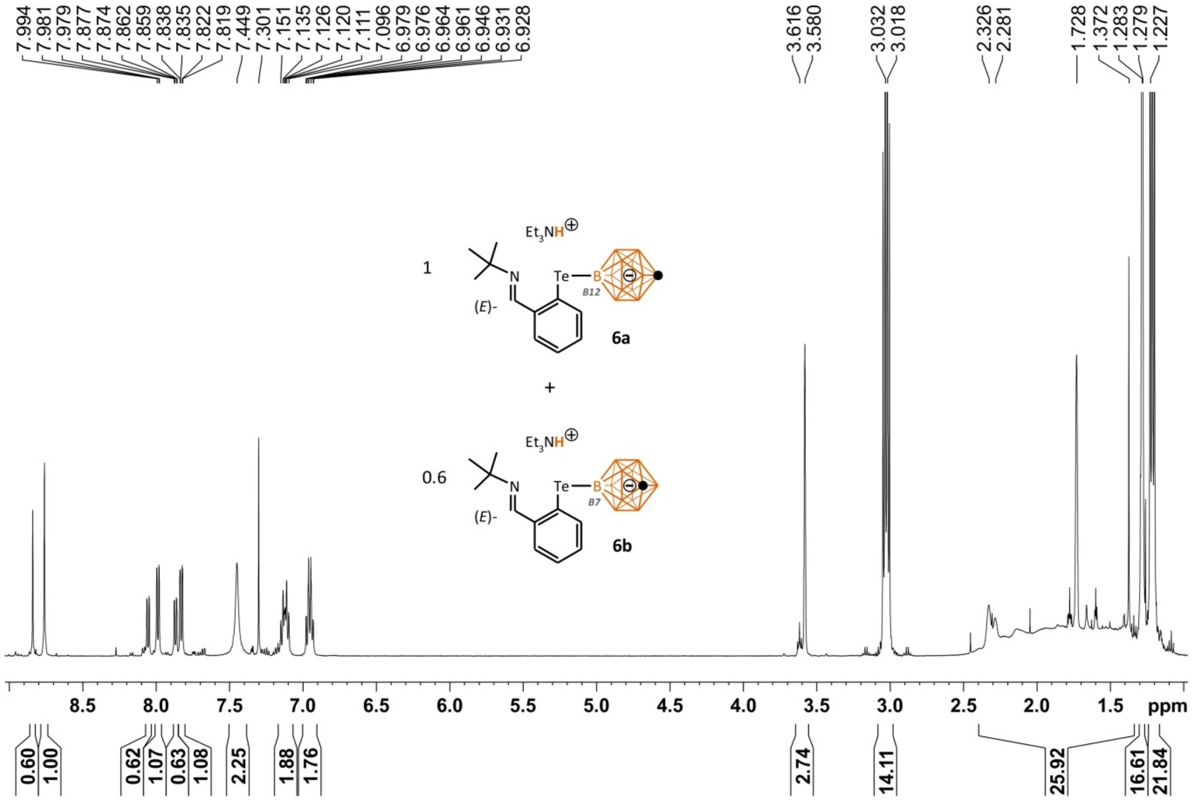

Figure S45: ^{1}**H NMR** of a mixture of compounds **6a** and **6b** (500.20 MHz, THF-d_8, 294 K).

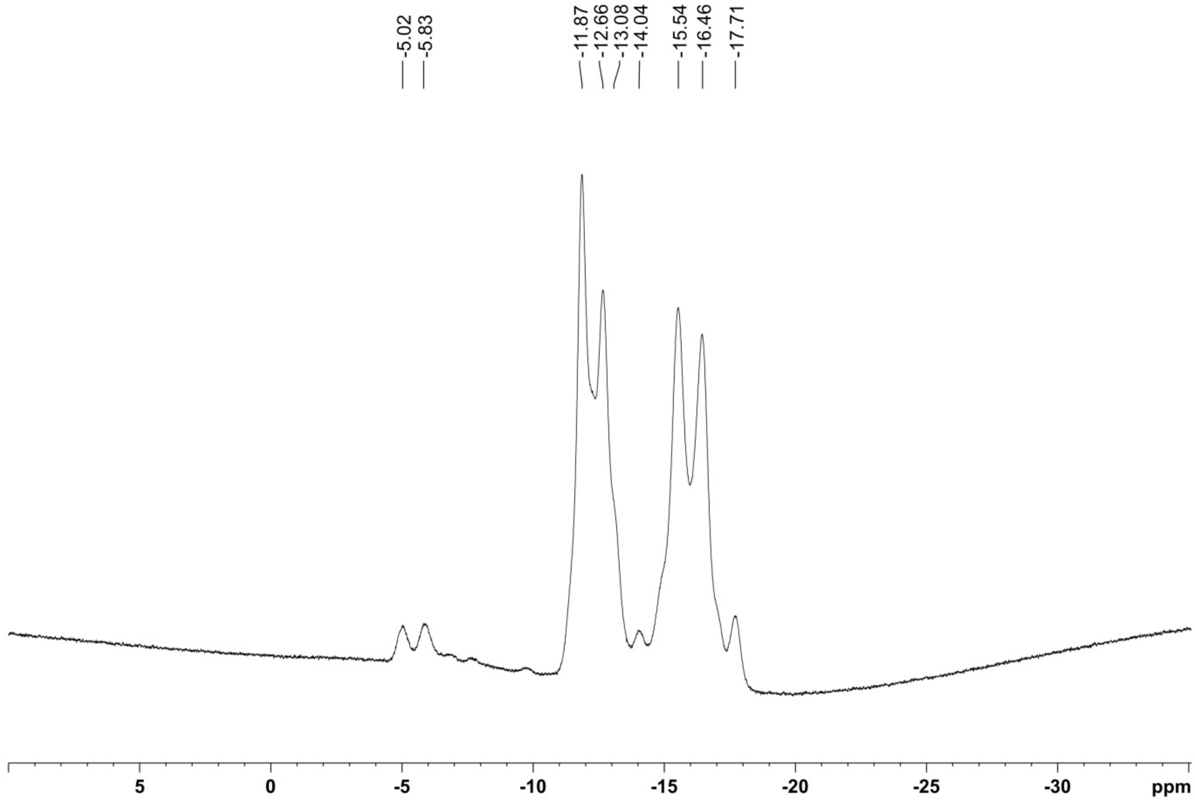

Figure S46. ^{11}**B NMR** spectrum of a mixture of **6a** and **6b** (160.48 MHz, THF-d_8, 294 K).

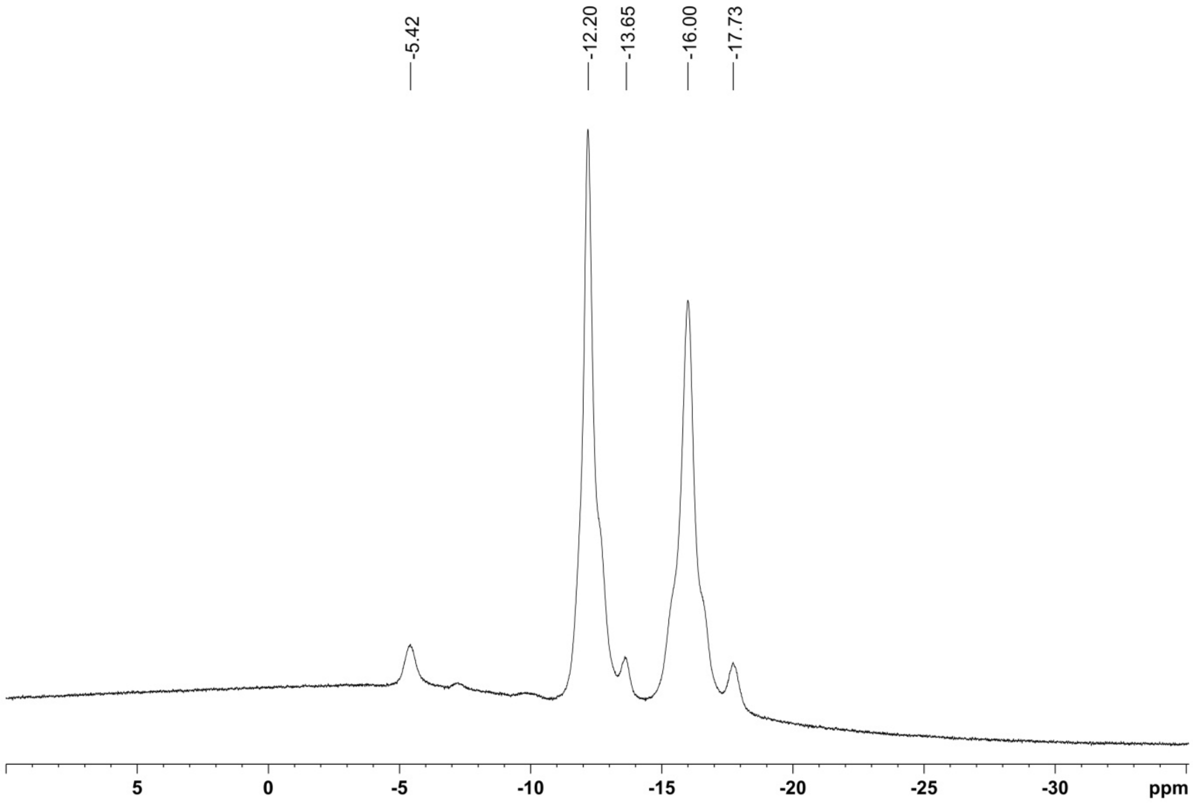

Figure S47. ^{11}B{^{1}H} NMR spectrum of a mixture of **6a** and **6b** (160.48 MHz, THF-d_8, 294 K).

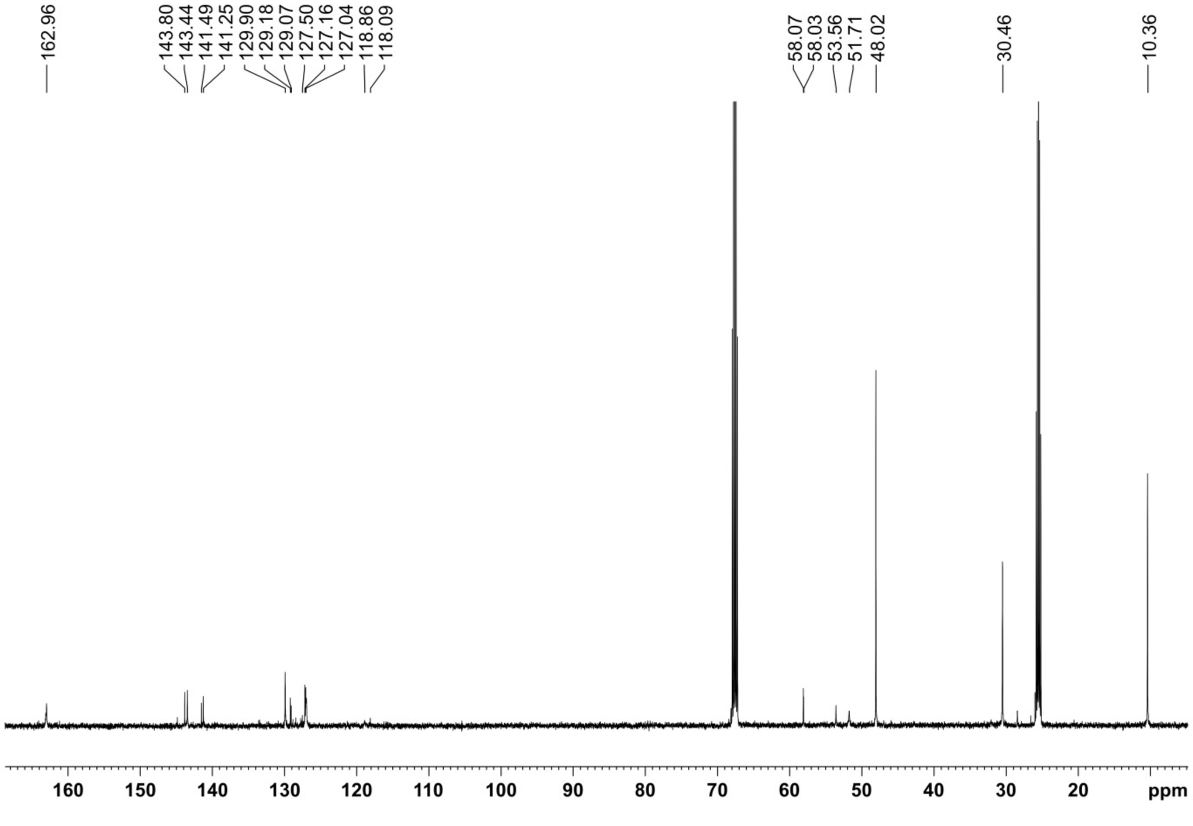

Figure S48. $^{13}C\{^{1}H\}$ **NMR** spectrum of a mixture of **6a** and **6b** (125.78 MHz, THF-d_8, 294 K).

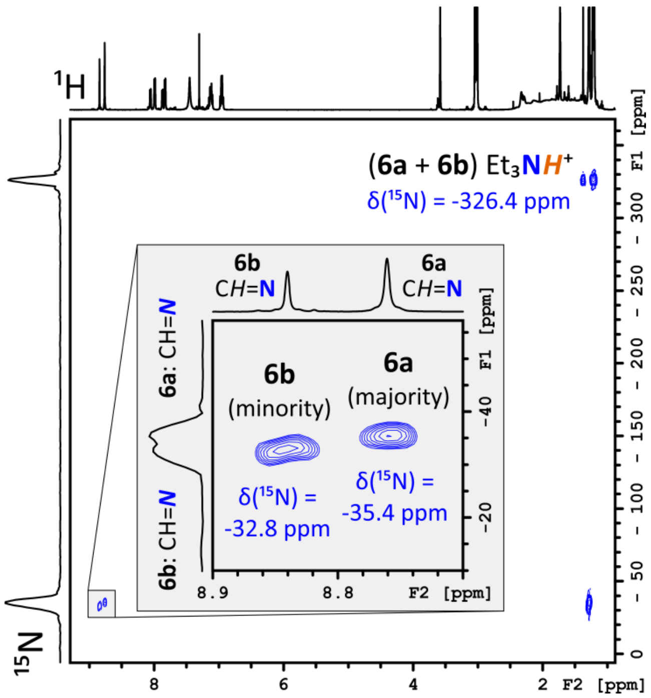

Figure S49. ^{1}H-^{15}N **HMBC** spectrum (500.20 MHz, THF-d_8, cnst13 = 4 Hz, 294 K) with zoomed crucial region of the spectrum of **6a** and **6b** mixture in molar ratio 1 : 0.6.

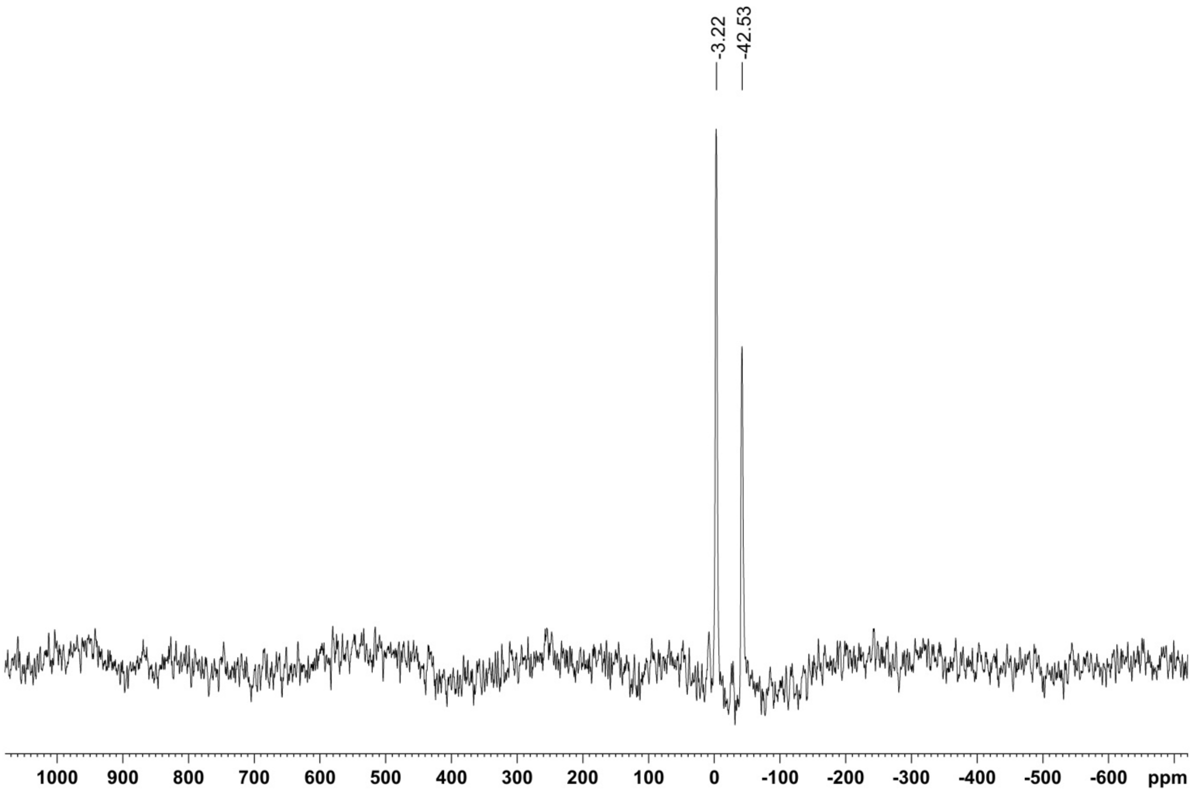

Figure S50. ^{125}**Te NMR** spectrum of a mixture of **6a** and **6b** (126.24 MHz, THF-d_8, 294 K, NS = 76800).

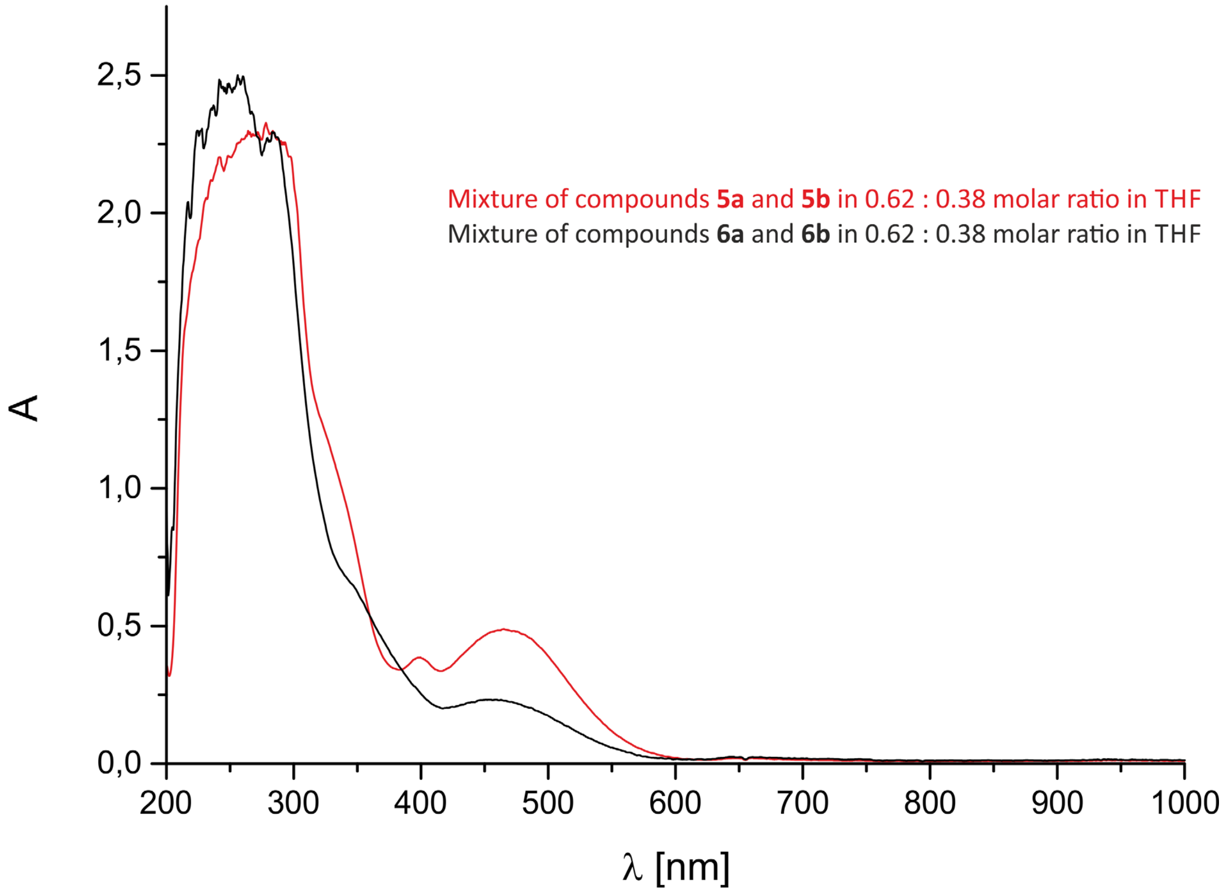

Figure S51. UV/Vis spectra of compounds **5a/5b** and **6a/6b**. Compounds **5a/5b**: λ_{max} = 466 nm (ε = 2319 l mol^{-1} cm^{-1}), compounds **6a/6b**: λ_{max} = 457 nm (ε = 753 l mol^{-1} cm^{-1}).

Proof that 5a and 5b regioisomers crystallizes together in one crystal lattice

We selected one bigger single-crystal of which lattice parameters were checked by XRD to be the same as obtained for the solved solid state structure showed in Figure 2. Then the single-crystal was dissolved in [D8]THF and ^{1}H NMR spectra were acquired. Despite a single crystal was taken, this NMR spectrum showed signals for both **5a** and **5b** regioisomers. Due to this fact, **5a** obviously cannot be separated from **5b** by crystallization.

Crystallography

Intensity data of **1**·1/2 CH_2Cl_2, **2a**(**2b**), **2a**, **3**, **4** and **5a**(**5b**)·1/2 toluene were collected using a Bruker Venture D8 diffractometer at 100 K with graphite-monochromated Mo-Kα (0.7107 Å) radiation. All structures were solved by direct methods and refined based on F^2 by use of the SHELX program package as implemented in WinGX.[S1] All non-hydrogen atoms were refined using anisotropic displacement parameters. Hydrogen atoms attached to carbon and boron atoms were included in geometrically calculated positions using a riding model. The ratio of **2a**:**2b** and **5a**:**5b** of 62:38 was inferred by refinement of split occupancies for B6(0.375) / C6(0.625), B26(0.925) /C26(0.075), B27(0.925) /C27(0.075), B28(0.925) /C28(0.075), B29(0.925) /C29(0.075) and B30(0.925) /C30(0.075). Disorder was resolved for C3, C4 and C5 of **5a**(**5b**)·1/2 toluene and refined with split occupancies of 0.63 : 0.37. Crystal and refinement data are collected in Table S5. Figures were created using DIAMOND.[S2] Crystallographic data (excluding structure factors) for the structural analyses have been deposited with the Cambridge Crystallographic Data Centre. Copies of this information may be obtained free of charge from The Director, CCDC, 12 Union Road, Cambridge CB2 1EZ, UK (Fax: +44-1223-336033; e-mail: deposit@ccdc.cam.ac.uk or http://www.ccdc.cam.ac.uk).

Table S4. Selected bond parameters [Å, °] of **5a**, **6a**, **7a** and **8a**.

	**5a**<br>X = O1	**6a**<br>X = F1	**7a**<br>X = O1	**8a**<br>X = Te2
Te1-C10	2.073(1)	2.073(2)	2.079(2)	2.103(1)
Te1-N1	2.113(1)	2.076(1)	2.125(2)	2.228(1)
Te1-X	2.500(1)	2.687(2)	2.403(1)	3.034(1)
C1-C11	1.435(2)	1.436(2)	1.431(2)	1.438(2)
C1-N1	1.297(2)	1.301(2)	1.296(2)	1.285(2)
N1-Te1-X	162.69(4)	168.60(5)	164.09(5)	168.93(3)
N1-C1-C11	117.7(1)	118.1(2)	118.4(1)	119.1(1)

Table S5. Crystal data and structure refinement of **1**, **2a(2b)**, **3**, **4** and **5a(5b)**.

	**1**·1/2 CH_2Cl_2	**2a(2b)**
Formula	$C_{12.5}H_{27}B_{11}ClNTe$	$C_{24}H_{52}B_{22}N_2Te_2$
Formula weight, g mol^{-1}	473.31	861.70
Crystal system	Monoclinic	Triclinic
Crystal size, mm	0.08 × 0.05 × 0.05	0.08 × 0.08 × 0.08
Space group	C_2	$P\bar{1}$
a, Å	20.5088(6)	11.3356(2)
b, Å	8.3218(2)	12.9085(3)
c, Å	12.3359(4)	16.2477(3)
α, °	90	103.185(1)
β, °	94.047(1)	103.737(1)
γ, °	90	109.695(1)
V, Å^3	2100.1(1)	2047.23(7)
Z	4	2
ρ_{calcd}, Mg m^{-3}	1.497	1.398
μ (Mo $K\alpha$), mm^{-1}	1.541	1.447
F(000)	932	848
θ range, deg	2.50 to 32.59	2.24 to 31.08
Index ranges	$-30 \le h \le 30$	$-15 \le h \le 15$
	$-11 \le k \le 12$	$-18 \le k \le 18$
	$-18 \le l \le 18$	$-22 \le l \le 22$
No. of reflns collected	49952	249024
Completeness to θ_{max}	99.9%	99.9%
No. indep. Reflns	7460	11948
No. obsd reflns with ($I>2\sigma(I)$)	6753	10468
No. refined params	247	469
GooF (F^2)	1.040	1.034
R_1 (F) ($I > 2\sigma(I)$)	0.0278	0.0170
wR_2 (F^2) (all data)	0.0515	0.0427
Largest diff peak/hole, e Å^{-3}	1.422 / −1.341	0.654 / –0.511
CCDC number	2092933	2092934

Table S5. cont.

**3**	**4**	**5a(5b)**·1/2 toluene
$C_{16}H_{34}B_{11}NOTe$	$C_{19}H_{36}B_{11}N_3Te$	$C_{15.5}H_{30}B_{11}NTe$
502.95	553.02	476.91
Triclinic	Triclinic	Triclinic
0.06 × 0.06 × 0.05	0.04 × 0.04 × 0.04	0.08 × 0.08 × 0.05
$P\bar{1}$	$P\bar{1}$	$P\bar{1}$
10.413(5)	8.5339(3)	10.104(5)
10.519(5)	11.5599(4)	11.010(5)
11.686(5)	14.2790(5)	12.030(5)
99.615(5)	94.760(1)	71.320(5)
93.237(5)	90.566(1)	89.035(5)
99.329(5)	107.719(1)	66.242(5)
1240(1)	1336.27(8)	1150.6(9)
2	2	2
1.346	1.374	1.377
1.207	1.127	1.295
504	556	474
2.42 to 27.57	2.45 to 28.39	2.29 to 27.54
$-13 \le h \le 13$	$-11 \le h \le 9$	$-11 \le h \le 13$
$-13 \le k \le 13$	$-15 \le k \le 15$	$-14 \le k \le 14$
$-15 \le l \le 15$	$-19 \le l \le 19$	$-15 \le l \le 15$
123938	47998	21020
99.8%	99.5%	99.8%
5734	6673	5292
5324	5852	4749
278	316	295
1.072	1.062	1.079
0.0191	0.0247	0.0464
0.0507	0.0530	0.1222
0.835 / –0.560	0.411 / –0.603	1.817 / –2.637
2092935	2092936	2092937

Materials and methods

Computational Methodology. Starting from the available solid-state molecular geometries density functional theory (DFT) computations were performed in the gas-phase at the B3PW91/6-311+G(2df,p)[S3] level of theory using Gaussian09.[S4] For the Te atoms, effective core potentials (ECP28MDF)[S5] and corresponding cc-pVTZ basis set[S5] were utilized. Dispersion was taken account for by the empirical disperison correction of Grimme.[S6] The wavefunction files were used for a topological analysis of the electron density according to the Atoms-In-Molecules space-partitioning scheme[19] using AIM2000,[S7] whereas DGRID[S8] was used to generate and analyze the Electron-Localizability-Indicator (ELI-D)[21] related real-space bonding descriptors applying a grid step size of 0.05 a.u. (0.12 a.u. for visualization). The NCI[20] grids were computed with NCIplot (0.1 a.u. grids).[S9] Bond paths are displayed with AIM2000, ELI-D and NCI figures are displayed with MolIso,[S10] and spin densities are displayed with GaussView. AIM provides a bond paths motif, which resembles and exceeds the Lewis picture of chemical bonding, disclosing all types and strengths of interactions. Additionally, it provides atomic volumes and charges. Analyses of the reduced density gradient, $s(\mathbf{r}) = [1/2(3\pi^2)^{1/3}]|\nabla\rho|/\rho^{4/3}$, according to the NCI method is used to visualize non-covalent bonding aspects. An estimation of different non-covalent contact types according to steric/repulsive ($\lambda_2 > 0$), van der Waals-like ($\lambda_2 \approx 0$), and attractive ($\lambda_2 < 0$) is facilitated by mapping the ED times the sign of the second eigenvalue of the Hessian ($\text{sign}(\lambda_2)\rho$) on the *iso*-surfaces of $s(\mathbf{r})$. AIM and NCI are complemented by the ELI-D, which provides electron populations and volumes of bonding and lone-pair basins and is especially suitable for the analysis of (polar-)covalent bonding aspects.

Real space bond indicator analysis. The electronic rearrangements *via* the Umpolung were monitored by means of density functional theory (DFT) calculations of compounds **1**, **2a**, **3**, **4**, **5a**, and **6a**, followed by a comprehensive study of a set of real-space bonding indicators (RSBI) derived from the Atoms-In-Molecules (AIM),[19] noncovalent interactions index (NCI),[20] and electron localizability indicator (ELI-D)[21] tools. RSBI provide topological, surface, as well as integrated atomic and bonding properties, which together are capable to disclose tiny electronic rearrangements between related structural motifs, which typically are not straight-forwardly accessible by orbital-based approaches. AIM provides a bond paths motif of all atom-atom contacts, including weak secondary intramolecular interactions, NCI uncovers regions in space in which noncovalent bonding aspects prevail, thereby complementing ELI-D, which is especially useful to analyze (polar) covalent bonds. The idiosyncratic N–Te···H–B to N–H···Te–B transformation is considered in more detail in that the transition state (TS) between compounds **1** and **5a** was calculated using the QST3 algorithm[S11] of the Gaussian16 software suite.[S12] AIM bond topology and NCI and ELI-D *iso*-surfaces of DFT models **1**, **TS**, **5** and **2** are given in Figures **S52-S58** (see supporting information for **3**, **4**, and **6**), quantitative results of all models are collected in Tables **S6-S9**, focusing on all types of Te–E (E = Te, O, N, C, B, H) contacts. The AIM bond paths motif of **1** shows primary Te-N/C as well as secondary Te/H$^{\delta+}\cdots$H$^{\delta-}$ contacts, see Figure **S52a**, the latter of which are dominated by noncovalent bonding aspects, see blue and green colored NCI basins between Te or (C)H on the one side and H(B) on the other in Figure **S52b**. The ELI-D *iso*-surface shows a compression of the basins representing the hydridic H(B) atoms being close to the Te atom, see Figure **S52c**, which is due to the steric demands of the Te atoms lone pairs. Mapping the ELI-D distribution on the basin-surface of the one H(B) forming the Te $^{\delta+}\cdots$H$^{\delta-}$ contact unravels the onset of covalent contributions to an otherwise rather ionic contact by forming a small excrescence in direction towards the Te atom on the basically flat basin surface, see Figure **S52d**. DFT models **5a** and **2a** also contain Te–B and/or

Te–Te bonds. Notably, the quite weak Te–Te contact includes both noncovalent (blue NCI basin, see Figure **S56b**) and covalent (strongly deformed ELI-D basin, see Figure **S56d**) bonding aspects, whereas Te–B bonds are rather covalent. These observations are supported by the corresponding quantitative RSBI, see Tables **S6** and **S7**. The Te–C bond properties in all compounds are virtually identical and were thus averaged, see Table **S8** for full list. The Te–B, Te–C, and short Te–N (Te–N < 2.5 Å) bonds span the range for typical polar-covalent (including dative) bonds with low electron densities (ED, ρ(**r**)) between 0.5 and 1 $eÅ^{-3}$, the Laplacian of the ED ($\nabla^2\rho(\mathbf{r})$) being positive or negative, but close to zero, and pronounced kinetic and total energy density over ED ratios (G/ρ(**r**) and H/ρ(**r**)). In that series, the relative bonding aspects change from mainly covalent (Te–B) to polar-covalent (Te–C) to dative (Te–N). Correspondingly, the Raub-Jansen Index,[S13] which is a measure for bond polarities, is about 70% for the less polar Te–B and Te–C bonds, but as high as 95% for the dative Te–N bonds. Finally, bond characteristics of weak coordination bonds are obtained for the long-distant Te–N (larger than 2.5 Å) and Te–O contacts, and also for the weak Te–Te bond, with ED values lower than 0.5 $eÅ^{-3}$ and specifically less negative or even positive H/ρ(**r**) values, indicating decreasing covalency, i.e. electron sharing. The transition state between DFT model compounds **1** and **5a** was modelled under the assumption that the hydridic H(B) atom, which is in contact to the Te atom and opposing the N atom of the aryltellurenyl cation in **1** is *not* the one becoming the protic H(N) atom in **5a**, but rather serves as a linker between the cation and the anion. This is relevant, because due to the steric demands and electronic impact of the Te atoms lone pairs, only the site opposite to the N atom may be attacked by a nucleophile, see Figure **S52**. The carborane may then rotate sidewise so that the B–H bond of the adjacent H atom (the one which becomes protic) is exposed to both the N and the Te atoms electronic influence, see Figure **S53**, resulting in a N···H(Te)B and finally N–H···Te–B arrangement, see Figure **S54**. Supported by the N atoms lone pair, the Te atom inserts into the H–B bond. The **TS** is 118.1 kJ mol^{-1} higher in energy than **1** and 193.2 kJ mol^{-1} higher in

energy than **5a**, resulting in an overall energy gain of about 75 kJ/mol for the **1** to **5a** transformation. In contrast to the noncovalent $Te^{\delta+}\cdots H^{\delta-}$ contact in **1** (visible as blue coloured disc-shaped NCI basin, *vide supra*), the H(Te)B contact in the **TS** is indeed considerably covalent (red coloured ring-shaped NCI basin), compare Figures **S52b** and **S53b**. This is also reflected in the bond topology with an ED value of 0.59 $eÅ^{-3}$ and a considerably negative H/ρ(**r**) value of –0.38 a.u. for the Te–H(B) contact in the **TS**, compared to 0.34 $eÅ^{-3}$ and –0.21 a.u. in **1**, see Table **7**. In **5a**, the Te···H contact shows electronic characteristics of weak coordination bonds (ρ(**r**) = 0.23 , H/ρ(**r**) = –0.14 a.u.), see also Figure **S54b**. The RJI provides a measure how the electron population within an ELI-D basin is distributed over all AIM atoms overlapping with that basin, in other words sharing it. Typically, this refers to only two binding atoms and thus the RJI is used for estimation of the bond polarity. In our analysis of the **TS** the RJI is used in a somewhat different fashion as we display the contributions of the Te, H, B, and N atoms to the H atoms ELI-D basin in all three DFT models, see Tables B. In **1**, 80 % of the ED within the hydridic H ELI-D basin are located within the H atomic AIM basin, 16 % in the B basin, and 4 % in the Te basin, according for the weak $Te^{\delta+}\cdots H^{\delta}$ contact. In the **TS**, however, the contribution of the AIM H atomic basin has dropped down to 53 %, whereas the B and Te contributions rise to 22 and 26 %, in accordance with a H(Te)B arrangement. The N atom is not involved so far by means of RJI. In the product **5a**, only the N (77 %) and H (23 %) atoms are involved into the now protic ELI-D basin of the H atom. The AIM atomic and fragmental charges are given in Table **S8**. About 0.2 e are transferred from the carborate anion in **1** to the tellurenyl cation. This is increased to about 0.6 e in the **TS**. The THF (**3**) or DMAP (**4**) adducts show smaller values of 0.1 or 0.2 e, respectively. About 0.4 e are transferred to the tellurenyl cation from the 12-[2-(*t*BuN{H}CH)C_6H_4Te fragment in **2a**.

Table S6. Topological and integrated bond properties from AIM and ELI-D.

model	contact or basin	d [Å]	d_1 [Å]	d_2 [Å]	ρ(r) [eÅ^{-3}]	∇^2ρ(r) [eÅ^{-5}]	ε
**2a**	Te-Te	2.998	1.422	1.577	0.31	0.6	0.18
**3**	Te-N	2.135	1.058	1.078	0.69	4.2	0.24
**1**	Te-N	2.163	1.072	1.091	0.65	4.1	0.25
**2a**	Te1-N	2.223	1.109	1.114	0.58	3.4	0.23
**4**	Te-N	2.228	1.109	1.120	0.57	3.5	0.23
**4**	Te-Np	2.307	1.155	1.151	0.49	3.0	0.06
**6a**	Te-N	2.928	1.560	1.373	0.15	1.4	0.16
**TS**	Te-N	3.075	1.685	1.401	0.13	1.2	0.94
**6a**	Te-B	2.228	1.291	0.939	0.69	-2.8	0.06
**2a**	Te2-B	2.229	1.375	0.854	0.65	-3.1	0.04
**5a**	Te-B	2.232	1.343	0.890	0.65	-2.8	0.06
**3**	Te-O	2.423	1.260	1.165	0.33	2.9	0.05
**3**	LP(O)						
all-8	*Te-C*	*2.102*	*1.075*	*1.027*	*0.85*	*0.0*	*0.18*

model	contact or basin	G/ρ(r) [a.u.]	H/ρ(r) [a.u.]	N_{ELI} [e]	V_{ELI} [Å^3]	γ_{ELI}	RJI
**2a**	Te-Te	0.35	-0.21	1.83	6.1	1.64	88.1
**3**	Te-N	0.84	-0.41	2.48	5.1	1.67	92.1
**1**	Te-N	0.83	-0.39	2.55	5.5	1.68	93.5
**2a**	Te1-N	0.75	-0.35	2.47	5.4	1.70	94.3
**4**	Te-N	0.77	-0.34	2.51	5.6	1.72	94.5
**4**	Te-Np	0.71	-0.29	2.82	7.8	1.76	96.3
**6a**	Te-N	0.63	0.03	2.64	8.4	1.94	99.8
**TS**	Te-N	0.59	0.03	2.57	7.5	1.97	99.8
**6a**	Te-B	0.21	-0.49	1.86	6.4	1.74	56.1
**2a**	Te2-B	0.21	-0.54	1.83	6.1	1.64	68.5
**5a**	Te-B	0.21	-0.51	1.68	5.4	1.64	63.6
**3**	Te-O	0.75	-0.12	2.32	4.0	1.67	99.8
**3**	LP(O)			2.65	5.7	1.75	99.9
all-8	*Te-C*	*0.54*	*-0.54*	*2.01*	*5.2*	*1.68*	*72.1*

For all bonds, d_1 and d_2 are the distance between the atom and the bcp, $\rho(\mathbf{r})_{bcp}$ is the electron density at the bcp, $\nabla^2\rho(\mathbf{r})_{bcp}$ is the corresponding Laplacian, ε is the bond ellipticity, $G/\rho(\mathbf{r})_{bcp}$ and $H/\rho(\mathbf{r})_{bcp}$ are the kinetic and total energy density over $\rho(\mathbf{r})_{bcp}$ ratios, N_{ELI} and V_{ELI} are electron populations and volumes of related ELI-D basins, γ_{ELI} is the ELI-D value at the attractor position, RJI is the Raub-Jansen Index.

Table S7. Topological and integrated bond properties from AIM and ELI-D for the Te–H interaction in DFT models **1**, **TS**, and **5a**.

model	contact or basin	d [Å]	d_1 [Å]	d_2 [Å]	$d_1 + d_2$ [Å]	ρ(r) [eÅ^{-3}]	∇^2ρ(r) [eÅ^{-5}]	G/ρ(r) [a.u.]	H/ρ(r) [a.u.]
**1**	Te–H	2.115	1.289	0.835	2.124	0.34	1.5	0.52	-0.21
**TS**	Te–H	1.932	1.200	0.781	1.980	0.59	0.3	0.42	-0.38
**5a**	Te–H	2.334	1.613	0.722	2.334	0.23	1.0	0.42	-0.14

model	contact or basin	ε	N_{ELI} [e]	V_{ELI} [Å^3]	γ_{ELI}	RJI (Te)	RJI (H)	RJI (B)	RJI (N)
**1**	Te–H	0.23	1.95	9.4	6.60	3.8	79.9	16.2	0.0
**TS**	Te–H	1.58	2.27	7.6	4.27	25.7	52.7	21.6	0.0
**5a**	Te–H	0.02	2.26	6.0	3.84	0.0	22.9	0.0	77.1

For all bonds, d_1 and d_2 are the distance between the atom and the bcp, $\rho(\mathbf{r})_{bcp}$ is the electron density at the bcp, $\nabla^2\rho(\mathbf{r})_{bcp}$ is the corresponding Laplacian, ε is the bond ellipticity, $G/\rho(\mathbf{r})_{bcp}$ and $H/\rho(\mathbf{r})_{bcp}$ are the kinetic and total energy density over $\rho(\mathbf{r})_{bcp}$ ratios, N_{ELI} and V_{ELI} are electron populations and volumes of related ELI-D basins, γ_{ELI} is the ELI-D value at the attractor position, RJI is the Raub-Jansen Index.

Table S8. AIM charges (in e) of relevant atoms and molecular fragments for the DFT models.

	**1**	**2a**	**3**	**4**	**5a**	**6a**	**TS**
$CB_{11}H_{12}^{(2-)}$	-0.79						-0.38
$CB_{11}H_{11}^{(1-)}$		-0.50			-0.59	-0.80	
$TeN^{(+)}$	0.79	0.64	0.91	0.79		-0.20	0.38
$TeNH^{(2+)}$		0.86			0.60		
THF/Npyr			0.10	0.22			
sum	0.00	1.00	1.00	1.00	0.04	-1.00	0.00

Table S9. Topological and integrated bond properties from AIM and ELI-D for the Te–C interaction in the DFT models.

model	contact or basin	d [Å]	d_1 [Å]	d_2 [Å]	ρ(r) [eÅ^{-3}]	∇^2ρ(r) [eÅ^{-5}]	ε
**1**	Te–C	2.074	1.065	1.009	0.90	–0.1	0.27
**3**	Te–C	2.076	1.069	1.006	0.90	–0.4	0.27
**4**	Te–C	2.089	1.073	1.016	0.88	–0.4	0.28
**2a**	Te1–C	2.094	1.072	1.023	0.86	0.0	0.26
**6a**	Te–C	2.109	1.072	1.037	0.83	0.5	0.21
**TS**	Te–C	2.114	1.088	1.025	0.83	–0.4	0.06
**5a**	Te–C	2.129	1.078	1.051	0.79	0.6	0.04
**2a**	Te2–C	2.130	1.085	1.044	0.81	0.1	0.05
All	*Te–C*	*2.102*	*1.075*	*1.027*	*0.85*	*0.0*	*0.18*

model	contact or basin	G/ρ(r) [a.u.]	H/ρ(r) [a.u.]	N_{ELI} [e]	V_{ELI} [Å^3]	γ_{ELI}	RJI
**1**	Te–C	0.55	–0.56	2.07	5.3	1.66	70.5
**3**	Te–C	0.53	–0.56	2.02	5.1	1.65	69.4
**4**	Te–C	0.53	–0.55	2.02	5.1	1.67	70.3
**2a**	Te1–C	0.55	–0.55	2.05	5.3	1.67	71.8
**6a**	Te–C	0.57	–0.53	2.02	5.3	1.69	74.9
**TS**	Te–C	0.50	–0.53	1.96	5.2	1.67	70.0
**5a**	Te–C	0.57	–0.51	1.96	5.3	1.72	76.5
**2a**	Te2–C	0.53	–0.52	1.97	5.2	1.70	73.0
all	*Te–C*	*0.54*	*–0.54*	*2.01*	*5.2*	*1.68*	*72.1*

For all bonds, d_1 and d_2 are the distance between the atom and the bcp, $\rho(\mathbf{r})_{bcp}$ is the electron density at the bcp, $\nabla^2\rho(\mathbf{r})_{bcp}$ is the corresponding Laplacian, ε is the bond ellipticity, $G/\rho(\mathbf{r})_{bcp}$ and $H/\rho(\mathbf{r})_{bcp}$ are the kinetic and total energy density over $\rho(\mathbf{r})_{bcp}$ ratios, N_{ELI} and V_{ELI} are electron populations and volumes of related ELI-D basins, γ_{ELI} is the ELI-D value at the attractor position, RJI is the Raub-Jansen Index.

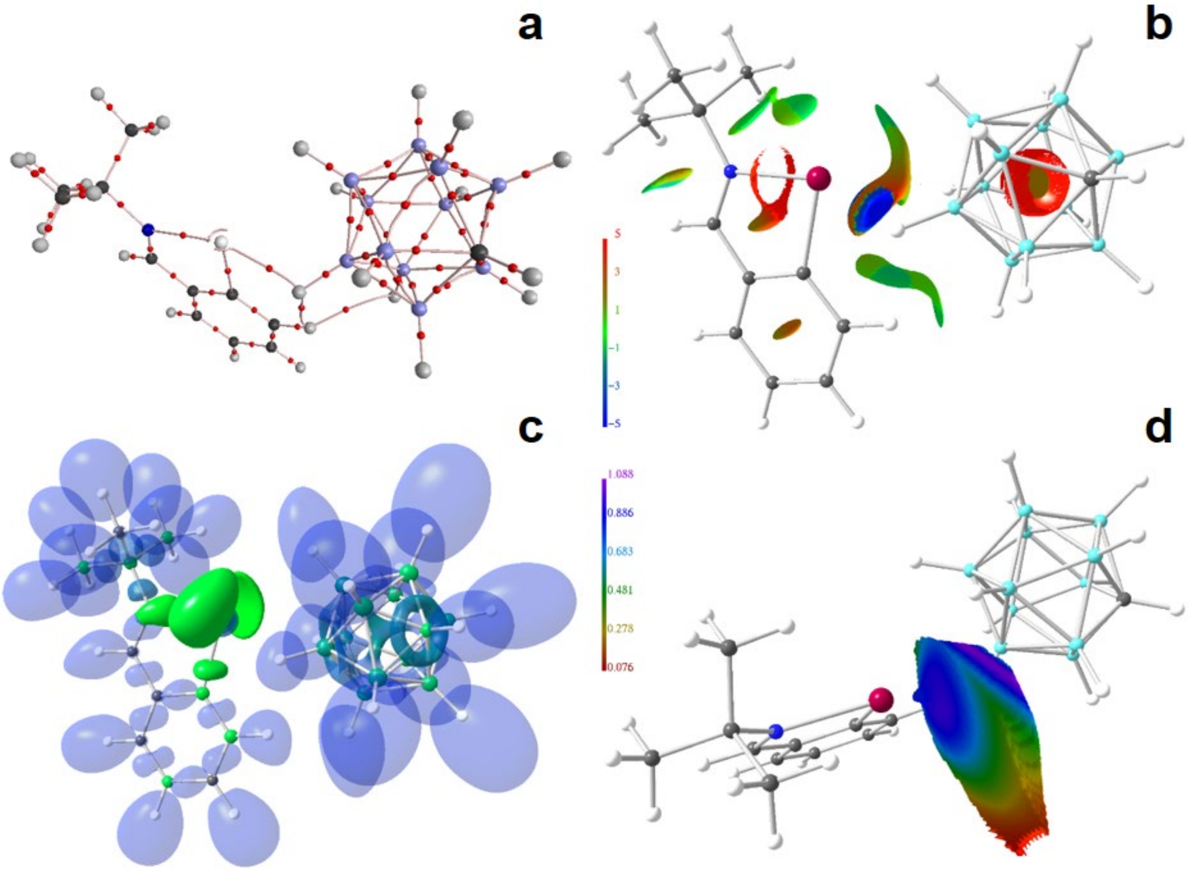

Figure S52. RSBI analysis of **1** (a) AIM bond paths motif, (b) NCI *iso*-surface at s(**r**) = 0.5, (c) ELI-D localization domain representation at *iso*-value of 1.4, (d) ELI-D distribution mapped on the H(B) ELI-D basin.

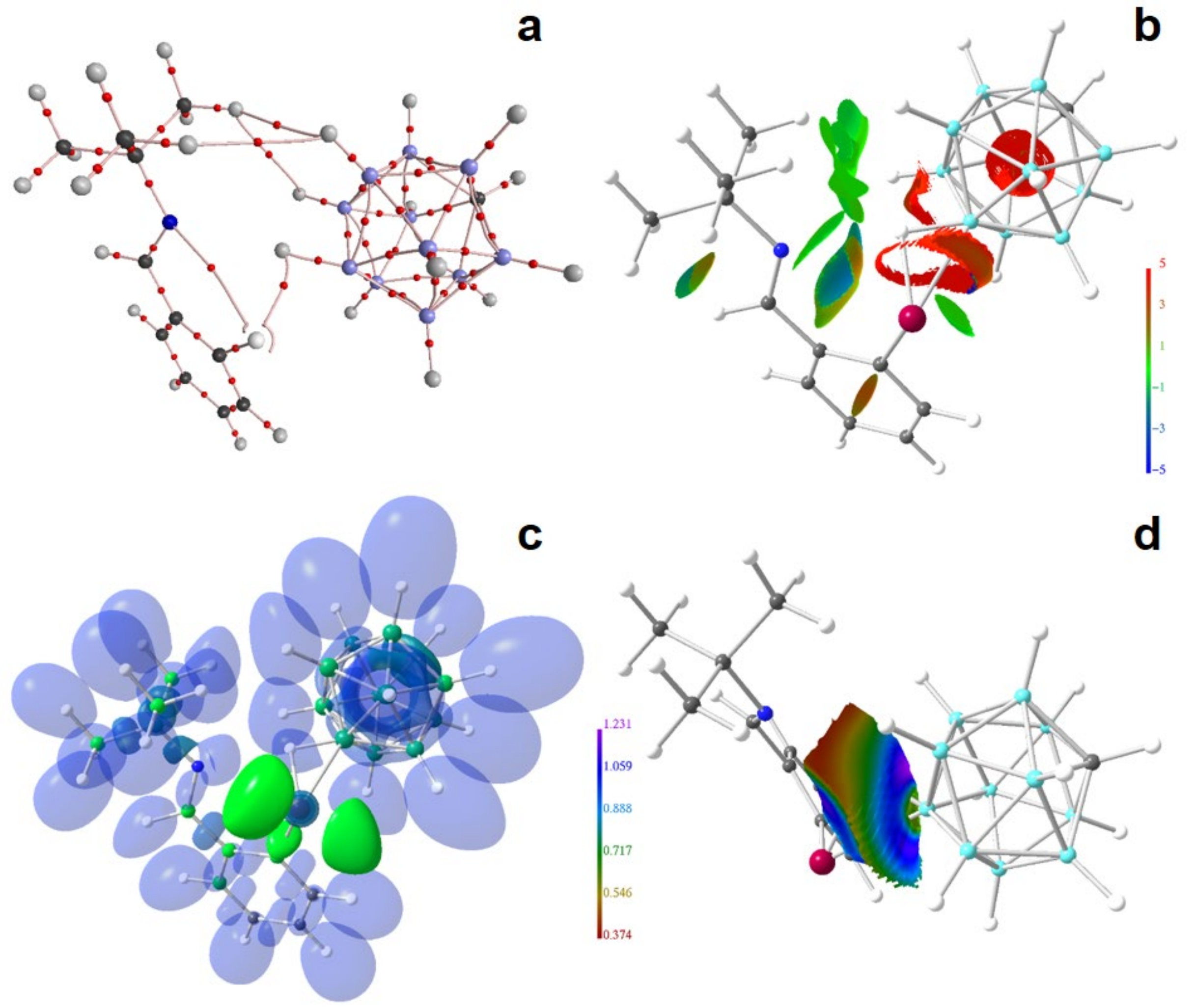

Figure S53. RSBI analysis of **TS** (a) AIM bond paths motif, (b) NCI *iso*-surface at s(**r**) = 0.5, (c) ELI-D localization domain representation at *iso*-value of 1.4, (d) ELI-D distribution mapped on the H(B) ELI-D basin.

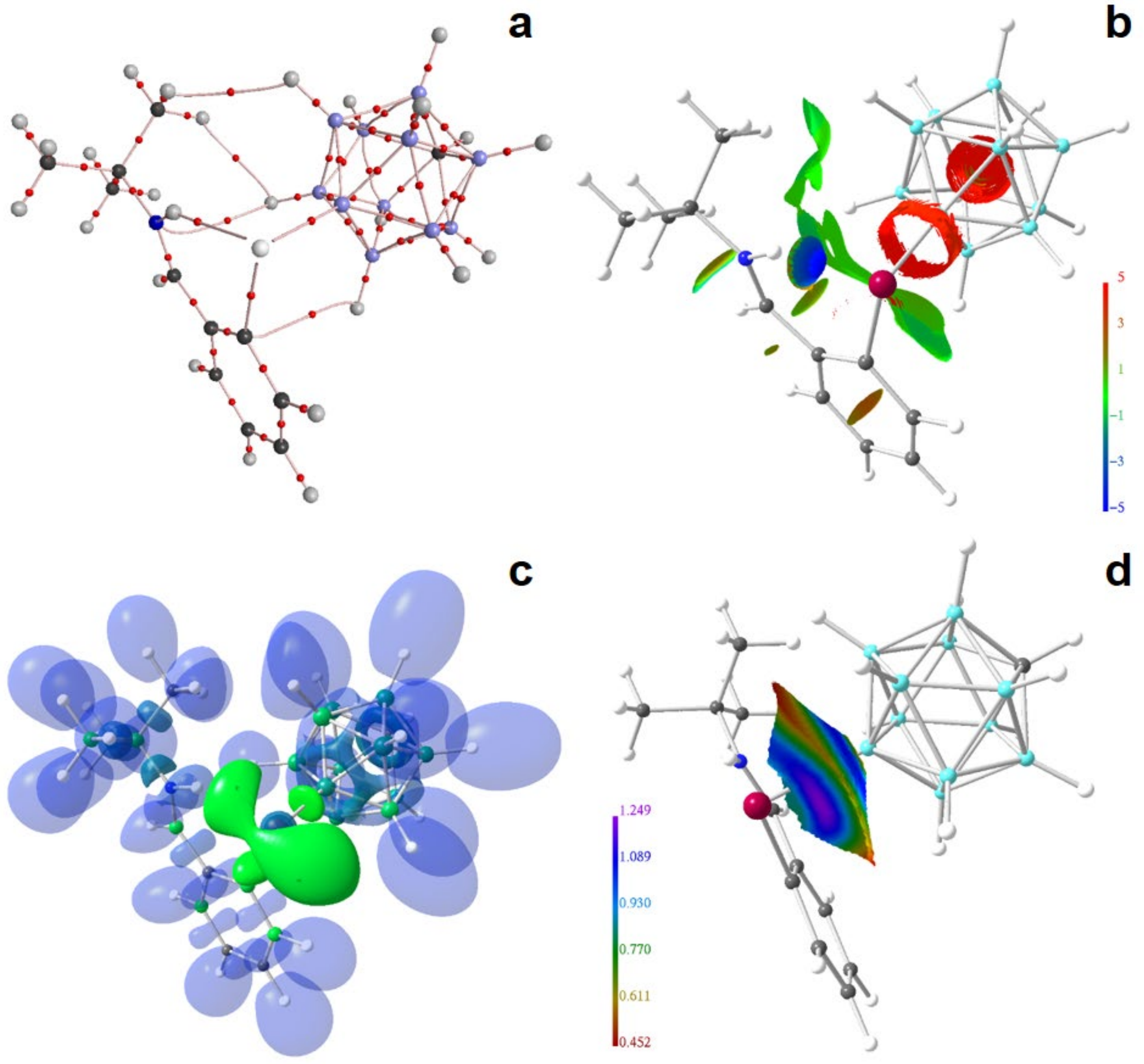

Figure S54. RSBI analysis of **5a** (a) AIM bond paths motif, (b) NCI *iso*-surface at s(**r**) = 0.5, (c) ELI-D localization domain representation at *iso*-value of 1.4, (d) ELI-D distribution mapped on the Te–B ELI-D basin.

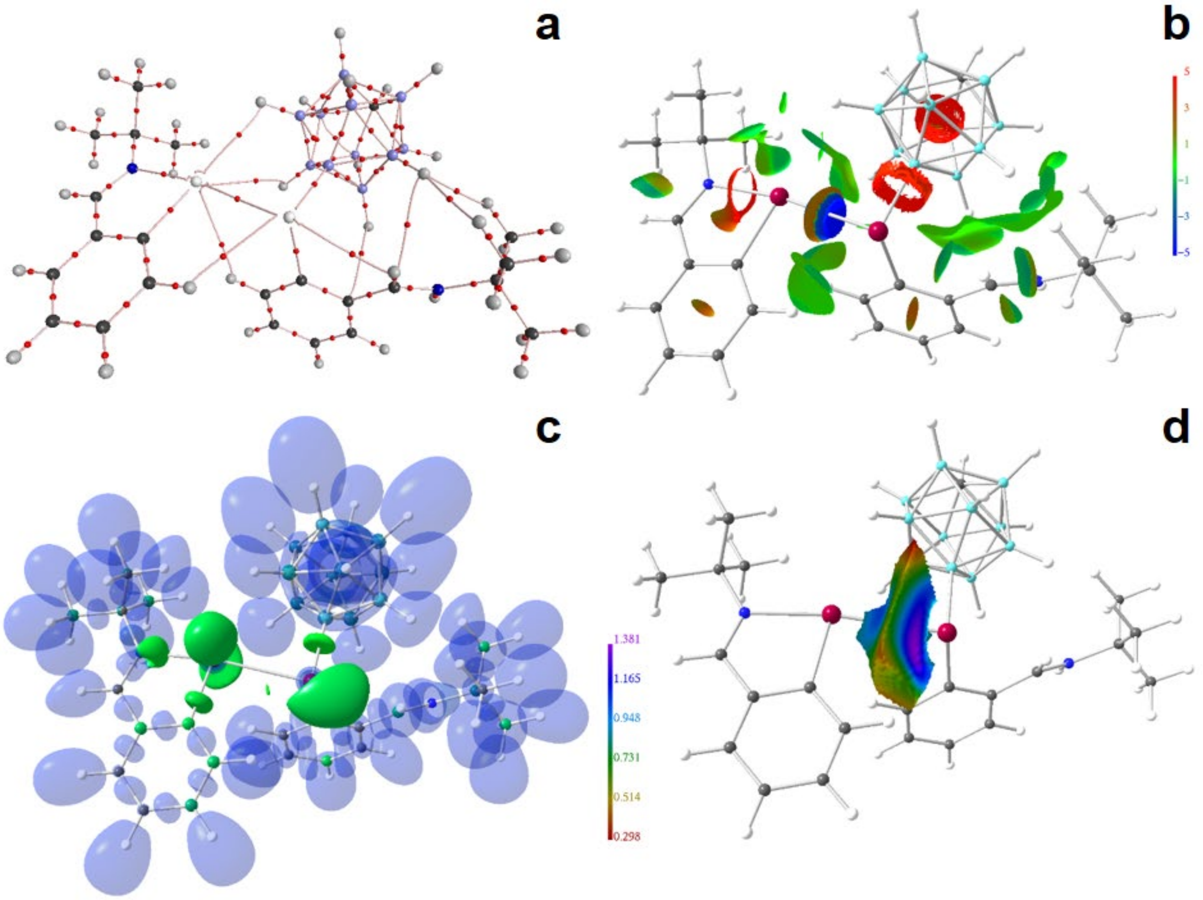

Figure S55. RSBI analysis of **2a** (a) AIM bond paths motif, (b) NCI *iso*-surface at s(**r**) = 0.5, (c) ELI-D localization domain representation at *iso*-value of 1.4, (d) ELI-D distribution mapped on the Te–Te ELI-D basin.

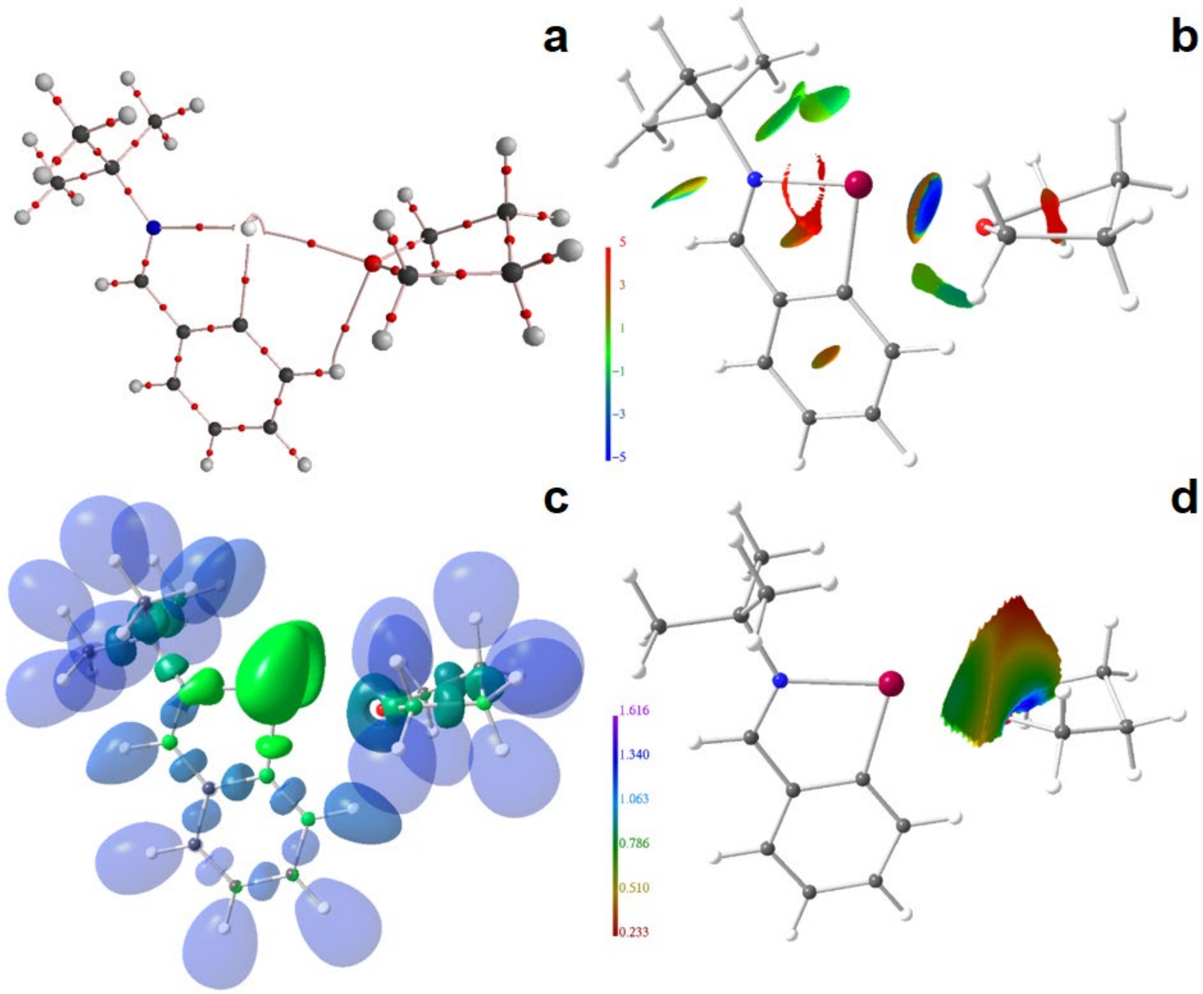

Figure S56. RSBI analysis of **3** (a) AIM bond paths motif, (b) NCI *iso*-surface at s(**r**) = 0.5, (c) ELI-D localization domain representation at *iso*-value of 1.4, (d) ELI-D distribution mapped on the (LP)O ELI-D basin.

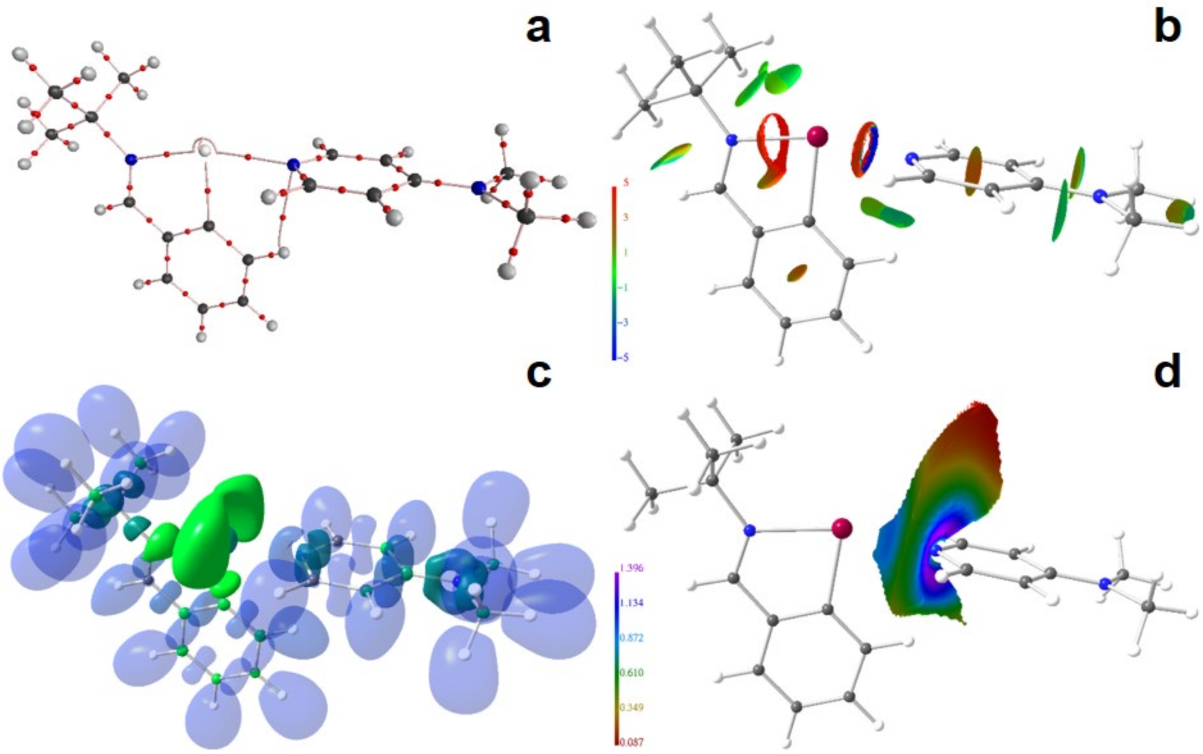

Figure S57. RSBI analysis of **4** (a) AIM bond paths motif, (b) NCI *iso*-surface at s(**r**) = 0.5, (c) ELI-D localization domain representation at *iso*-value of 1.4, (d) ELI-D distribution mapped on the (LP)N ELI-D basin.

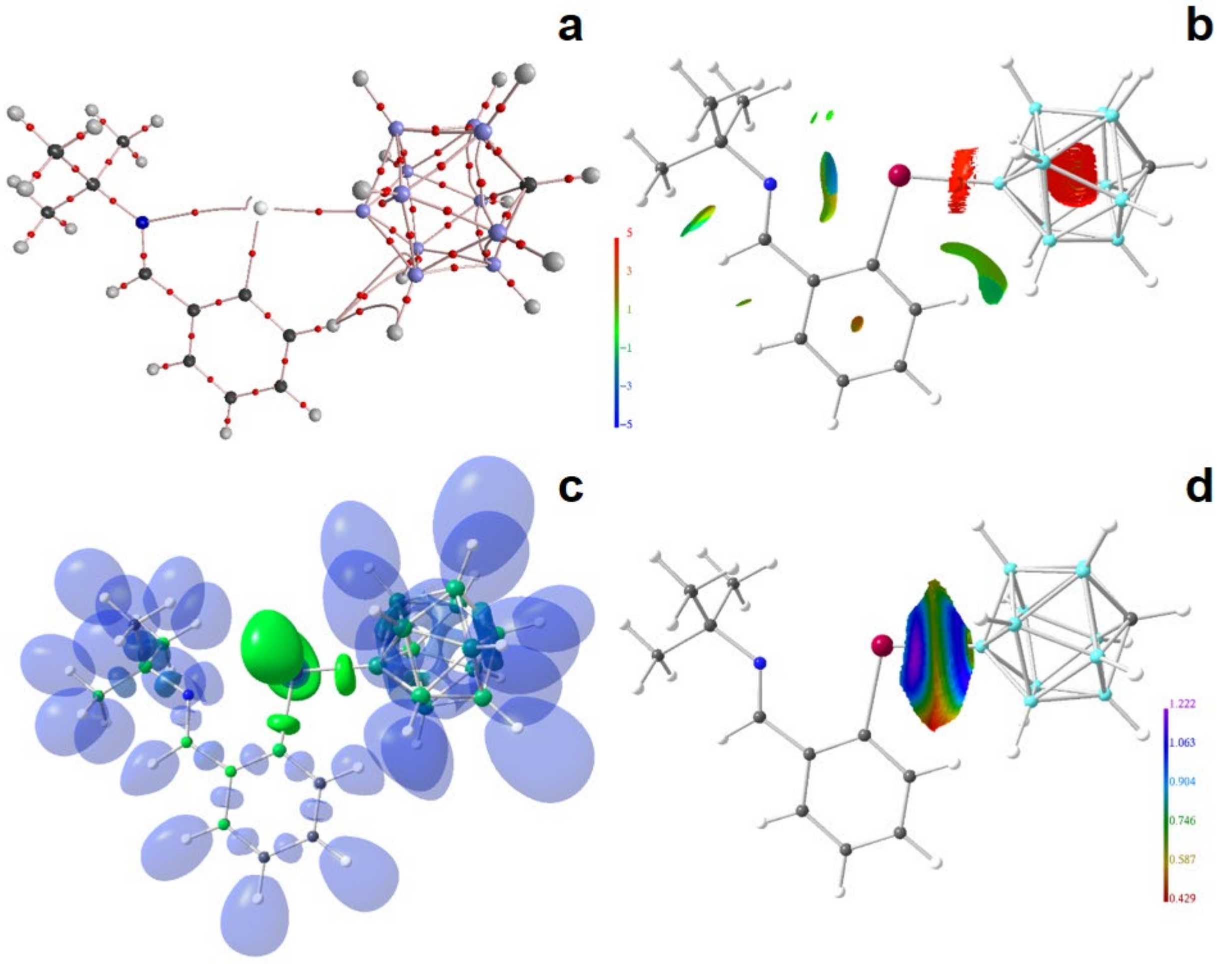

Figure S58. RSBI analysis of **6a** (a) AIM bond paths motif, (b) NCI *iso*-surface at s(**r**) = 0.5, (c) ELI-D localization domain representation at *iso*-value of 1.4, (d) ELI-D distribution mapped on the Te–B ELI-D basin.

pK_a values calculations

The $pKa_{(MeCN)}$ values were calculated using the SMD continuum solvation model.[S14] We chose the M052X/6-31+G** level of theory listed among those taken for the optimization of the SMD parameters.[S14] For Te atoms, the Def2SVP basis set with the ECP-28 pseudopotential was used. [S15] DFT calculations were performed for $[CB_{11}H_{12}]^{-}$ anion **1**, **1′**, **5a** and their conjugate bases. Analogously, 17 tabulated organic bases and their conjugated acids[S16] and 8 tabulated anions and dianions of organic diacids[S17] were calculated (Tables S10, S11). The acid and conjugate base structures were optimized and the vibrational frequencies were computed in MeCN solution with the Gaussian 09 package.[S18] The $pKa_{(MeCN)}$ values were then determined as $pKa_{(MeCN)} = \Delta_{diss}G^{\ominus}{}_{(MeCN)}/ 2.303RT$ (Tables S10, S11). The $\Delta_{solv}G^{\ominus}$ (H^{+}) = –251.9 kcal mol^{-1} proton solvation free energy was used.[S19] The 1.9 kcal mol^{-1} correction[S20] for the standard state change on going from the gas to solution phase was taken into account. The theoretical pKa's correlate well with the experimental data (Tables S10, S11). The experimental pKa_{exp} values were plotted against calculated pKa_{calc} (Tables S10, S11). Then the expected pKa magnitudes were determined on the basis of linear regression analysis (Tables S10, S11). The mean absolute error (MAE) determined from the differences between the experimental and predicted pKa values decreases from 2.4 to 0.6 on going from $pKa_{calc(MeCN)}$ to $pKa_{cor(MeCN)}$. The detailed procedure for pKa determination was described previously.[18]

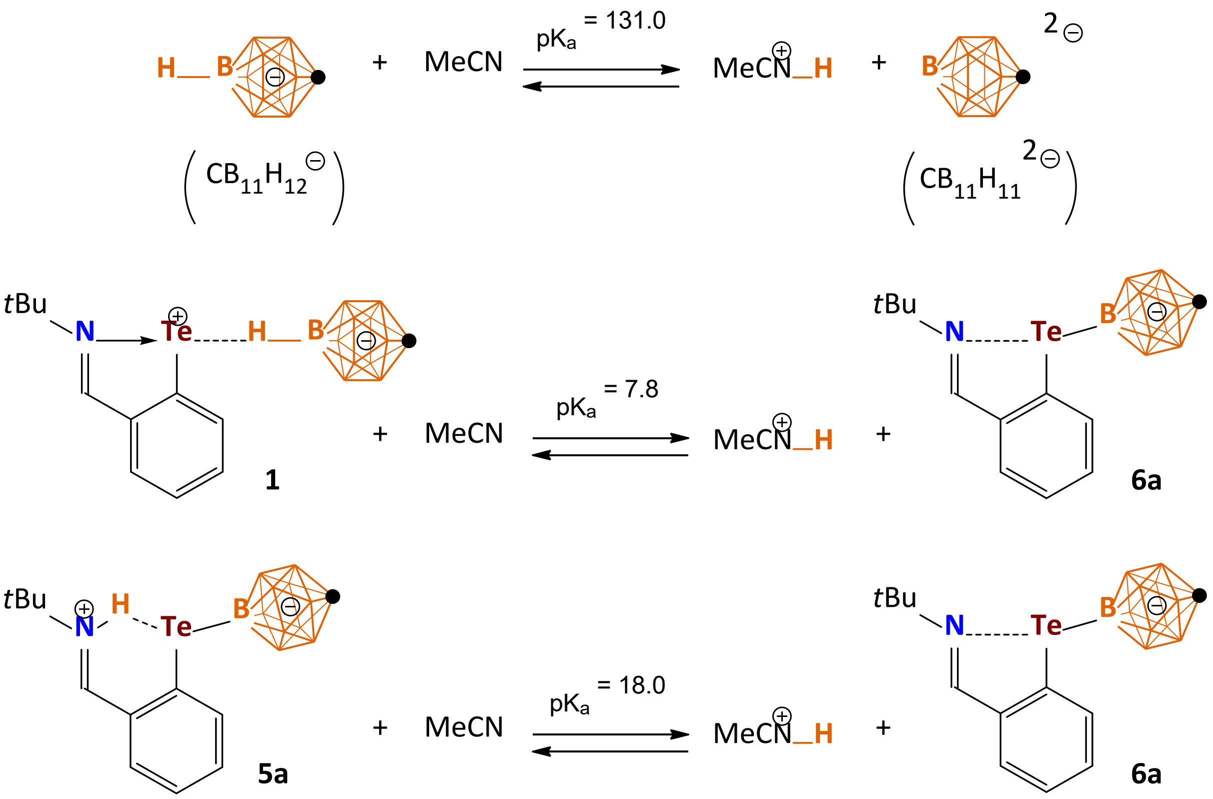

Figure S59. Acid-base reactions to calculate the pK_a values

Table S10. Calculated pK_a in acetonitrile

Compound	"absolute" pK_a*	pK_a from linear correlation**
$[CB_{11}H_{12}]^-$	182.4	131.0
**1**	6.02	7.42
**1′**	5.80	7.21
**5a**	18.97	17.14

* Calculated against experimental proton affinity of acetonitrile

** Linear correlation of experimental *vs*. calculated pK_a of cationic and anionic acids, respectively, for pK_a determination of cationic (**1**, **4**) and anionic $[B_{11}CH_{12}]^-$ compounds.

Table S11. Experimental[S16] pKa_{exp}, calculated pKa_{calc} and expected on the basis of correlation analysis pKa_{cor} values for conjugated acids of organic bases in acetonitrile. The differences between the experimental and theoretical $pKa_{(MeCN)}$ values are given.

Base	pKa_{exp}	pKa_{calc}	$pKa_{exp} – pKa_{calc}$	pKa_{cor}	$pKa_{exp} – pKa_{cor}$
Pyridine	12.5	13.9	-1.4	13.2	-0.8
2-Methoxypyridine	10.6	12.4	-1.8	12.1	-1.5
2-Amionbenzimidazole	16.0	16.4	-0.5	15.2	0.7
2-Nitroaniline	5.0	1.6	3.5	3.9	1.2
3-Chloropyridine	10.1	10.4	-0.3	10.6	-0.5
3-Nitroaniline	7.7	6.5	1.2	7.6	0.1
4-(Trifluoromethyl)anilne	8.2	6.5	1.7	7.6	0.5
4-Nitroaniline	6.2	3.9	2.3	5.7	0.5
2,4,6-Trimethylpyridine	14.4	16.1	-1.7	15.0	-0.6
Aniline	10.6	9.8	0.8	10.2	0.4
Benzylamine	16.8	18.0	-1.2	16.4	0.4
1,8-Diazabicyclo[5.4.0]undec-7-ene	24.1	27.0	-2.9	23.3	0.9
4-Dimethylaminopyridine	18.2	18.8	-0.6	17.0	1.2
Dimethylaniline	12.3	14.2	-1.9	13.5	-1.2
Methyldiphenylphosphine	9.6	11.1	-1.5	11.2	-1.6
Pyrrolidine	19.6	22.2	-2.6	19.6	0.0
4-Bromoaniline	9.4	8.4	0.9	9.1	0.3
$CB_{11}H_{11}TeC_6H_4CHN\textit{t}Bu$	–	19.0	–	17.1	–

Table S12. Experimental[S17] pKa_{exp}, calculated pKa_{calc} and expected on the basis of correlation analysis pKa_{cor} values for second step deprotonation of organic diacids in acetonitrile. The differences between the experimental and theoretical $pKa_{(MeCN)}$ values are given.

Acid	pKa_{exp}	pKa_{calc}	$pKa_{exp} – pKa_{calc}$	pKa_{cor}	$pKa_{exp} – pKa_{cor}$
Oxalic acid	27.7	30.1	-2.4	26.2	1.5
Malonic acid	30.5	36.4	-5.9	30.5	0.0
Succinic acid	29.0	34.9	-5.9	29.6	-0.6
Fumaric acid	22.9	25.0	-2.1	22.7	0.2
Phthalic acid	29.8	34.3	-4.5	29.1	0.7
Azelaic acid	24.8	28.5	-3.7	25.1	-0.3
Adipic acid	26.9	31.4	-4.5	27.1	-0.2
Glutaric acid	28.0	32.2	-4.2	27.6	0.3
$H[CB_{11}H_{12}]$	–	182.4	–	131.0	–

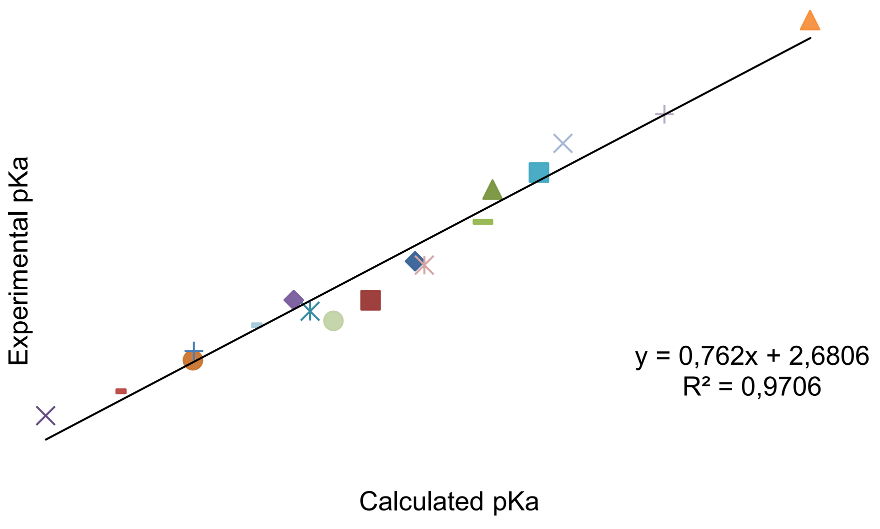

Figure S52. Linear regression between experimental[S16] and calculated pK_a values for conjugated acids of neutral bases in MeCN solution.

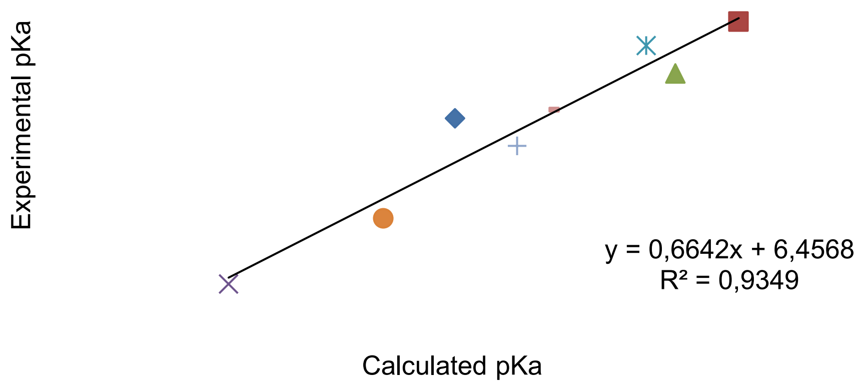

Figure S53. Linear regression between experimental[S17] and calculated pKa_2 values for diacids in MeCN solution

Additional references

[S1] L. J. Farrugia, *J. Appl. Crystal*. **1999**, *32*, 837-838.

[S2] Brandenburg, K. DIAMOND version 3.2i, Crystal Impact GbR, Bonn Germany **2012**.

[S3] (a) Becke, A. D. A New Mixing of Hartree-Fock and Local-Density-Functional Theories. *J. Chem. Phys*. **1993**, *98*, 5648-5652. (b) Perdew, J. P.; Chevary, J. A.; Vosko, S. H.; Jackson, K. A.; Pederson, M. R.; Singh, D. J.; Fiolhais, C. Atoms, Molecules, Solids, and Surfaces: Applications of the Generalized Gradient Approximation for Exchange and Correlation. *Phys. Rev*. B **1992**, *46*, 6671-6687.

[S4] Gaussian 16, Revision C.01, Frisch, M. J.; Trucks, G. W.; Schlegel, H. B.; Scuseria, G. E.; Robb, M. A.; Cheeseman, J. R.; Scalmani, G.; Barone, V.; Petersson, G. A.; Nakatsuji, H.; Li, X.; Caricato, M.; Marenich, A. V.; Bloino, J.; Janesko, B. G.; Gomperts, R.; Mennucci, B.; Hratchian, H. P.; Ortiz, J. V.; Izmaylov, A. F.; Sonnenberg, J. L.; Williams-Young, D.; Ding, F.; Lipparini, F.; Egidi, F.; Goings, J.; Peng, B.; Petrone, A.; Henderson, T.; Ranasinghe, D.; Zakrzewski, V. G.; Gao, J.; Rega, N.; Zheng, G.; Liang, W.; Hada, M.; Ehara, M.; Toyota, K.; Fukuda, R.; Hasegawa, J.; Ishida, M.; Nakajima, T.; Honda, Y.; Kitao, O.; Nakai, H.; Vreven, T.; Throssell, K.; Montgomery, J. A., Jr.; Peralta, J. E.; Ogliaro, F.; Bearpark, M. J.; Heyd, J. J.; Brothers, E. N.; Kudin, K. N.; Staroverov, V. N.; Keith, T. A.; Kobayashi, R.; Normand, J.; Raghavachari, K.; Rendell, A. P.; Burant, J. C.; Iyengar, S. S.; Tomasi, J.; Cossi, M.; Millam, J. M.; Klene, M.; Adamo, C.; Cammi, R.; Ochterski, J. W.; Martin, R. L.; Morokuma, K.; Farkas, O.; Foresman, J. B.; Fox, D. J. Gaussian, Inc., Wallingford CT, 2016.

[S5] (a) Peterson, K. A. Systematically convergent basis sets with relativistic pseudopotentials. I. Correlation consistent basis sets for the post-d group 13–15 elements *J. Chem. Phys.* **2003**, *119*, 11099. (b) Peterson, K. A.; Figgen, D.; Goll, E.; Stoll, H.; Dolg, M.: Systematically convergent basis sets for transition metals. II.

Pseudopotential-based correlation consistent basis sets for the group 11 (Cu, Ag, Au) and 12 (Zn, Cd, Hg) elements. *J. Chem. Phys*. **2003**, *119*, 11113-11123.

[S6] Grimme, S.; Anthony, J.; Ehrlich, S.; Krieg, H., A consistent and accurate ab initio parametrization of density functional dispersion correction (DFT-D) for the 94 elements H-Pu. J. Chem. Phys. 2010, 132, 154104.

[S7] Biegler-König, F.; Schönbohm, J.; Bayles, D. A Program to Analyze and Visualize Atoms in Molecules. *J. Comput. Chem*. **2001**, *22*, 545-559.

[S8] Kohout, M. *DGRID*-4.6 Radebeul, **2015**.

[S9] Contreras-García, J.; Johnson, E.; Keinan, S.; Chaudret, R.; Piquemal, J.-P.; Beratan, D.; Yang, W. NCIPLOT: A Program for Plotting Noncovalent Interaction Regions. *J. Chem. Theor. Comp.* **2011**, *7*, 625-632.

[S10] Hübschle, C. B.; Luger, P. MolIso – A Program for Colour-Mapped Iso-Surfaces. *J. Appl. Crystallogr*. **2006**, *39*, 901-904.

[S11] C. Peng; H. B. Schlegel, *Israel J. Chem.* **1993**, *33*, 449-454.

[S12] Gaussian 16, Revision C.01, M. J. Frisch, G. W. Trucks, H. B. Schlegel, G. E. Scuseria, M. A. Robb, J. R. Cheeseman, G. Scalmani, V. Barone, G. A. Petersson, H. Nakatsuji, X. Li, M. Caricato, A. V. Marenich, J. Bloino, B. G. Janesko, R. Gomperts, B. Mennucci, H. P. Hratchian, J. V. Ortiz, A. F. Izmaylov, J. L. Sonnenberg, D. Williams-Young, F. Ding, F. Lipparini, F. Egidi, J. Goings, B. Peng, A. Petrone, T. Henderson, D. Ranasinghe, V. G. Zakrzewski, J. Gao, N. Rega, G. Zheng, W. Liang, M. Hada, M. Ehara, K. Toyota, R. Fukuda, J. Hasegawa, M. Ishida, T. Nakajima, Y. Honda, O. Kitao, H. Nakai, T. Vreven, K. Throssell, J. A. Montgomery, Jr., J. E. Peralta, F. Ogliaro, M. J. Bearpark, J. J. Heyd, E. N. Brothers, K. N. Kudin, V. N. Staroverov, T. A. Keith, R. Kobayashi, J. Normand, K. Raghavachari, A. P. Rendell, J. C. Burant, S. S. Iyengar, J. Tomasi, M. Cossi, J. M. Millam, M. Klene, C. Adamo, R.

Cammi, J. W. Ochterski, R. L. Martin, K. Morokuma, O. Farkas, J. B. Foresman, and D. J. Fox, Gaussian, Inc., Wallingford CT, 2016.

[S13] S. Raub, G. Jansen, *Theor. Chem. Acc.* **2001**, *106*, 223-232.

[S14] A. V. Marenich, C. J. Cramer, D. G. Truhlar. *J. Phys. Chem. B* **2009**, *113*, 6378.

[S15] K. A. Peterson, D. Figgen, E. Goll, H. Stoll, M. Dolg. *J. Chem. Phys.* **2003**, *119*, 11113.

[S16] I. Kaljurand, A. Kütt, L. Sooväli, T. Rodima, V. Mäemets, I. Leito, I. A. Koppel. *J. Org. Chem.* **2005**, *70*, 1019.

[S17] M. K. Chantooni, I. M. Kolthoff. *J. Phys. Chem.* **1975**, *19*, 1176.

[S18] *Gaussian 09, Revision B.01*, M. J. Frisch, G. W. Trucks, H. B. Schlegel, G. E. Scuseria, M. A. Robb, J. R. Cheeseman, G. Scalmani, V. Barone, B. Mennucci, G. A. Petersson, H. Nakatsuji, M. Caricato, X. Li, H. P. Hratchian, A. F. Izmaylov, J. Bloino, G. Zheng, J. L. Sonnenberg, M. Hada, M. Ehara, K. Toyota, R. Fukuda, J. Hasegawa, M. Ishida, T. Nakajima, Y. Honda, O. Kitao, H. Nakai, T. Vreven, J. A. Montgomery, Jr., J. E. Peralta, F. Ogliaro, M. Bearpark, J. J. Heyd, E. Brothers, K. N. Kudin, V. N. Staroverov, R. Kobayashi, J. Normand, K. Raghavachari, A. Rendell, J. C. Burant, S. S. Iyengar, J. Tomasi, M. Cossi, N. Rega, J. M. Millam, M. Klene, J. E. Knox, J. B. Cross, V. Bakken, C. Adamo, J. Jaramillo, R. Gomperts, R. E. Stratmann, O. Yazyev, A. J. Austin, R. Cammi, C. Pomelli, J. W. Ochterski, R. L. Martin, K. Morokuma, V. G. Zakrzewski, G. A. Voth, P. Salvador, J. J. Dannenberg, S. Dapprich, A. D. Daniels, Ö. Farkas, J. B. Foresman, J. V. Ortiz, J. Cioslowski, and D. J. Fox, Gaussian, Inc., Wallingford CT, 2010.

[S19] (a) E. Raamat, K. Kaupmees, G. Ovsjannikov, A. Trummal, A. Kütt, J. Saame, I. Koppel, I. Kaljurand, L. Lipping, T. Rodima, V. Pihl, I.A. Koppel, I. Leito *J. Phys. Org. Chem.* **2013**, *26*, 162. (b) J. Ho, A. Klamt, M. Coote, *J. Phys. Chem. A* **2010**, *114*, 13442.

[S20] K. S. Alongi, G. C. Shields In: *Annual Reports in Computational Chemistry (Volume 6)*, Ed. R.A. Wheeler, Elsevier, **2010**, 113.
